# LASER couples damage sensing to ESCRT assembly for lysosome repair

**DOI:** 10.1038/s41586-026-10604-6

**Published:** 2026-06-03

**Authors:** Claire S. Goul, Aakriti Jain, Samira Yitiz, Zahra E. Soltani, Serim Yang, Simon Rapp, Martina Spacci, Scot Federman, James Sacco, Huinan Li, Lauren D. Enriquez, Nalan Liv, Laralynne Przybyla, Roberto Zoncu

**Affiliations:** 1 Department of Molecular and Cell Biology, University of California at Berkeley, Berkeley, CA USA; 2 Laboratory for Genomics Research, San Francisco, CA USA; 3 Department of Biochemistry and Biophysics, University of California, San Francisco, San Francisco, CA USA; 4 Center for Molecular Medicine, University Medical Center Utrecht, Institute of Biomembranes, Utrecht University, Utrecht, The Netherlands; 5 Present Address: Department of Physiology, University of Texas Southwestern Medical Center, Dallas, TX USA

**Keywords:** Lysosomes, Calcium signalling, Endoplasmic reticulum

## Abstract

Lysosomal membrane integrity is essential for cell survival, but how damage sensing is spatiotemporally coupled to repair remains poorly understood. Recruitment and assembly of endosomal sorting complex required for transport (ESCRT) I–III rapidly counteracts membrane damage, but it is unclear how ESCRT-I recognizes defective lysosomal membranes. Here, leveraging genome-wide CRISPRi screens in a damage-sensitized genetic background, we identified LC3/GABARAP-assisted stimulator for ESCRT recruitment (LASER), a multicomponent protein assembly that forms rapidly upon calcium release from damaged lysosomes and couples sensing of lysosomal membrane damage to ESCRT-dependent repair. At the core of LASER is TFG, an endoplasmic reticulum exit-site-resident protein that translocates to damaged lysosomes by binding to ATG8 family proteins (LC3 and GABARAP) conjugated to lysosomal phospholipids. ATG8-bound TFG forms oligomeric assemblies that directly recruit the essential ESCRT-I subunit TSG101 via conserved motif recognition enhanced by avidity-driven interactions. TFG binding to TSG101 stimulates sequential ESCRT-I–II–III polymerization and promotes membrane repair. TFG mutations that drive hereditary spastic paraplegia disrupt its oligomerization and impair lysosomal ESCRT recruitment and membrane resealing, implicating defective repair as a driver of TFG-associated neurodegeneration. Thus, LASER promotes ESCRT polymerization at damaged lysosomes and couples damage sensing to membrane repair.

## Abstract (web summary)

ATG8 conjugation on damaged lysosomes triggers rapid assembly of a protein complex containing TFG, which directs lysosomal membrane repair by recruiting ESCRT proteins to sites of damage.

## Main

Lysosomal membrane integrity is essential for cellular homeostasis and survival, yet lysosomes are continuously exposed to stresses that threaten membrane stability, including lipid oxidation, lipid asymmetry and the accumulation of aggregation-prone luminal cargo^1^. In response to membrane disruption, cells activate a multi-tiered repair system that monitors and restores lysosomal integrity, preventing leakage of luminal contents and the resulting cell death^1^. Lysosomal membrane damage occurs along a continuum, ranging from subtle loss of ion gradients and lipid packing defects to overt membrane rupture, raising the possibility that distinct damage-sensing and repair mechanisms operate at early versus advanced stages of disruption^2–6^.

The membrane remodelling ESCRT-I, -II and -III complexes provide one of the earliest-acting repair mechanisms, undergoing sequential assembly and oligomerization on damaged lysosomes that help to prevent, stabilize or reseal membrane tears^7–10^. Accordingly, depletion of the essential ESCRT-I subunit tumor susceptibility gene 101 (TSG101) compromises ESCRT polymerization and leads to accumulation of severely damaged lysosomes^7,8^. However, despite its obligate role in membrane repair, how sensing of lysosomal damage is coupled to ESCRT recruitment remains unclear.

In contrast to intraluminal vesicle formation, in which ESCRT-0 recognizes ubiquitylated endocytosed proteins to initiate sequential ESCRT-I–II–III polymerization^11^, ESCRT-0 is dispensable for lysosomal membrane repair, and ubiquitylation is instead detected during bulk removal of irreversibly damaged lysosomes (lysophagy)^2,7,12,13^. The calcium sensor apoptosis linked gene-2 (ALG2) was proposed to recruit ESCRT to damaged lysosomes in a calcium-dependent manner^14,15^. However, in vitro and cell-based evidence suggests that the requirement for calcium-ALG2 in ESCRT polymerization at damage sites may be insult-specific and not universal^14–16^.

Recently, recruitment of ESCRT subunits and of the accessory ESCRT assembly factor ALG2-interacting protein X (ALIX) to damaged lysosomes was shown to require conjugation of ATG8 proteins to single membranes (CASM). During CASM, ATG8 proteins are conjugated to lysosomal membrane phospholipids by the E3-like ATG5–ATG12 complex, which is recruited to damaged lysosomes either by ATG16L1, bound to stalled vacuolar ATPases, or by tectonin β-propeller repeat-containing 1 (TECPR1), bound in turn to inner leaflet sphingomyelin that becomes exposed to the cytosol^15,17–21^. Despite evidence for both ATG16L1- and TECPR1-dependent CASM contributing to ESCRT-I recruitment, the specific molecular links between lysosome-bound ATG8 and ESCRT-I subunits have not been identified.

Another unresolved question is the relationship between ESCRT assembly and the extensive endoplasmic reticulum (ER)–lysosome membrane contact sites that are established upon lysosomal damage^22–24^. The proximity created by these contacts enable the ER-to-lysosome transfer of phospholipids by oxysterol binding protein-related proteins (ORPs) and bridge-like lipid transport protein (BLTP) family proteins, which together help restore the optimal topology, surface area and composition of the lysosomal limiting membrane^20,22–24^. However, whether ER–lysosome contacts have additional repair-promoting roles, specifically whether and how they contribute to the regulation of ESCRT assembly on damaged lysosomes, is currently unknown.

Neuronal lysosomes operate close to a damage threshold and are therefore uniquely reliant on efficient membrane repair^3,25^. Excess or aberrant proteolytic cargo, compounded by genetic or environmental perturbations in cholesterol and sphingolipid metabolism, accelerates membrane injury^26–28^. Increasing evidence suggests that even healthy neurons undergo constitutive, albeit transient, lysosomal permeabilization^3,5,29^. Mutations in several components of the lysosomal repair machinery are increasingly linked to neurodegenerative conditions. Most notably, the ESCRT-III subunit charged multivesicular body protein 2B (CHMP2B) is mutated in amyotrophic lateral sclerosis and frontotemporal degeneration^11,30,31^, whereas mutations in the ESCRT-I subunit ubiquitin associated protein 1 (UBAP1) underlie hereditary spastic paraplegia (HSP)^32^. However, given the pleiotropic roles of ESCRT in membrane remodelling, the extent to which defective ESCRT assembly in response to lysosomal damage drives neurodegeneration remains unclear.

Here we identify LASER and its core component Trk-fused gene (TFG) as a key molecular link between lysosomal damage sensing and ESCRT-mediated membrane repair, acting through cooperative, motif-driven interactions that promote ESCRT polymerization. By uncovering a requirement for VAMP-associated proteins (VAPs) in LASER assembly, we further reveal how ER–lysosome contact formation is coordinated with ESCRT activation during repair. Finally, we demonstrate that TFG mutations linked to HSP cause profound defects in ESCRT recruitment and lysosomal membrane resealing. Together, these findings define a molecular pathway that integrates damage detection, ESCRT assembly and membrane contact site remodelling and implicate defective lysosomal repair as a mechanistic driver of TFG-associated neurodegeneration.

## TFG is a modifier of lysosomal damage response

**Fig. 1:**
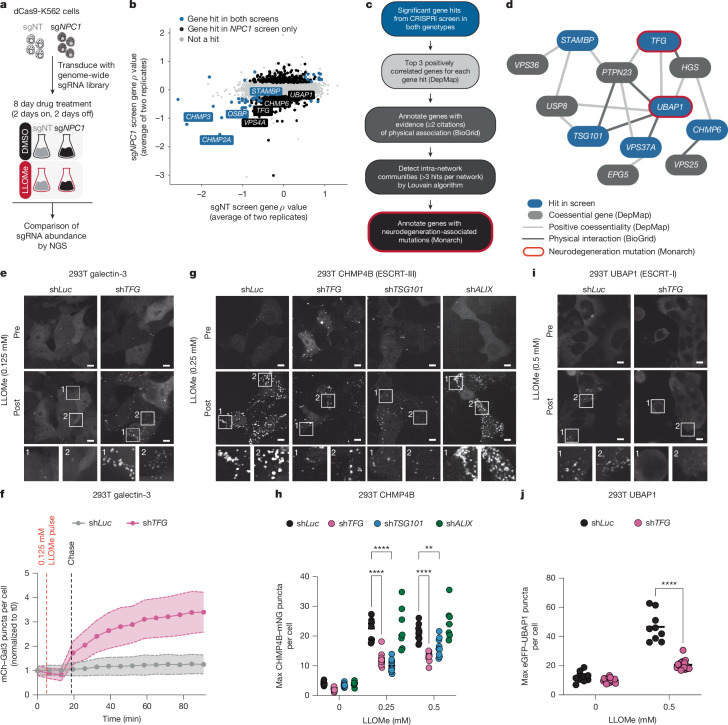
Genome wide CRISPRi screen identifies TFG as a modifier of ESCRT-mediated lysosome membrane repair. **a**, Genome-wide CRISPRi screen design. Cells underwent an 8-day regimen of two 4-day cycles: 2 days of LLOMe (0.5 mM) followed by 2 days without drug. **b**, Scatter plot of hits (*P* < 0.05, blue/black) with lipid transport (*OSBP*), ESCRT-0 (*STAMBP*), ESCRT-I (*UBAP1*), ESCRT-III (*CHMP2A*, *CHMP3*, *CHMP6*) genes labelled. sg*NPC1*, sgRNA targeting *NPC1*; sgNT, non-targeting control sgRNA. **c**, Network analysis pipeline to identify functional modules. **d**, ESCRT-0, ESCRT-I and ESCRT-II subnetwork containing hits (blue) and coessential genes (grey), with protein–protein interactions (dark grey edges) and neurodegenerative mutations (red outline). **e**,**f**, Representative images (**e**; *t* = 90 min) and quantification (**f**) of mCherry–Gal3 (mCh–Gal3) recruitment to lysosomes upon pulse–chase with 0.125 mM LLOMe following treatment with sh*TFG* or luciferase control (sh*Luc*). Each point shows the mean number of mCh–Gal3 puncta per cell (*n* = 8 fields of view (FOVs)) and the shaded region indicates s.d. **g**,**h**, Representative images (**g**; *t* = 5 min) and quantification of the maximum (max) number (**h**) of CHMP4B–mNG puncta per cell across the time course in 293T cells treated with DMSO (vehicle) or 0.25 mM or 0.5 mM LLOMe following treatment with sh*TFG* (pink), sh*TSG101* (blue), sh*ALIX* (green) or sh*Luc* (black). Each point represents one FOV (*n* = 10). *ALIX* is also known as *PDCD6IP*. **i**,**j**, Representative images (**i**; *t* = 60 min) and quantification of the maximum number (**j**) of eGFP–UBAP1 puncta per cell across the time course in 293T cells treated with DMSO or 0.5 mM LLOMe following treatment with sh*TFG* (pink) or sh*Luc* (black). Each point represents one FOV (*n* = 9). Comparison of groups by two-sided Mann–Whitney test (**b**) or two-way ANOVA (**h**,**j**). **h**, 0.25 mM, sh*Luc* versus sh*TFG*: *P* = 8.5 × 10^−6^; sh*Luc* versus sh*TSG101*: *P* = 1.2 × 10^−6^; 0.5 mM, sh*Luc* versus sh*TFG*: *P* = 7.0 × 10^−6^; sh*Luc* versus sh*TSG101*: *P* = 5.8 × 10^−3^. **j**, *P* = 9.4 × 10^−12^. Scale bars, 10 µm. *P < 0.05, **P < 0.01, ***P < 0.001, ****P < 0.0001; NS, not significant (*P* ≥ 0.05). Source data

**Extended Data Fig. 1:**
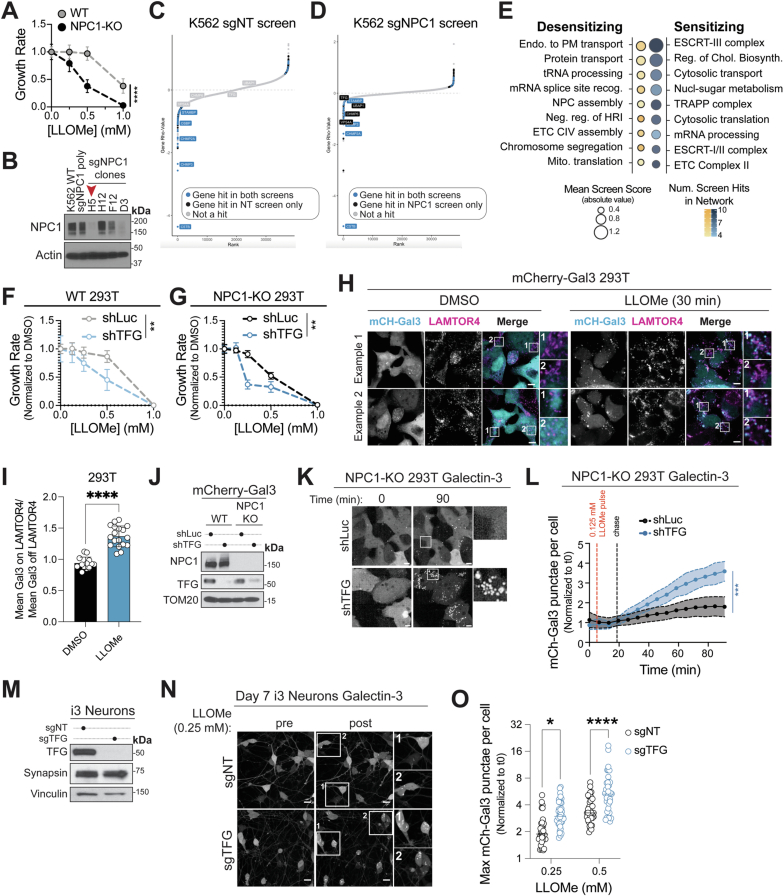
Genome wide CRISPRi screen identifies TFG as a new modifier of lysosome membrane repair. (**A**) Growth rate of wild-type (WT) or NPC1 knockout (KO) 293 T cells treated with shRNAs against Luciferase (Luc) control or TFG. Cells were grown in the presence of DMSO (vehicle control) or 0.25, 0.5, or 1 mM LLOMe. Quantification of 3 replicates per group. (**B**) Western blot of WT, sgNPC1 polyclonal, or sgNPC1 clonal K562 cell lines. Growth curves with sgNPC1 clonal K562 lines were repeated 3 times. (**C, D**) Individual waterfall plot of screen hits (significant, ρ < 0.05, in blue/black) from sgNT (C) and sgNPC1 (D) screens. (**E**) Bubble plot showing the top 9 unique enriched subnetworks identified from Louvain algorithm among sensitizing (blue) or desensitizing (yellow) hits, ordered by mean score of screen hits in community. Comparison of groups carried out by two-way ANOVA (**A**). (**F, G**) Growth rate of WT (**F**) or NPC1-KO (**G**) 293 T cells treated with shRNAs against Luc control or TFG. Cells were grown in the presence of DMSO (vehicle control) or 0.125, 0.25, 0.5, or 1 mM LLOMe. Quantification of 3 replicates per group. (**H, I**) Representative images (**H**) of mCherry-Galectin-3 (Gal3) and LAMTOR4 (lysosomal marker) immunofluorescence staining and quantification (**I**) of colocalization ratio between mCherry-Gal3 puncta and LAMTOR4 in 293 T cells. Cells were treated with vehicle control (DMSO) or 0.25 mM LLOMe for 30 mins. Quantification of 18 individual cells. (**J**) Western blot analysis of WT or NPC1-KO mCherry-Gal3 expressing 293 T cells following knockdown with shRNAs against Luc control or TFG. TFG knockdown experiments in Gal3-mCherry cells were repeated at least 3 times. (**K, L**) Representative images (**K**) and quantification (**L**) of mCherry-Gal3 expressing NPC1-KO 293 T cells treated with shRNAs against Luc control or TFG and treated with a pulse of 0.125 mM LLOMe followed by a chase. Shaded region within dotted lines is standard deviation. Quantification of 8 fields of view (FOVs). Scale bars, 10 µm. (**M**) Western blot analysis i3 neurons treated with single guide RNAs against TFG (sgTFG) or non-against control (sgNT). Experiments with sgNT and sgTFG in i3neurons were repeated 2 times. (**N, O**) Representative images (**N**) and quantification (**O**) of mCherry-Gal3 puncta formation upon treatment with DMSO vehicle control or 0.25 mM or 0.5 mM LLOMe in i3 neurons following knockdown of TFG (sgTFG) or non-against control (sgNT). Each point is one FOV (n = 41). Images are shown for 45 min of treatment. Scale bars, 20 µm. Comparison of groups carried out by unpaired two sided t-test (**I**) or two-way ANOVA (**A, F, G, L, O**) (**A:** 0.25 mM, p = 7.6 × 10^−3^; 0.5 mM, p = 4.8 × 10^−8^; 1 mM, p = 3.9 × 10^−5^; **F:** 0 vs 0.5 mM, p = 5.5 × 10^−3^; **G:** 0 vs 0.25 mM: p = 6.3 × 10^−5^**; I**: p = 5.3 × 10^−10^; **L**: p = 1 × 10^−15^ (t = 91), p = 3.3 × 10^−14^ (t = 84.5), p = 1.7 × 10^−12^ (t = 78), p = 6.8 × 10^−11^ (t = 71.5), p = 4.2 × 10^−9^ (t = 65), p = 2.9 × 10^−7^ (t = 58.5), p = 4.3 × 10^−5^ (t = 52); **O:** 0.25 mM: p = 3.2 × 10^−2^, 0.5 mM: p = 1.7 × 10^−6^). Error bars represent mean +/− standard deviation. ****p < 0.0001, ***p < 0.001, **p < 0.01, *p < 0.05. Source data

In cells that lack the lysosomal cholesterol transporter Niemann–Pick type C1 (NPC1), lysosomes have increased propensity to damage upon treatment with the lysosomotropic reagent, l-leucyl-l-leucine methyl-ester (LLOMe)^26,33^. Consistent with increased damage, *NPC1*-knockout HEK293T cells are more sensitive than control cells to LLOMe-mediated growth inhibition (Extended Data Fig. 1a). Thus, *NPC1*-knockout cells represent a sensitized background in which to identify genetic modifiers of lysosome membrane damage and repair. To this end, we designed parallel, growth-based, whole-genome CRISPR inhibition (CRISPRi) screens in control and *NPC1*-deleted K562 cells to identify genes that modify survival in the presence of an LLOMe challenge (Fig. 1a and Extended Data Fig. 1b). Control and *NPC1*-knockout K562 cells stably expressing CRISPRi–dCas9 machinery were transduced with a genome-wide single guide RNA (sgRNA) library with coverage of five guides per gene. Both cell populations were challenged for 8 days with LLOMe at a concentration resulting in half-maximal growth inhibition, followed by next-generation sequencing (Fig. 1a). After thresholding for statistically significant gene hits across control and *NPC1*-knockout cells, we identified 389 genes that, when knocked down, sensitize cells to LLOMe (sensitizing hits, indicating a damage-protective gene function) and 322 desensitizing gene hits. Consistent with their role in membrane repair, depletion of multiple components of the ESCRT-I and ESCRT-III complexes was strongly sensitizing in both genotypes (Fig. 1b, Extended Data Fig. 1c,d and Supplementary Table 1). Notably, the *NPC1*-knockout cells yielded the majority of both sensitizing and desensitizing hits (65% and 77%, respectively), consistent with the damage-prone state of this genetic background.

**Extended Data Fig. 2:**
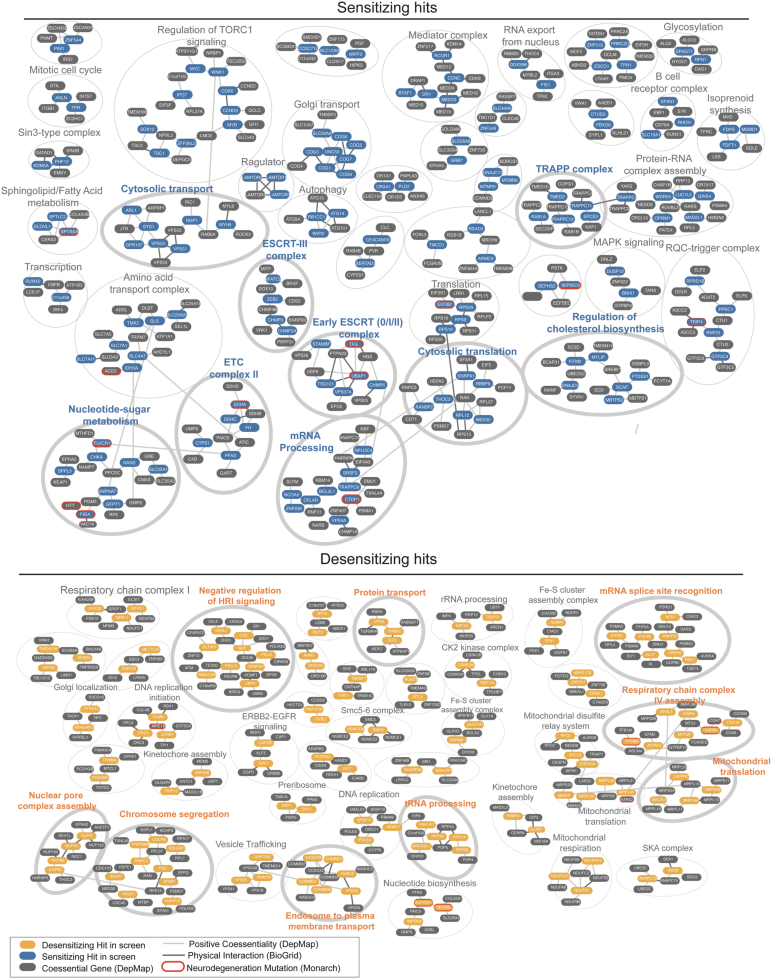
Coessentiality networks of CRISPRi screen hits. Coessentiality networks of sensitizing (***top***) or desensitizing (***bottom***) hits (blue or yellow nodes, respectively) from sgNT and sgNPC1 screens. Functional subnetworks are annotated (gray circles) with corresponding top Gene Ontology terms (mean scores of hit nodes in the screen per subnetwork was calculated, and the top 9 scoring subnetworks are annotated in blue or orange text and bolded gray circles).

To prioritize gene hits for functional characterization, we built a bioinformatic pipeline that clusters hits into functionally related networks based on coessentiality, overlaid with evidence for physical protein–protein interaction and for neurodegenerative disease association (Fig. 1c). Inspection of the sensitizing networks revealed several functional modules including lysosomal membrane repair (ESCRTs and autophagy), vesicle trafficking (transport protein particle (TRAPP), conserved oligomeric Golgi (COG) complex), and cholesterol and phospholipid homeostasis (Extended Data Figs. 1e and 2). By contrast, the desensitizing hits included growth regulation (mitochondrial translation, tRNA processing and HRI (also known as eIF-2α kinase) signalling) and endosome and lysosome biogenesis (COMMD and homotypic fusion and protein sorting (HOPS) complex) (Extended Data Figs. 1e and 2).

The network analysis revealed novel candidate protective genes within networks of known function. In particular, the ESCRT-I–II network contained TFG, a protein not previously associated with lysosomal repair (Fig. 1d). We proceeded to validate TFG as protective against lysosome damage by measuring the growth of TFG-depleted HEK293T cells under LLOMe challenge. In both control and *NPC1*-knockout genotypes, *TFG* knockdown with small hairpin RNA (shRNA) led to decreased growth rate upon LLOMe treatment compared with shRNA targeting luciferase (sh*Luc*) (Extended Data Fig. 1f,g).

To test whether the reduced growth of TFG-depleted cells was due to failure to repair damaged lysosomes, we assessed LLOMe-induced recruitment of mCherry–galectin 3 (Gal3), a readout for lysosomal membrane permeabilization^7,8^. In both control and *NPC1*-knockout genotypes, we found increased Gal3 recruitment in cells treated with shRNA targeting *TFG* (sh*TFG*) compared with sh*Luc*-treated cells (Fig. 1e,f and Extended Data Fig. 1h–l).

Loss-of-function mutations in *TFG* drive several neurological disorders, including HSP, Charcot–Marie–Tooth disease, hereditary motor and sensory neuropathy (HMNS-P) and amyotrophic lateral sclerosis^34^. Consistent with a neuroprotective function of TFG, its CRISPRi-mediated depletion in induced pluripotent stem cell (iPSC)-derived neurons led to increased damage upon LLOMe treatment, as assessed by higher numbers of mCherry–Gal3 puncta compared with control neurons (Extended Data Fig. 1m–o). It is worth emphasizing that although TFG scored as a hit only in the *NPC1*-knockout arm of the CRISPRi screen (Extended Data Fig. 1c,d), its depletion in follow-up experiments sensitized both control and *NPC1*-knockout HEK293T cells to LLOMe, highlighting the general requirement for TFG in lysosomal membrane homeostasis.

## TFG is required for ESCRT recruitment to damaged lysosomes

**Extended Data Fig. 3:**
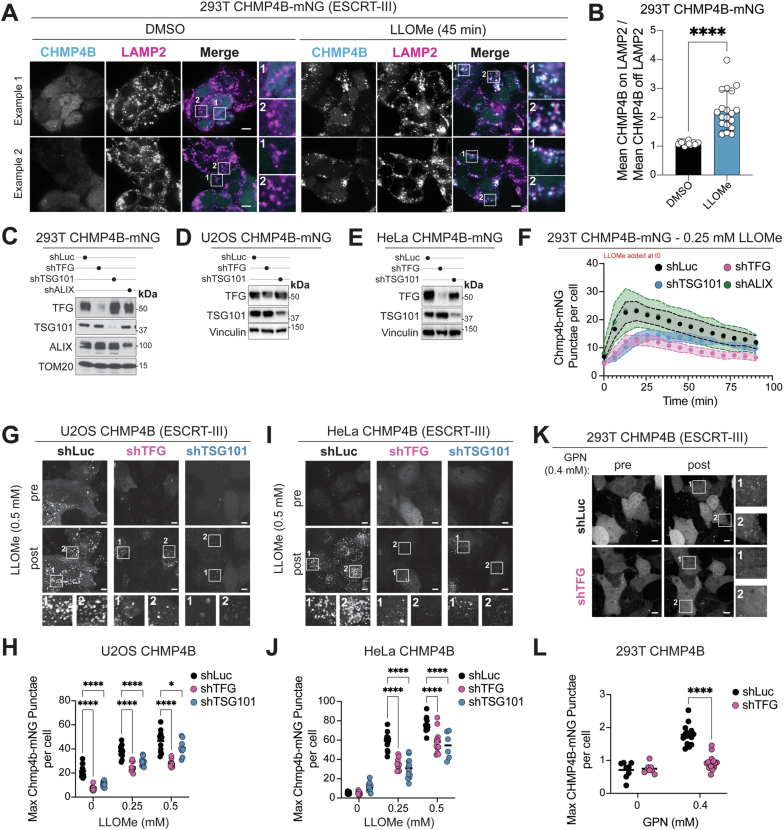
TFG loss results in decreased ESCRT-III recruitment to damaged lysosomes. (**A, B**) Representative images (**A**) and quantification (**B**) of CHMP4B-mNeonGreen (mNG) and LAMP2 colocalization in 293 T cells treated with vehicle (DMSO) or 0.25 mM LLOMe for 45 min (n = 20 cells). (**C-E**) Western blots of 293 T (**C**), U2OS (**D**), or HeLa (**E**) expressing CHMP4B-mNeonGreen treated with shRNA against Luc control, TFG, or TSG101. Experiments with CHMP4B-mNeonGreen cells were repeated at least 3 times. (**F**) CHMP4B-mNG puncta formation in 293 T treated with shRNAs against Luc, TFG, or TSG101 and 0.25 mM LLOMe. Each point is the mean. Quantification of 10 FOVs. (**G, H**) Representative images (**G**) and quantification (**H**) of maximum CHMP4B-mNG puncta across the time course in U2OS treated with DMSO, 0.25 mM or 0.5 mM LLOMe and shRNA against TFG (pink), TSG101 (blue), or Luc (black). Images are of 15 min of treatment. Each point is one FOV (n = 12, except n = 9 for shTSG101 0.5 mM). (**I, J**) Representative images (**I**) and quantification (**J**) of maximum CHMP4B-mNG puncta across the time course in HeLa treated with DMSO, 0.25 mM or 0.5 mM LLOMe and shRNA against TFG (pink), TSG101 (blue), or Luc (black). Images are of 20 min of treatment. Each point is one FOV (n = 12, except n = 6 for shTSG101 0.5 mM and n = 9 for shTFG 0.25 mM), Scale bars, 10 µm. (**K, L**) Representative images (**K**) and quantification (**L**) of maximum CHMP4B-mNG puncta in 293 T treated with DMSO or 0.4 mM GPN following shRNA against TFG (pink) or Luc (black). Images are of 5 min of treatment. Each point is one FOV (n = 8). Comparisons by two-sided unpaired t-test (**B**) or two-way ANOVA (**H, J, L**) (**B:** p = 7.4 × 10^−6^, **H:** 0 mM shLuc vs shTFG: p = 4.7 × 10^−10^, shLuc vs shTSG101: p = 1.9 × 10^−6^; 0.25 mM shLuc vs shTFG: p = 6.6 × 10^−9^, shLuc vs shTSG101, p = 1.0 × 10^−4^; 0.5 mM shLuc vs shTFG: p = 9 × 10^−15^, shLuc vs shTSG101, p = 5.0 × 10^−2^; **J**: 0.25 mM shLuc vs shTFG: p = 1.6 × 10^−8^, shLuc vs shTSG101: p = 2.3 × 10^−12^; 0.5 mM shLuc vs shTFG: p = 3.8 × 10^−6^, shLuc vs shTSG101 p = 5.8 × 10^−6^; **L:** 0.4 mM GPN**, p** = 1.2 × 10^−12^). Error bars are mean +/− standard deviation. ****p < 0.0001, *p < 0.05. Scale bars, 10 µm. Source data

TFG was shown to localize to ER exit sites (ERES), where it regulates COPII vesicle assembly and turnover^34,35^. Notably, TFG has also been linked to the sorting of internalized plasma membrane receptors into multivesicular bodies (MVBs), an ESCRT-dependent process^36^. The presence of TFG in an ESCRT network prompted us to investigate whether TFG is required for ESCRT recruitment to damaged lysosomes. We generated HEK293T, HeLa and U2OS cells that stably expressed the ESCRT-III subunit CHMP4B–mNeonGreen (CHMP4B–mNG), which, as expected, accumulated on LAMP2-positive lysosomes upon LLOMe-induced damage^7^ (Extended Data Fig. 3a,b). Depletion of TFG impaired lysosomal CHMP4B–mNG recruitment to a similar degree to depletion of the obligate ESCRT-I component TSG101, whereas depleting ALIX did not affect CHMP4B–mNG recruitment (Fig. 1g,h and Extended Data Fig. 3c–j). TFG depletion similarly reduced CHMP4B–mNG recruitment following treatment with a distinct lysosomotropic agent, glycyl-L-phenylalanine 2-naphthylamide (GPN)^14^, indicating a broad role of TFG in ESCRT-III assembly at damaged lysosomes (Extended Data Fig. 3k,l).

**Extended Data Fig. 4:**
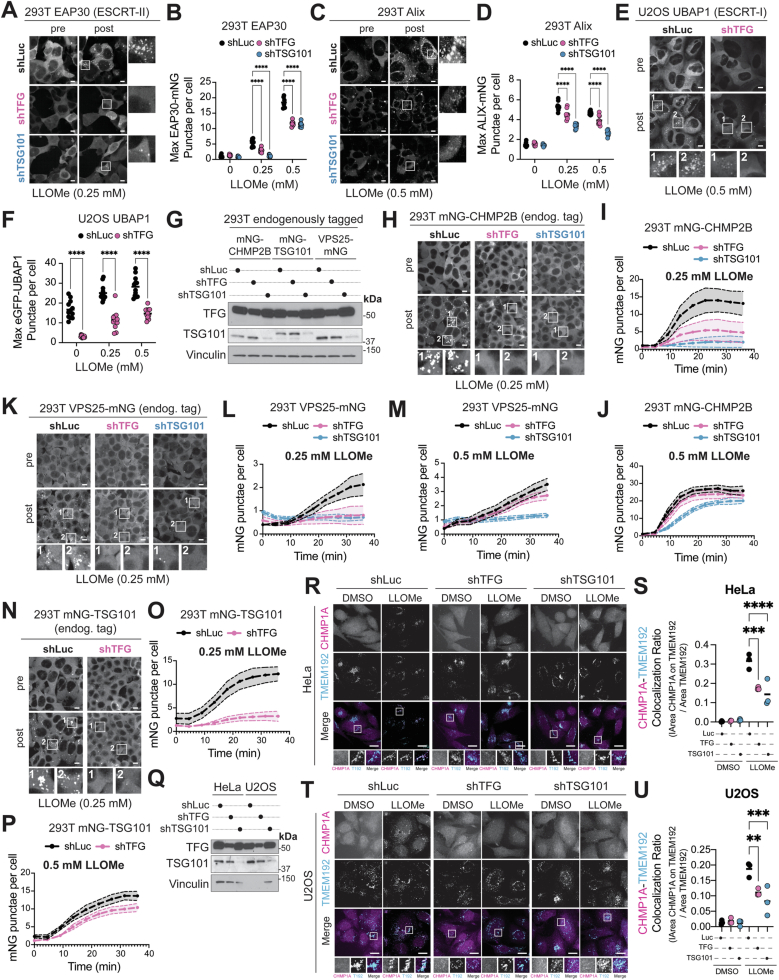
TFG loss results in decreased ESCRT-I, II, III, and ALIX recruitment to damaged lysosomes. (**A, B**) Representative images (**A**) and quantification (**B**) of maximum EAP30-mNG puncta formed across the time course in 293 T cells treated with DMSO (vehicle control), 0.25 mM or 0.5 mM LLOMe. Cells were treated with shRNA against TFG (pink) or TSG101 (blue) compared to Luc control (black). Images are shown for 60 min of treatment. Each point is one FOV (n = 8). (**C, D**) Representative images (**C**) and quantification (**D**) of maximum Alix-mNG puncta formed in 293 T cells treated with DMSO, 0.25 mM or 0.5 mM LLOMe following treatment with shRNA against TFG (pink) or TSG101 (blue) compared to Luc control (black). Images are shown for 15 min of treatment. Each point is one FOV (n = 10). (**E, F**) Representative images (**E**) and quantification (**F**) of maximum eGFP-UBAP1 puncta formed in U2OS cells treated with DMSO, 0.25 mM or 0.5 mM LLOMe following treatment with shRNA against TFG (pink) compared to Luc control (black). Images are shown for 60 min of treatment. Each point is one FOV (n = 12). (**G**) Western blot analysis of 293 T cells tagged at the endogenous locus for CHMP2B, TSG101, or VPS25 with mNG following knockdown with shRNAs against Luc control, TFG, or TSG101. Experiments in these cell lines with Luc, TFG, or TSG101 knockdown were repeated 2 times. (**H-P**) Representative images (**H, K, N**) and quantification (**I, J, L, M, O, P**) of maximum mNG-CHMP2B (**H-J**), VPS25-mNG (**K-M**), or mNG-TSG101 (**N-P**) puncta formed in endogenously tagged 293 T cells treated with either 0.25 mM (**H, I, K, L, N, O**) or 0.5 mM (**J, M, P**) LLOMe following treatment with shRNA against TFG (pink) or TSG101 (blue) compared to Luc control (black). Images are shown for 20 min of treatment. Each point is one FOV (n = 12). (**Q**) Western blot analysis of Hela and U2OS cells following knockdown with shRNAs against Luc control, TFG, or TSG101. HeLa and U2OS ESCRT colocalization experiments were repeated 2 times. (**R-U**) Representative immunofluorescence images (**R, T**) and quantification (**S, U**) of colocalization between CHMP1A and TMEM192 in HeLa (**R, S**) or U2OS (**T, U**) cells treated with vehicle control (DMSO) or 0.5 mM LLOMe. Cells were treated with shRNAs against Luc control, TFG, or TSG101. Each point is one FOV. Quantification of n = 3 FOVs. Comparison of groups carried out by two-way ANOVA (**B, D, F**) or one-way ANOVA (**S, U**). ****p < 0.0001, ***p < 0.001. Scale bars, 10 µm. Error bars represent mean +/− standard deviation (I, L, M, J, O, P) (**B**: 0.25 mM shLuc vs shTFG, p = 4.3 × 10^−5^; shLuc vs shTSG101, p = 6.5 × 10^−12^; 0.5 mM shLuc vs shTFG, p < 1 × 10^−15^, shLuc vs shTSG101 p < 1 × 10^−15^; **D**: 0.25 mM shLuc vs shTFG, p = 2.9 × 10^−7^, shLuc vs shTSG101, p < 1 × 10^−15^; 0.5 mM shLuc vs shTFG, p = 9.3×10^−7^; shLuc vs shTSG101, p < 1 × 10^−15^; **F**: 0 mM p = 1.7 × 10^−11^, 0.25 mM p = 1.1 × 10^−11^, 0.5 mM p = 1.6 × 10^−11^; **S**: LLOMe shLuc vs shTFG, p = 3.5 × 10^−3^, shLuc vs shTSG101 p = 2.7 × 10^−4^; **U**: shLuc vs shTFG LLOMe p = 3.9 × 10^−4^, shLuc vs shTSG101 p = 6.7 × 10^−5^). Source data

We next investigated the role of TFG in earlier ESCRT assembly steps. ESCRT-II recruits ESCRT-III subunits, initiating ESCRT-III filament polymerization and subsequent membrane remodelling^11^. Consistent with a requirement in ESCRT-II assembly, TFG depletion impaired recruitment of the ESCRT-II subunit EAP30 (also known as VPS22)–mNG to damaged lysosomes, to a degree comparable to the effect of TSG101 depletion (Extended Data Fig. 4a,b). Accumulation of ALIX at damaged lysosomes was also TFG-dependent (Extended Data Fig. 4c,d). The requirement for TFG extended to ESCRT-I, as shown by the lack of lysosomal recruitment of eGFP–UBAP1 in TFG-depleted HEK293T and U2OS cells (Fig. 1i,j and Extended Data Fig. 4e,f).

We next examined the TFG requirement in HEK293T cells in which TSG101, VPS25 and CHMP2B (ESCRT-I, ESCRT-II and ESCRT-III, respectively) are tagged at the endogenous loci with mNG. mNG-tagged TSG101, VPS25 and CHMP2B accumulated at damaged lysosomes and, consistent with the exogenous ESCRT reporters, their recruitment was abolished by TFG depletion (Extended Data Fig. 4g–p). Increasing the LLOMe concentration reduced the requirement for TFG in the recruitment of these ESCRT subunits, as well as the dependency on TSG101 for CHMP2B recruitment, possibly pointing to alternative modes of ESCRT-III recruitment as the extent of damage increases^1,15,18^.

To further validate the requirement for TFG in ESCRT-III polymerization on damaged lysosomes, we carried out immunofluorescence co-staining for endogenous CHMP1A (ESCRT-III) and the lysosomal marker TMEM192. In both HeLa cells and U2OS cells, shRNA-mediated depletion of TFG resulted in decreased accumulation of CHMP1A on damaged lysosomes, to a similar extent to TSG101 depletion (Extended Data Fig. 4r–u).

**Extended Data Fig. 5:**
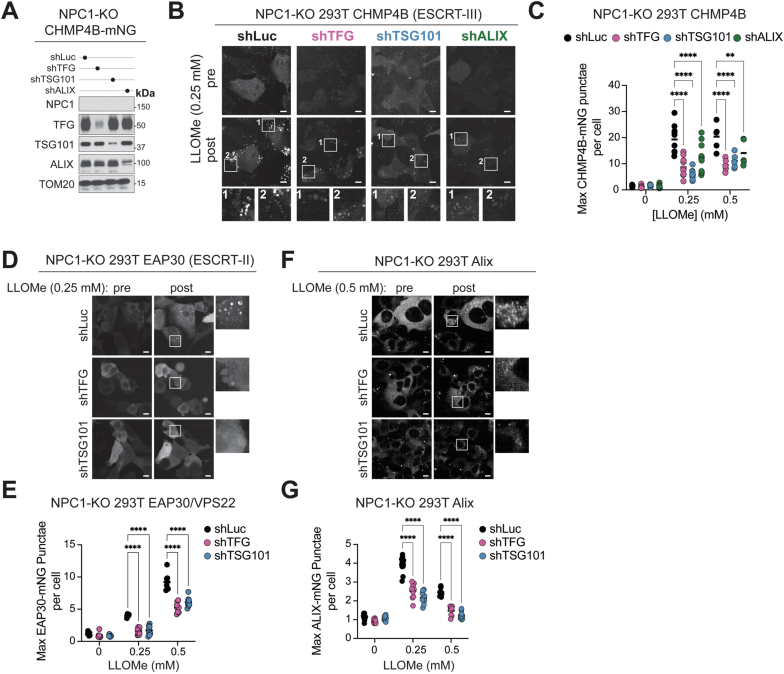
TFG loss results in decreased ESCRT recruitment to damaged lysosomes in NPC1-KO cells. (**A**) Western blot analysis of NPC1-KO 293 T cells following knockdown with shRNAs against Luc control, TFG, TSG101, or ALIX. Experiments with CHMP4B-mNG NPC1 KO cells were repeated at least 3 times. (**B, C**) Representative images (**B**) and quantification (**C**) of maximum CHMP4B-mNG puncta per cell across the time course in NPC1-KO 293 T cells treated with DMSO (vehicle control), 0.25 mM or 0.5 mM LLOMe. Cells were treated with shRNA against Luc control (black), TFG (pink) or TSG101 (blue), or ALIX (green). Each point is one FOV (n = 10, except n = 5 for 0.5 mM LLOMe). Images are shown for 20 min of treatment. (**D, E**) Representative images (**D**) and quantification (**E**) of maximum EAP30-mNG puncta per cell across the time course in NPC1-KO 293 T cells treated with DMSO (vehicle control), 0.25 mM or 0.5 mM LLOMe. Cells were treated with shRNA against Luc control (black), TFG (pink) or TSG101 (blue). Each point is one FOV (n = 8). Images are shown for 60 min of treatment. (**F, G**) Representative images (**F**) and quantification (**G**) of maximum ALIX-mNG puncta per cell across the time course in NPC1-KO 293 T cells treated with DMSO (vehicle control), 0.25 mM or 0.5 mM LLOMe. Cells were treated with shRNA against Luc control (black), TFG (pink) or TSG101 (blue). Each point is one FOV (n = 10). Images are shown for 15 min of treatment. Comparison of groups carried out by two-way ANOVA (**C, E, G**). ****p < 0.0001, ***p < 0.001, **p < 0.01 (**C:** 0.25 shLuc vs shTFG p = 4.8 × 10^−10^, shLuc vs shTSG101 p = 7.8 × 10^−14^, shLuc vs shALIX p = 4.8 × 10^−5^; 0.5 mM shLuc vs shTFG p = 3.4 × 10^−6^, shLuc vs shTSG101 p = 6.9 × 10^−5^, shLuc vs shALIX p = 9.1 × 10^−3^, **E**: 0.25 mM shLuc vs shTFG p = 1.0 × 10^−6^, shLuc vs shTSG101 p = 3.7 × 10^−6^; 0.5 mM shLuc vs shTFG p < 1 × 10^−15^, shLuc vs shTSG101 p = 4.5 × 10^−12^; **G**: 0.25 mM shLuc vs shTFG p < 1 × 10^−15^, shLuc vs shTSG101 p < 1 × 10^−15^; 0.5 mM shLuc vs shTFG p = 1.1 × 10^−11^, shLuc vs shTSG101 p < 1 × 10^−15^). Scale bars, 10 µm. Source data

Similar to wild-type cells, and consistent with the identification of TFG in the sg*NPC1* background, *NPC1*-knockout cells exhibited strong defects in ESCRT recruitment to damaged lysosomes upon TFG or TSG101 depletion (Extended Data Fig. 5a–g).

Collectively, these data identify TFG as a critical factor upstream of ESCRT-I and thus as having a role early in the ESCRT assembly cascade that enables lysosomal membrane repair.

## TFG translocates from ERES to damaged lysosomes

**Extended Data Fig. 6:**
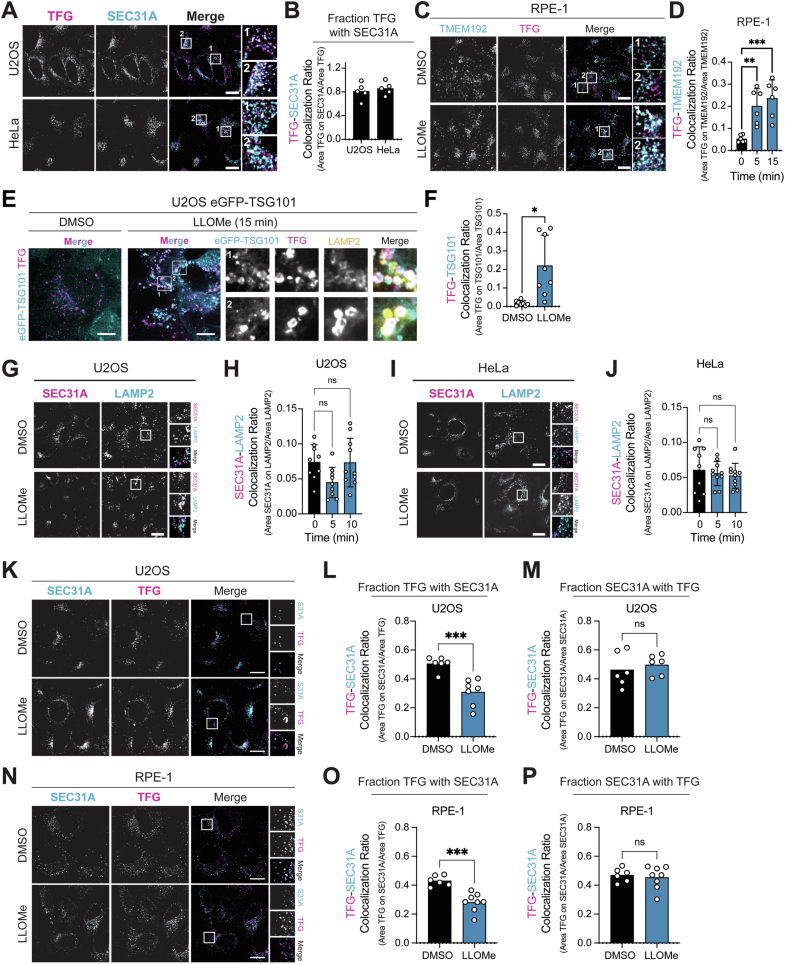
TFG translocates from ERES to damaged lysosomes. (**A, B**) Representative images of TFG and SEC31A immunofluorescence staining (**A**) and quantification (**B**) of the fraction of TFG colocalized with SEC31A in U2OS and HeLa cells. Each point is one FOV (n = 5). Scale bars, 10 µm. (**C, D**) Representative images of TFG and TMEM192 immunofluorescence staining (**A**) and quantification (**B**) of the fraction of TFG colocalized with TMEM192 in RPE-1 cells. Each point is one FOV (n = 6). Scale bars, 10 µm. (**E, F**) Representative images (**E**) of TFG, eGFP-TSG101, and LAMP2 immunofluorescence staining and quantification (**F**) of colocalization ratio between TFG and eGFP-TSG101 in U2OS cells expressing eGFP-TSG101. Cells were treated with DMSO or 0.5 mM LLOMe for 15 min. Quantification of 8 FOVs. Scale bars, 5 µm. (**G-J**) Representative images of LAMP2 and SEC31A immunofluorescence staining (**G, I**) and quantification (**H, J**) of the fraction of SEC31A colocalized with LAMP2 in U2OS (**G, H**) or HeLa (**I, J**) cells following treatment with DMSO (vehicle control) or 0.5 mM LLOMe for 10 min. Each point is one FOV (n = 10). Scale bars, 10 µm. (**K-P**) Representative images in U2OS (**K-M**) or RPE-1 (**N-P**) cells of TFG and SEC31A immunofluorescence staining (**K, N**) and quantification of the fraction of TFG colocalized with SEC31A (**L, O**) or the fraction of SEC31A colocalized with TFG (**M, P**) following treatment with DMSO or 0.5 mM LLOMe for 10 min. Each point is one FOV (n = 6 for U2OS and n = 8 for RPE-1). Scale bars, 10 µm. Comparison of groups carried out by one-way ANOVA (**D, H, J**) or two-sided Welch’s unpaired t-test (**F, L, M, O, P**) (**D:** DMSO vs 5 min, p = 3.1 × 10^−3^; DMSO vs 15 min, p = 5.2 × 10^−4^; **F**: p = 1.0 × 10^−2^, **L**: p = 7.2 × 10^−4^; **O**: p = 1.6 × 10^−4^). Error bars represent mean +/− standard deviation. ****p < 0.0001, ***p < 0.001, *p < 0.05, ns = not signficant. Source data

Our finding that TFG promotes ESCRT recruitment to damaged lysosomes motivated us to test whether TFG physically associates with these organelles. At baseline, immunofluorescence staining in U2OS and HeLa cells revealed endogenous TFG as discrete spots, about 80% of which colocalized with the ERES marker SEC31A (Extended Data Fig. 6a,b).

**Fig. 2:**
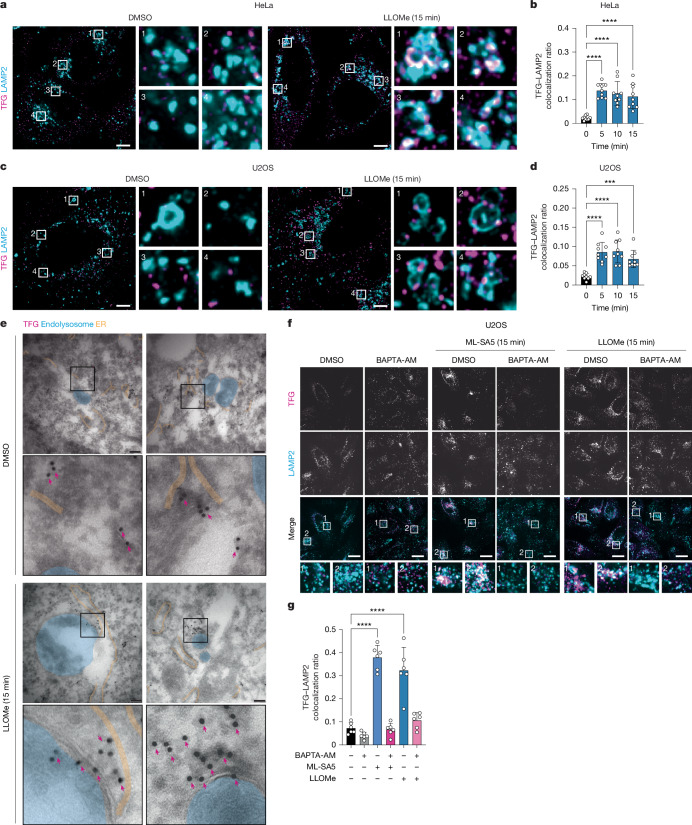
TFG translocates from ERES to damaged lysosomes. **a**–**d**, Representative images (**a**,**c**) and quantification (**b**,**d**) of colocalization ratio of TFG and LAMP2 in HeLa (**a**,**b**) or U2OS (**c**,**d**) cells treated with DMSO (vehicle control) or 0.5 mM LLOMe. Colocalization ratio is measured as area of colocalized signals divided by the area of LAMP2 signal. Each point represents one FOV (*n* = 9). Images are shown for 15 min of treatment. Scale bars, 5 µm. **b**, 0 versus 5 min: *P* = 1.5 × 10^−6^; 0 versus 10 min: *P* = 9.1 × 10^−6^; 0 versus 15 min: *P* = 7.3 × 10^−5^. **d**, 0 versus 5 min: *P* = 4.9 × 10^−6^; 0 versus 10 min: *P* = 2.9 × 10^−6^; 0 versus 15 min: *P* = 5.8 × 10^−6^. **e**, Representative EM–IGS images of TFG in HeLa cells treated with DMSO or 0.5 mM LLOMe. Images are shown for 15 min of treatment. Pink arrows indicate immuno-gold labeled TFG. Scale bars, 150 nm. Experiment was repeated three times. **f**,**g**, Representative confocal images of TFG and LAMP2 immunofluorescence staining (**f**) and quantification of colocalization ratio between TFG and LAMP2 (**g**) in U2OS cells. Cells were treated with DMSO or 20 µM BAPTA-AM, followed by treatment with either 4 µM ML-SA5 or 0.5 mM LLOMe for 15 min. Each point represents one FOV (*n* = 5). Scale bars, 10 µm. Comparison of groups carried out by one-way ANOVA (**b**,**d**,**g**). Data are mean ± s.d. **g**, Untreated versus ML-SA5, *P* = 8.2 × 10^−11^; Untreated versus LLOMe, *P* = 7.8 × 10^−9^. Source data

Remarkably, within 5 min of LLOMe treatment in multiple cell lines, TFG extensively colocalized with lysosomes (Fig. 2a–d and Extended Data Fig. 6c,d), which, as expected, also accumulated eGFP–TSG101 (Extended Data Fig. 6e,f). This result was supported by immunogold staining coupled with electron microscopy (EM–IGS) in HeLa cells, which showed TFG-positive gold particles clustered around damaged lysosomes (Fig. 2e). TFG accumulation could reflect the formation of membrane contact sites between ERES and damaged lysosomes or, alternatively, the translocation of TFG itself from ERES to the lysosomal membrane. The following observations support the second scenario: (1) there was no increase in SEC31A colocalization with lysosomes upon LLOMe treatment (Extended Data Fig. 6g–j); and (2) TFG colocalization with SEC31A partially decreased in LLOMe-treated conditions, while most SEC31A spots retained some TFG signal, indicating that lysosomal TFG is at least in part derived from ERES (Extended Data Fig. 6k–p).

**Extended Data Fig. 7:**
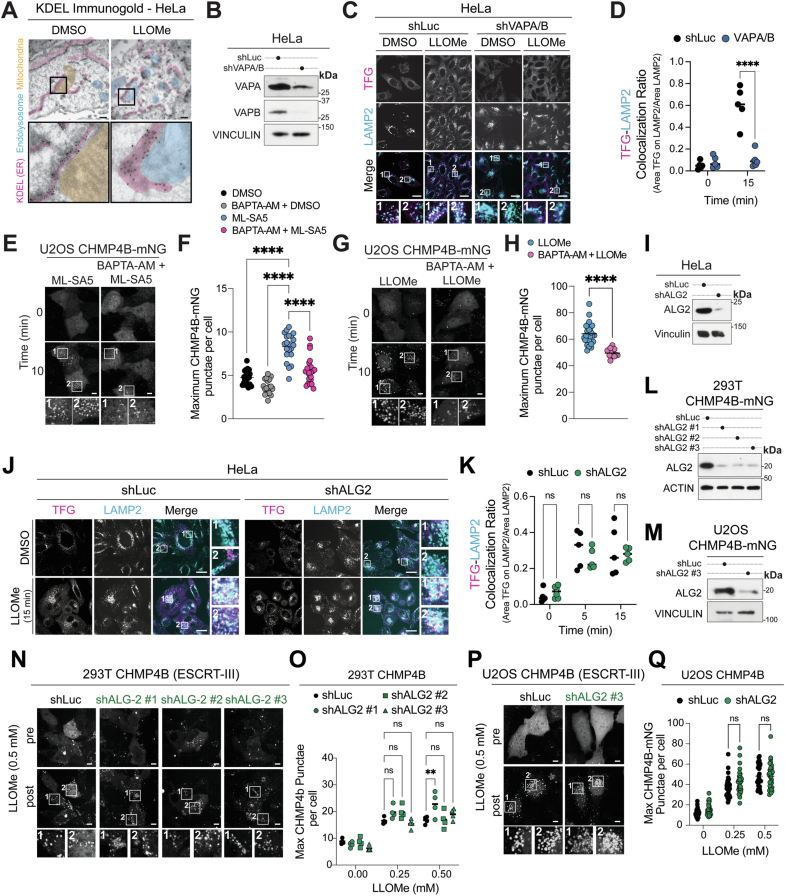
ER-lysosome contacts and calcium release during lysosome damage facilitate TFG translocation to lysosomes. (**A**) KDEL Immunogold-EM of DMSO or 0.5 mM LLOMe treated HeLa cells. Images are shown for 15 min of treatment. Scale bar, 200 nm. Experiment was repeated 2 times. (**B**) Western blot analysis of HeLa cells following knockdown with shRNAs targeting Luc or VAPA and VAPB. VAP knockdown experiments in HeLa were repeated 2 times. (**C, D**) Representative images of TFG and LAMP2 immunofluorescence staining (**C**) and quantification (**D**) of the fraction of LAMP2 colocalized with TFG in HeLa cells following treatment with shRNAs targeting Luc or VAPA and VAPB followed by treatment with DMSO (vehicle control) or 0.5 mM LLOMe for 15 min. Each point is one field of view (FOV; n = 5). Scale bars, 10 µm. (**E, F**) Representative images (**E**) and quantification (**F**) of maximum CHMP4B-mNG puncta in U2OS cells upon treatment with DMSO or 4 µM ML-SA5, with or without pre-treatment for 1 h with 20 µM BAPTA-AM. Images are shown for 10 min of treatment. Each point is one FOV (n = 21). Scale bars, 10 µm. (**G, H**) Representative images (**G**) and quantification (**H**) of maximum CHMP4B-mNG puncta in U2OS cells upon treatment with LLOMe, with or without pre-treatment for 1 h with 20 µM BAPTA-AM. Images are shown for 10 min of treatment. Each point is one FOV (n = 24). Scale bars, 10 µm. (**I**) Western blot analysis of HeLa cells following knockdown with shRNAs targeting Luc control or ALG2. ALG2 knockdown experiments in HeLa were done 2 times. (**J, K**) Representative images (**J**) of TFG and LAMP2 immunofluorescence staining and quantification (**K**) of colocalization ratio between TFG and LAMP2 in HeLa cells. Cells were transduced with shRNAs targeting ALG2 or Luc, followed by treatment with vehicle control (DMSO) or 0.5 mM LLOMe for 5 or 15 min. Images are shown for 15 min of treatment. Each point is one FOV (n = 5). Scale bars, 10 µm. (**L, M**) Western blot analysis of CHMP4B-mNG expressing 293 T (**L**) or U2OS (**M**) cells following knockdown with shRNAs targeting Luc control or ALG2. ALG2 knockdown experiments in U2OS and 293 T were done 2 times. (**N-Q**) Representative images (**N, P**) and quantification (**O, Q**) of maximum CHMP4B-mNG puncta formed in 293 T (**N, O**) or U2OS (**P, Q**) cells treated with DMSO (vehicle control), 0.25 mM, or 0.5 mM LLOMe following treatment with shRNA targeting ALG2 (green) compared to Luc control (black). Images are shown for 15 min of treatment. Each point is one FOV (n = 4 for 293 T; n = 15 for U2OS). Scale bars, 10 µm. Comparison of groups carried out by two-way ANOVA (**D, K, O, Q**), one-way ANOVA (**F**) or two-tailed Welch’s unpaired t-test (**H**). ****p < 0.0001, ***p < 0.001, **p < 0.01, ns = not significant (**D:** t = 15 min, p = 2.7 × 10^−7^, **F:** DMSO vs ML-SA5, p < 1 × 10^−15^; BAPTA + DMSO vs ML-SA5, p < 1 × 10^−15^; ML-SA5 vs BAPTA + ML-SA5, p = 2.0×10^−9^; **H:** p = 1.1 × 10^−8^; **O:** 0.5 mM shLuc vs shALG2#1, p = 5.5 × 10^−3^). Source data

EM–IGS showed enlarged lysosomes wrapped by ER positive for the ER retrieval amino acid motif KDEL, consistent with previous reports^22–24^ (Extended Data Fig. 7a). ER–lysosome membrane contact sites are largely mediated by VAPA and VAPB, which have been implicated in lysosomal repair^22,23^. Of note, co-depletion of VAPA and VAPB by shRNA strongly suppressed LLOMe-induced accumulation of TFG on lysosomes (Extended Data Fig. 7b–d). Together, these data point to a model in which lysosomal damage triggers the relocalization of TFG from ERES to lysosomes, and VAP-mediated physical proximity between ER and lysosomes is required for this translocation to occur.

## TFG binds to ATG8 proteins to form LASER

We next dissected the mechanism that specifically recruits TFG to damaged lysosomes. Release of luminal calcium is an early event during lysosomal membrane damage^20^. The calcium chelator BAPTA-AM blocked both TFG translocation and CHMP4B–mNG accumulation on damaged lysosomes, when co-administered with LLOMe (Fig. 2f,g and Extended Data Fig. 7e–h). Conversely, inducing lysosomal calcium release with ML-SA5, a chemical activator of the lysosomal calcium channel transient receptor potential mucolipin 1 (TRPML1), was sufficient to induce lysosomal accumulation of TFG and CHMP4B–mNG in the absence of LLOMe and in a BAPTA-AM sensitive manner (Fig. 2f,g and Extended Data Fig. 7e–h). We therefore interrogated calcium-dependent processes implicated in lysosomal repair.

ALG2, a calcium-binding protein associated with ERES, has been implicated in several membrane repair processes^14^. However, shRNA-mediated depletion of ALG2 had no effect on TFG translocation from ERES to damaged lysosomes (Extended Data Fig. 7i–k) or on CHMP4B–mNG accumulation (Extended Data Fig. 7l–q).

**Extended Data Fig. 8:**
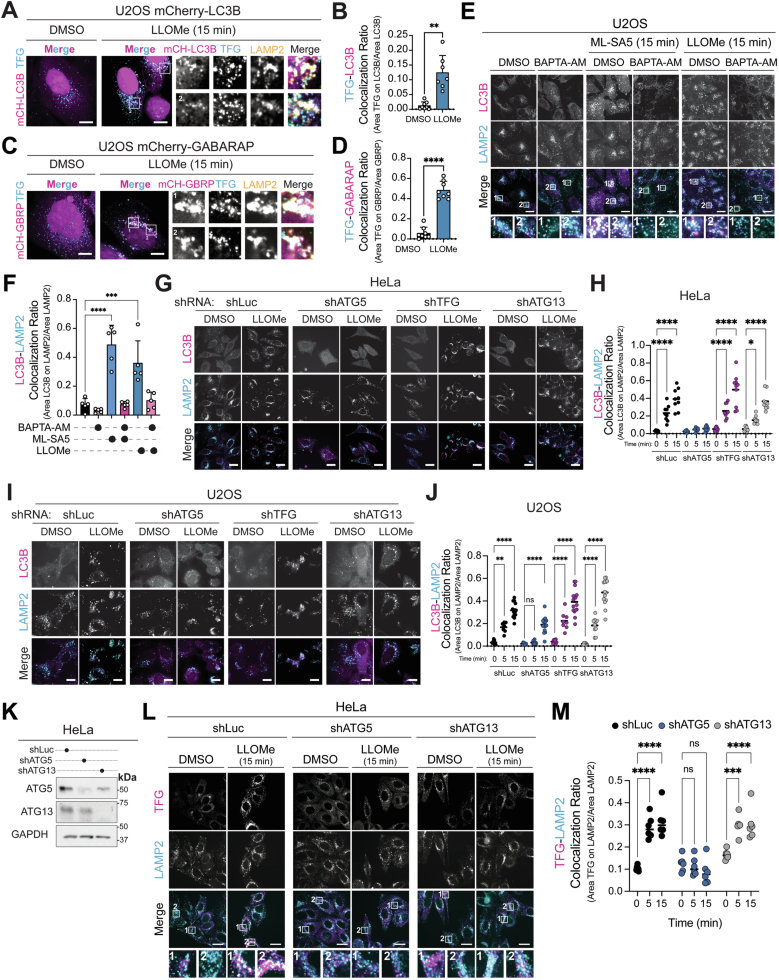
LLOMe treatment or TRPML1-mediated calcium release result in ATG-8ylation of lysosomes. (**A-D**) Representative images (**A, C**) of TFG, mCherry-LC3B/GABARAP, and LAMP2 immunofluorescence staining and quantification (**B, D**) of colocalization ratio between TFG and mCherry-LC3B (**A, B**) or mCherry-GABARAP (**C, D**) in U2OS cells expressing mCherry-LC3B (**A, B**) or mCherry-GABARAP (**C, D**). Cells were treated with DMSO or 0.5 mM LLOMe for 15 min. Each point is one FOV (n = 7 for **B** and n = 8 for **D**). Scale bars, 5 µm. (**E, F**) Representative images (**E**) of LC3B and LAMP2 immunofluorescence staining and quantification (**F**) of colocalization ratio between LC3B and LAMP2 in U2OS cells. Cells were treated with vehicle control (DMSO) or 20 µM BAPTA-AM, followed by treatment with either 4 µM MLSA5 or 0.5 mM LLOMe for 15 min. Each point is one FOV (n = 5). Scale bars, 10 µm. (**G-J**) Representative images (**G, I**) of LC3B and LAMP2 immunofluorescence staining and quantification (**H, J**) of colocalization ratio between LC3B and LAMP2 in HeLa (**G, H**) or U2OS (**I, J**) cells. Cells were treated with shRNAs against ATG5, ATG13, TFG, or Luc control, followed by treatment vehicle control (DMSO) or 0.5 mM LLOMe. Each point is one field of view (FOV; n = 9). Images are shown for 15 min of treatment. Scale bars, 10 µm. (**K**) Western blot analysis of HeLa cells following knockdown with shRNAs targeting Luc control, ATG5, or ATG13. HeLa ATG5 and ATG13 knockdown experiments were repeated 2 times. (**L, M**) Representative images (**L**) of TFG and LAMP2 immunofluorescence staining and quantification (**M**) of colocalization ratio between TFG and LAMP2 in HeLa cells. Cells were transduced with shRNAs targeting ATG5, ATG13, or Luc, followed by treatment with DMSO (vehicle control) or 0.5 mM LLOMe for 5 or 15 min. Each point is one FOV (n = 6). Images are shown for 15 min of treatment. Scale bars, 10 µm. Comparison of groups carried out by two-sided Welch’s unpaired t-test (**B, D**) or one-way ANOVA (**F, H, J, M**). ****p < 0.0001, ***p < 0.001, *p < 0.05, ns=not significant. Error bars represent mean +/− standard deviation (B, D, F) (**B:** p = 1.9 × 10^−3^; **D:** p = 1.1 × 10^−7^; **F:** Untreated vs ML-SA5, p = 6.1 × 10^−7^; Untreated vs LLOMe, p = 1.4 × 10^−4^; **H:** shLuc 0 min vs shLuc 5 min, p = 4.4 × 10^−6^; shLuc 0 min vs shLuc 15 min, p = 8 × 10^−15^; shTFG 0 min vs shTFG 5 min, p = 3.6 × 10^−6^; shTFG 0 min vs shTFG 15 min, p < 1 × 10^−15^; shATG13 0 min vs shATG13 5 min, p = 4.5 × 10^−2^, shATG13 0 min vs shATG13 15 min, p = 3.4 × 10^−12^; **J:** shLuc 0 min vs shLuc 5 min, p = 1.6 × 10^−3^; shLuc 0 min vs shLuc 15 min, p < 1 × 10^−15^; shATG5 0 min vs shATG5 15 min, p = 1.9 × 10^−7^; shTFG 0 min vs shTFG 5 min, p = 1.3 × 10^−6^; shTFG 0 min vs shTFG 15 min, p < 1 × 10^−15^; shATG13 0 min vs shATG13 5 min, p = 2.0 × 10^−5^, shATG13 0 min vs shATG13 15 min, p < 1 × 10^−15^; **M:** shLuc 0 min vs shLuc 5 min, p = 2.4 × 10^−7^; shLuc 0 min vs shLuc 15 min, p = 9.9 × 10^−9^; shATG13 0 min vs shATG13 5 min, p = 1.2 × 10^−4^; shATG13 0 min vs shATG13 15 min, p = 7.0 × 10^−5^). Source data

CASM is a lysosomal repair process that is linked, at least in part, to calcium efflux^15,19,20^. Consistent with CASM induction, LLOMe treatment led to accumulation of endogenous LC3B, mCherry-tagged LC3B and GABARAP on LAMP2-positive lysosomes that also contained TFG (Extended Data Fig. 8a–f). In line with its calcium dependence, lysosomal LC3B conjugation was inhibited by BAPTA-AM and was induced by the TRPML1 agonist ML-SA5 in the absence of LLOMe (Extended Data Fig. 8e,f).

**Fig. 3:**
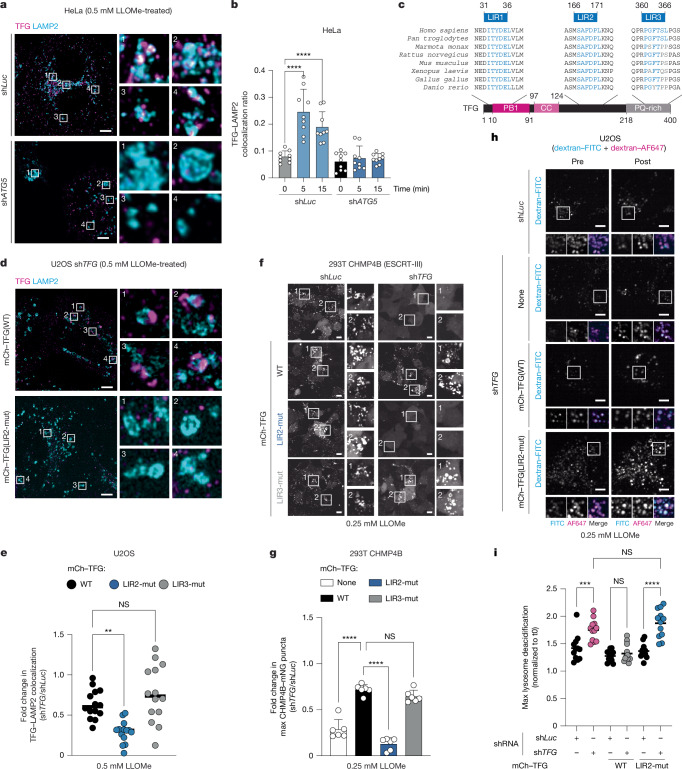
TFG binds to the ATG8 proteins to form LASER on damaged lysosomal membranes. **a**,**b**, Representative images (**a**; *t* = 15 min) and quantification (**b**) of TFG–LAMP2 colocalization in HeLa cells treated with sh*ATG5* or sh*Luc* and 0.5 mM LLOMe. Each point represents one FOV (*n* = 9). Scale bars, 5 µm. **b**, sh*Luc* 0 versus 5 min: *P* = 2 × 10^−6^; sh*Luc* 0 versus 10 min: *P* = 8.3 × 10^−5^. **c**, Top, multiple sequence alignment of TFG LIRs (conserved, blue; non-conserved, grey). Bottom, schematic of TFG, with oligomerization domains (Phox and Bem1 (PB1) and CC) in pink and PQ-rich domains in grey. **d**,**e**, Representative images (**d**; *t* = 20 min) and quantification (**e**) of TFG–LAMP2 colocalization in U2OS cells treated with 0.5 mM LLOMe and sh*TFG* or sh*Luc*, reconstituted with wild-type (WT) or LIR2-mutant (LIR2-mut; SAFDPL>SAADPA) TFG. Each point represents one FOV (*n* = 14). Scale bars, 5 µm. **e**, Wild type versus LIR2-mut, *P* = 1 × 10^−10^. **f**,**g**, Representative images (**f**; *t* = 20 min) and quantification of fold change in maximum number (**g**) of CHMP4B–mNG puncta per cell across the time course in 293T cells treated with 0.25 mM LLOMe and sh*Luc* or sh*TFG*, reconstituted with wild-type, LIR2-mut or LIR3-mut (PGFTSL>PGATSA) TFG. Each point represents one FOV (*n* = 6). Scale bars, 10 µm. **g**, None versus WT: *P* = 2.24 × 10^−8^; WT versus LIR2: *P* = 1.0 × 10^−10^. **h**,**i**, Representative images (**h**) and quantification (**i**) of fluorescence intensity (FITC- versus Alexa Fluor 647-labelled dextran) in U2OS cells treated with 0.25 mM LLOMe and sh*Luc* or sh*TFG*, reconstituted with wild-type or LIR2-mutant TFG. Each point represents one FOV (*n* = 12). Scale bars, 10 µm. **i**, sh*Luc* versus sh*TFG*: *P* = 2.8 × 10^−4^, sh*Luc* versus sh*TFG* plus LIR2: *P* = 7.0 × 10^−7^. Comparison of groups by one-way ANOVA (**b**,**e**,**g**,**i**). Data are mean ± s.d. Source data

**Extended Data Fig. 9:**
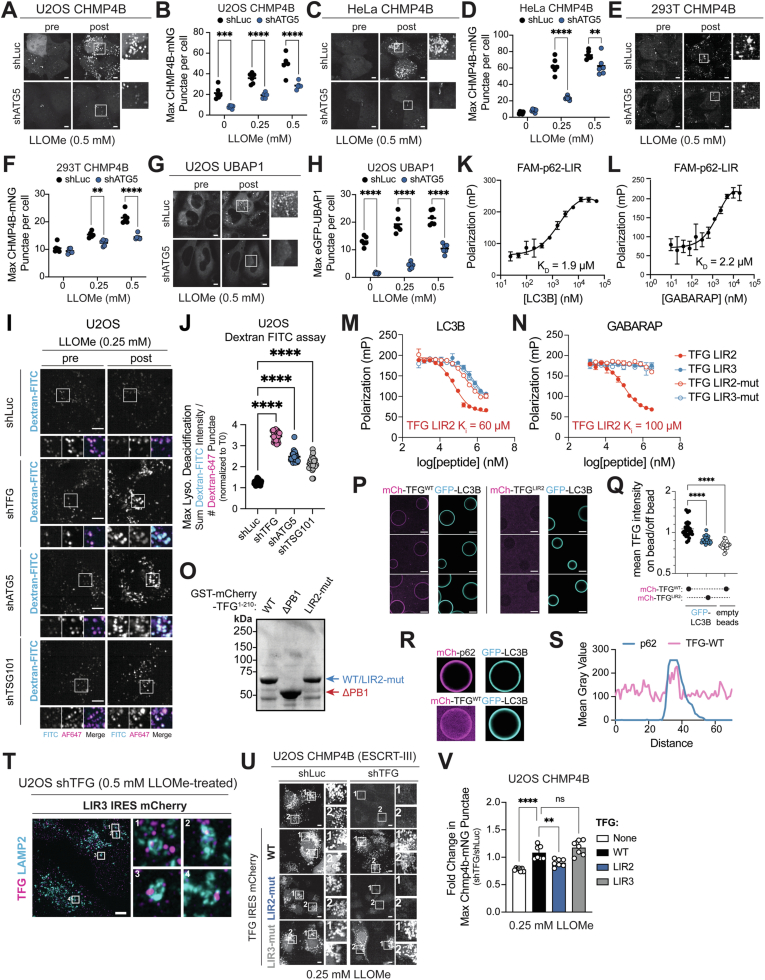
CASM and the LIR2-dependent recruitment of TFG to damaged lysosomes is required for ESCRT recruitment. (**A-F**) Representative images (**A, C, E**) and quantification (**B, D, F**) of fold-change in maximum CHMP4B-mNG puncta per cell across the time course in U2OS (**A, B**), HeLa (**C, D**) or 293 T (**E, F**) cells following treatment with vehicle control (DMSO), 0.25 mM, or 0.5 mM LLOMe and knockdown with shRNAs against Luc or ATG5. Each point is one FOV (n = 6). Images are shown for 15 min of treatment. (**G, H**) Representative images (**G**) and quantification (**H**) of fold-change in maximum eGFP-UBAP1 puncta per cell across the time course in U2OS cells upon treatment with vehicle control (DMSO), 0.25 mM, or 0.5 mM LLOMe and knockdown with shRNAs against Luc or ATG5. Each point is one FOV (H: n = 6). Images are shown for 40 min of treatment. (**I, J**) Representative images (**I**) and quantification (**J**) of total fluorescence intensity ratio between internalized dextran (FITC vs AlexaFluor−647) punctae following treatment with 0.25 mM LLOMe. U2OS cells were transduced with shRNAs targeting Luc, TFG, ATG5, or TSG101. Each point is one FOV (n = 24). (**K, L**) Binding curve, measured by fluorescence polarization of FAM-p62-LIR peptide incubated with increasing concentrations of recombinant human LC3B (**K**) or GABARAP (**L**) protein. Quantification of n = 3 replicates. (**M, N**) Peptide competition, measured by fluorescence polarization, in which FAM-p62-LIR peptide was pre-incubated with recombinant human LC3B (**M**) or GABARAP (**N**), followed by addition of increasing concentrations of unlabeled TFG peptides (LIR2, LIR2-mutant, LIR3, LIR3 mutant). Quantification of n = 3 replicates (**O**) Coomassie staining of purified recombinant 6xHis-mCherry-TFG^1-210^ WT versus mutant (ΔPB1 and LIR2 mutant) proteins. TFG purifications were done at least 3 times and experiments with recombinant TFG were repeated at least 2 times. (**P, Q**) Representative images (**P**) and quantification (**Q**) of binding between recombinant mCherry-TFG^1-210^ WT (***left***) or LIR2-mutant (SAFDPL > SAADPA) (***right***) and GFP-trap beads coated with Flag-GFP-LC3B. Quantification of 16–30 individual beads. (**R, S**) Representative images (**R**) and line graph quantification (**S**) of bead-bound mCherry-p62 (***top***) versus mCherry-TFG^1-210^ WT (***bottom***) recombinant proteins. GFP-trap beads were coated with Flag-GFP-LC3B. (**T**) Representative images of TFG and LAMP immunofluorescence staining in 0.5 mM LLOMe treated U2OS cells following treatment with shRNA against TFG and reconstitution with LIR3-mutant (PGFTSL > PGATSA) TFG protein (related to Fig. 3d). Images are shown for 15 min of treatment. Scale bars, 5 µm. (**U, V**) Representative images (**U**) and quantification (**V**) of fold-change in maximum CHMP4B-mNG per cell across the time course upon treatment with 0.25 mM LLOMe in U2OS cells reconstituted with either WT, LIR2-mutant (SAFDPL > SAADPA), or LIR3-mutant (PGFTSL > PGATSA) TFG and treated with shRNAs against endogenous TFG or Luc control. Each point is one FOV (n = 7). Images are shown for 30 min of treatment. Comparison of groups carried out by two-way ANOVA (**B, D, F, H**) or one-way ANOVA (**J, Q, V**). ****p < 0.0001, ***p < 0.001, **p < 0.01, ns=not significant. Scale bars, 10 µm, unless otherwise stated. Error bars represent mean +/− standard deviation (K, L, M, N) (**B:** DMSO, p = 9.8 × 10^−4^; 0.25 mM LLOMe, p = 8.1 × 10^−5^; 0.5 mM LLOMe, p = 5.6 × 10^−6^; **D**: 0.25 mM LLOMe, p = 6.6 × 10^−11^; 0.5 mM LLOMe, p = 4.6 × 10^−3^; **F:** 0.25 mM LLOMe, p = 1.5 × 10^−3^; 0.5 mM LLOMe, p = 1.2 × 10^−9^; **H:** DMSO: p = 1.3 × 10^−10^; 0.25 mM LLOMe: 2.7 × 10^−13^; 0.5 mM LLOMe 3.9 × 10^−10^; **J:** p < 1 × 10^−15^ for all comparisons plotted; **Q:** TFG^WT^ vs TFG^ΔPB1^ p = 1.4 × 10^−5^; TFG^WT^ vs TFG^WT^ + empty bead, p = 1.4 × 10^−12^; **V:** None vs WT, p = 1.8 × 10^−5^; WT vs LIR2, p = 6.0 × 10^−3^). Source data

Lysosomal LC3B conjugation was abolished by shRNA-mediated knockdown of the obligate E3-like component ATG5 but not of ATG13, which is required for canonical macroautophagy but dispensable for CASM^15^ (Extended Data Fig. 8g–j). Notably, TFG was not required for LLOMe-induced lysosomal LC3B conjugation (Extended Data Fig. 8g–j). We then tested whether, conversely, CASM could be responsible for the recruitment of TFG to damaged lysosomes. Consistent with this possibility, depletion of ATG5, but not ATG13, abolished LLOMe-induced relocalization of TFG to LAMP2-positive lysosomes (Fig. 3a,b and Extended Data Fig. 8k–m). Moreover, in line with the requirement for TFG in ESCRT assembly and lysosomal repair, depleting ATG5 abolished accumulation of eGFP–UBAP1 and CHMP4B–mNG on damaged lysosomes (Extended Data Fig. 9a–h) and inhibited repair to a similar degree as depleting TFG or TSG101, as assessed by dextran–fluorescein isothiocyanate (FITC) unquenching (Extended Data Fig. 9i,j).

Inspection of the amino acid sequence of TFG revealed three predicted, highly conserved LC3-interacting regions (LIRs); LIR2 and LIR3 are in exposed, unstructured domains and were therefore selected for testing^37^ (Fig. 3c). Fluorescence polarization experiments demonstrated binding of a peptide encompassing LIR2 of TFG to recombinant LC3B and GABARAP, with apparent dissociation constants of 60 μM and 100 μM, respectively (Extended Data Fig. 9k–n). By contrast, peptides containing a mutated LIR2 (FDPL>ADPA, LIR2-mut) or encompassing LIR3 (or mutant LIR3 (FTSL>ATSA, LIR3-mut)) did not bind to either LC3B or GABARAP (Extended Data Fig. 9m,n).

We next assessed whether TFG and LC3B could directly interact in an equilibrium binding assay. We purified truncated wild-type and LIR2-mutant mCherry–TFG^1–210^ (full length TFG, including the C-terminal intrinsically disordered region, could not be purified owing to aggregation and precipitation), and incubated these with agarose beads coated with Flag–GFP–LC3B. Recombinant wild-type TFG exhibited detectable association with bead-bound LC3B (Extended Data Fig. 9o–q). Identical amounts of the LIR2-mutant TFG^1–210^ did not exhibit any detectable binding to LC3B (Extended Data Fig. 9o–q).

In line with the lower affinity of the TFG-LIR2 peptide versus that of p62-LIR peptide for LC3B (approximately 60 µM for TFG-LIR2 versus 2 µM for p62-LIR) (Extended Data Fig. 9k–n), the binding of wild-type mCherry–TFG^1–210^ to LC3B-containing beads was weaker than binding of the same amount of mCherry–p62 (Extended Data Fig. 9r,s).

To determine whether LIR2 is functionally required for TFG assembly at damaged lysosomes, we reintroduced wild-type (WT), LIR2-mutant and LIR3-mutant TFG (coupled to mCherry via IRES or P2A to monitor expression levels) into U2OS cells depleted of endogenous TFG. As with the endogenous protein, exogenous wild-type TFG localized to lysosomes upon LLOMe challenge (Fig. 3d,e). Underscoring the essential role of the LIR2–ATG8 interaction, TFG(LIR2-mut), but not TFG(LIR3-mut), did not cluster on LAMP2-positive lysosomes upon LLOMe treatment (Fig. 3d,e and Extended Data Fig. 9t).

Consistent with the critical role for LIR2 in the lysosomal translocation of TFG, TFG-depleted HEK293T and U2OS cells reconstituted with TFG(LIR2-mut) did not recruit CHMP4B–mNG to damaged lysosomes, whereas TFG(LIR3-mut) supported CHMP4B–mNG recruitment as efficiently as the wild-type protein (Fig. 3f,g and Extended Data Fig. 9u,v). Furthermore, TFG(LIR2-mut) did not support lysosomal repair to a similar degree as complete TFG depletion, as assessed by dextran–FITC unquenching (Fig. 3h,i). Thus, a LIR2-mediated interaction of TFG with ATG8 proteins promotes ESCRT assembly on damaged lysosomal membranes, leading to their repair.

On the basis of these data, we propose that TFG and lysosome-conjugated ATG8 proteins together form a functional complex, LASER, that is essential for damage-induced ESCRT polymerization.

## TFG–TSG101 binding drives ESCRT assembly

**Fig. 4:**
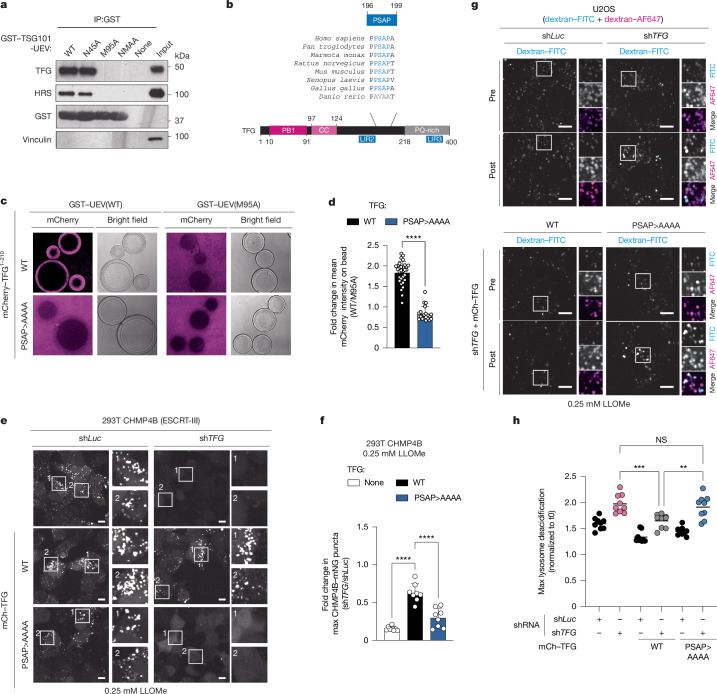
TFG binds the UEV domain of TSG101 to initiate ESCRT assembly on damaged lysosomes. **a**, Western blot analysis of immunoprecipitated recombinant GST fusion with the UEV domain of TSG101 incubated with lysate from 293T cells. TSG101-UEV binding experiments were repeated three times. **b**, Multiple sequence alignment of TFG-PSAP (196–199; conserved, blue; non-conserved, grey). Oligomerization domains are in pink; PQ-rich domain are in grey; solvent-accessible LIRs (LIR2 and LIR3) are in blue. **c**,**d**, Representative images (**c**) and quantification (**d**) of binding of recombinant wild-type or PSAP>AAAA mutant mCherry–TFG^1–210^ to GST beads coated with wild-type or M95A-mutant TSG101-UEV domain. Each point represents one bead. Quantification of *n* = 32 beads (TSG101-UEV(WT)), *n* = 25 beads (TSG101-UEV(M95A)). **d**, Comparison of groups by two-sided unpaired *t*-test; *P* < 1 × 10^−15^. **e**,**f**, Representative images of CHMP4B–mNG (**e**; *t* = 20 min) and quantification of fold change in the maximum number of CHMP4B–mNG puncta per cell (**f**) in 293T cells treated with 0.25 mM LLOMe. Cells were treated with sh*Luc* or sh*TFG* and reconstituted with TFG(WT) or TFG(PSAP>AAAA). Each point represents one FOV (*n* = 8). Scale bars, 10 µm. **f**, One-way ANOVA; none versus WT: *P* = 2.4 × 10^−8^; WT versus PSAP: *P* = 6.5 × 10^−6^. **g**,**h**, Representative images (**g**) and quantification of total fluorescence intensity ratio (**h**) of internalized dextran (FITC- versus Alexa Fluor 647-labelled) puncta following treatment with 0.25 mM LLOMe. U2OS cells were treated with sh*Luc* or sh*TFG* and reconstituted with TFG(WT) or TFG(PSAP>AAAA). Each point represents one FOV (*n* = 9). Scale bars, 10 µm. **h**, One-way ANOVA; sh*TFG* versus sh*TFG* + TFG(WT): *P* = 3.2 × 10^−4^; sh*TFG* + TFG(WT) versus sh*TFG* + TFG(PSAP): *P* = 5.3 × 10^−3^. Data are mean ± s.d. Source data

**Extended Data Fig. 10:**
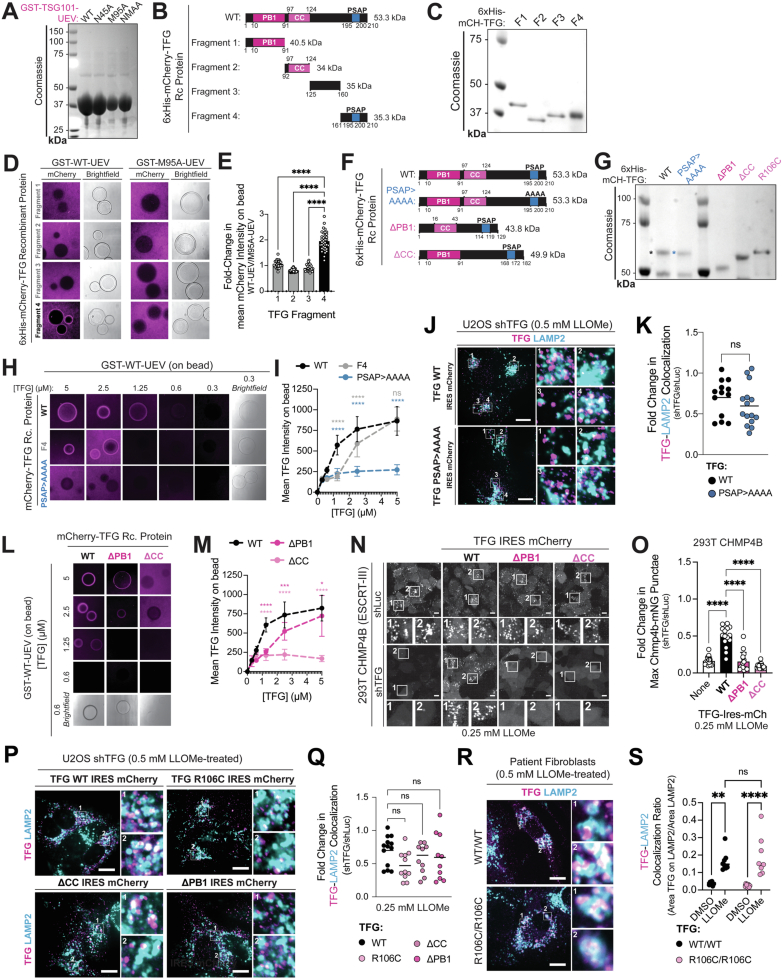
TFG PSAP motif and oligomerization domains are required for ESCRT recruitment. (**A**) Coomassie staining of purified recombinant GST-tagged human TSG101 UEV domains: WT; N45A (Ubiquitin binding mutant); M95A (PSAP binding mutant; NMAA (N45A/M95A double mutant). UEV purifications were done at least 3 times and experiments with recombinant UEV domain were repeated at least 2 times. (**B**) Overview of recombinant 6xHis-mCherry-TFG^1-210^ fragment proteins. (**C**) Coomassie staining of purified recombinant 6xHis-mCherry-TFG^1-210^ fragments. TFG^1-210^ purifications were done at least 2 times and experiments with recombinant TFG fragments were repeated at least 2 times. (**D, E**) Representative confocal images (**D**) and quantification (**E**) of recombinant mCherry-TFG^1-210^ fragments, binding to either wild-type (WT; *left*) or M95A-mutant (*right*) TSG101-UEV domain coated GST beads. Each point represents one bead. Each point represents one individual bead (F1: n = 25; F2: n = 42; F3: n = 23; F4: n = 36). (**F**) Overview of recombinant 6xHis-mCherry-TFG^1-210^ WT versus mutant (PSAP > AAAA, ΔPB1, and ΔCC) proteins. (**G**) Coomassie staining of purified recombinant 6xHis-mCherry-TFG^1-210^ WT and mutant proteins. TFG^1-210^ mutant purifications were done at least 2 times and experiments with recombinant TFG mutants were repeated at least 2 times. (**H, I**) Representative confocal images (**H**) and quantification (**I**) of binding between recombinant mCherry-TFG^1-210^, WT or mutants (F4 or PSAP > AAAA), and GST beads coated with the TSG101-UEV domain. Each point represents one individual bead (in decreasing order of concentration, WT: n = 5, 9, 6, 9, 12, 3; F4: n = 10, 12, 17, 25, 13, 3; PSAP: n = 11, 14, 5, 9, 15, 3). (**J, K**) Representative confocal images (**J**) of TFG and LAMP2 immunofluorescence staining and quantification (**K**) of colocalization ratio between TFG and LAMP2 in U2OS cells. Cells were transduced with shRNAs targeting Luc control or endogenous TFG and reconstituted WT or PSAP > AAAA mutant TFG. Cells were treated with vehicle control (DMSO) or 0.5 mM LLOMe. Each point represents one field of view (FOV). Quantification of n = 14 FOVs. Images are shown for 15 min of treatment. Scale bars, 5 µm. (**L, M**) Representative images (**L**) and quantification (**M**) of binding between recombinant mCherry-TFG^1-210^, WT or mutants lacking the PB1 (ΔPB1) or CC (ΔCC) domains and GST beads coated with the TSG101-UEV domain. Quantification of 8–20 individual beads. (**N, O**) Representative images (**N**) and quantification (**O**) of fold-change in maximum CHMP4B-mNG puncta in 293 T cells upon treatment with vehicle control (DMSO) or 0.25 mM LLOMe and following knockdown with shRNAs against Luc or TFG and reconstituted with wild-type TFG or TFG lacking the PB1 or coiled-coil (CC) domains. Each point is one field of view (FOV; n = 15). Images are shown for 20 min of treatment. Scale bars, 10 µm. (**P, Q**) Representative images (**P**) of TFG and LAMP2 immunofluorescence staining and quantification (**Q**) of colocalization ratio between TFG and LAMP2 in U2OS cells transduced with hairpins targeting Luc control or TFG reconstituted with wild-type TFG, R106C mutant TFG, or TFG lacking the PB1 or coiled-coil (CC) domains. Cells were treated with 0.5 mM LLOMe for 15 min. Each point is one FOV (n = 10). Scale bars, 5 µm. (**R, S**) Representative images (**R**) of TFG and LAMP2 immunofluorescence staining and quantification (**S**) of colocalization ratio between TFG and LAMP2 in TFG^R106C/R106C^ or control (TFG^WT/WT^) patient fibroblast cells treated with 0.5 mM LLOMe for 15 min. Each point is one FOV (n = 8). Scale bars, 5 µm. Comparison of groups carried out by one-way (**E, O, Q**) or two-way (**I, M, S**) ANOVA, or two-sided unpaired t-test (**K**). Error bars represent mean +/− standard deviation. ****p < 0.0001, **p < 0.01, ns = not significant (**E:** p = 2.2 × 10^−14^ for all comparisons; **I:** 5 µM TFG: WT vs PSAP p < 1 × 10^−15^; 2.5 µM TFG: WT vs PSAP p < 1 × 10^−15^, WT vs F4 p = 1.6 × 10^−5^; 1.25 µM TFG: WT vs PSAP p = 2.3 × 10^−10^, WT vs F4 p < 1 × 10^−15^; **M:** 5 µM TFG WT vs ΔPB1, p = 4.0 × 10^−2^, TFG WT vs ΔCC, p = 8.3 × 10^−14^; 5 µM TFG WT vs ΔPB1, p = 2.4 × 10^−4^, TFG WT vs ΔCC, p = 8.8 × 10^−14^; 2.5 µM WT vs ΔPB1, p = 1.3 × 10^−12^, WT vs ΔCC p = 1.1 × 10^−13^; **O:** WT vs None, p = 2.4 × 10^−13^; WT vs ΔCC, p = 8 × 10^−15^; WT vs ΔPB1, p = 1.1 × 10^−13^; **S:** WT/WT DMSO vs WT/WT LLOMe: p = 2.6 × 10^−3^**;** R106C/R106C DMSO vs R106C/R106C LLOMe, p = 8.5 × 10^−5^). Source data

We next sought to determine the molecular mechanism through which LASER promotes ESCRT polymerization on the lysosomal membrane. On the basis of previous literature, we hypothesized that the ubiquitin E2 variant (UEV) domain of TSG101, which is known to bind to both ubiquitin and Pro-Ser/Thr-Ala-Pro (PS/TAP) motifs, may interact with TFG^38^. We expressed and purified recombinant glutathione *S*-transferase (GST)-tagged UEV domains (wild-type, PSAP-binding and ubiquitin-binding mutants), incubated them with cell lysates, and evaluated binding to endogenous TFG (Extended Data Fig. 10a). Wild-type and ubiquitin-binding mutant (N45A) UEV domains bound to TFG, whereas the PSAP-binding mutant (M95A) did not (Fig. 4a). This pattern mirrored the binding between the UEV domain and the ESCRT-0 protein HRS^11^, indicating a putative PSAP-dependent interaction (Fig. 4a).

By inspecting the primary amino acid sequence of TFG, we noted the presence of a highly conserved PSAP motif following the coiled-coil (CC) region (Fig. 4b). Consistent with this motif being the primary UEV-interacting region, when we incubated mCherry-tagged fragments encompassing the N-terminal region of TFG with recombinant UEV immobilized on agarose beads, only the PSAP-containing region (TFG^161–210^, or fragment 4) bound to the beads (Extended Data Fig. 10b–e). Moreover, unlike wild-type recombinant TFG^1–210^, TFG^1–210^ in which the PSAP motif was mutated to AAAA did not bind to the UEV domain (Fig. 4c,d and Extended Data Fig. 10f–i). Consistent with a critical role for the interaction between TSG101-UEV and TFG-PSAP in ESCRT recruitment, the PSAP>AAAA mutant of TFG did not support CHMP4B–mNG clustering on damaged lysosomes in HEK293T cells (Fig. 4e,f), despite its unaltered recruitment to damaged lysosomes, as predicted by an intact LIR2 (Extended Data Fig. 10j,k). In agreement with decreased ESCRT recruitment, TFG-depleted U2OS cells reconstituted with the PSAP>AAAA mutant displayed a comparable repair defect to non-rescued, TFG-depleted cells, as shown by dextran–FITC unquenching (Fig. 4g,h).

Thus LASER recruits ESCRT-I, at a minimum, through a specific interaction between the PSAP motif of TFG and the UEV domain of TSG101.

## TFG oligomerization enables ESCRT recruitment

Within the ESCRT pathway, oligomerization-driven cooperativity enables efficient assembly, despite low-affinity interactions among the various ESCRT components^10,11^. TFG is also thought to exist in a highly assembled state. It undergoes oligomerization via its N-terminal PB1 and CC domains, whereas a PQ-rich, low-complexity region encompassing the C-terminal half of TFG drives its liquid–liquid phase separation (LLPS)^39–41^.

Of note, TFG^161–210^, which lacks all oligomerization domains, displayed substantially reduced binding compared with wild-type TFG^1–210^, despite having an intact PSAP motif (Extended Data Fig. 10h,i). This finding was consistent with previous reports that the PSAP motif of HRS binds weakly to the UEV of TSG101 and requires oligomerization-driven avidity for stable association. We thus hypothesized that TFG oligomerization, mediated by its PB1 and CC domains^40^, enhances TSG101 binding through avidity-based stabilization. Consistent with this possibility, deleting the CC domain from the recombinant TFG^1–210^ completely abolished interaction with recombinant UEV, whereas deleting the PB1 domain significantly weakened it, despite the presence of an intact PSAP in both constructs (Extended Data Fig. 10g,l,m).

Consistent with the reduced in vitro binding, in TFG-depleted HEK293T cells reconstituted with exogenous TFG constructs, both PB1-deleted and CC-deleted TFG failed to support lysosomal CHMP4B–mNG recruitment upon LLOMe treatment (Extended Data Fig. 10n,o), despite localizing correctly to lysosomes (Extended Data Fig. 10p,q). Thus, TFG oligomerization mediated by the PB1 and CC domains enhances its PSAP-dependent interaction with TSG101, supporting a model in which oligomeric TFG within LASER accelerates ESCRT assembly on damaged lysosomes.

**Fig. 5:**
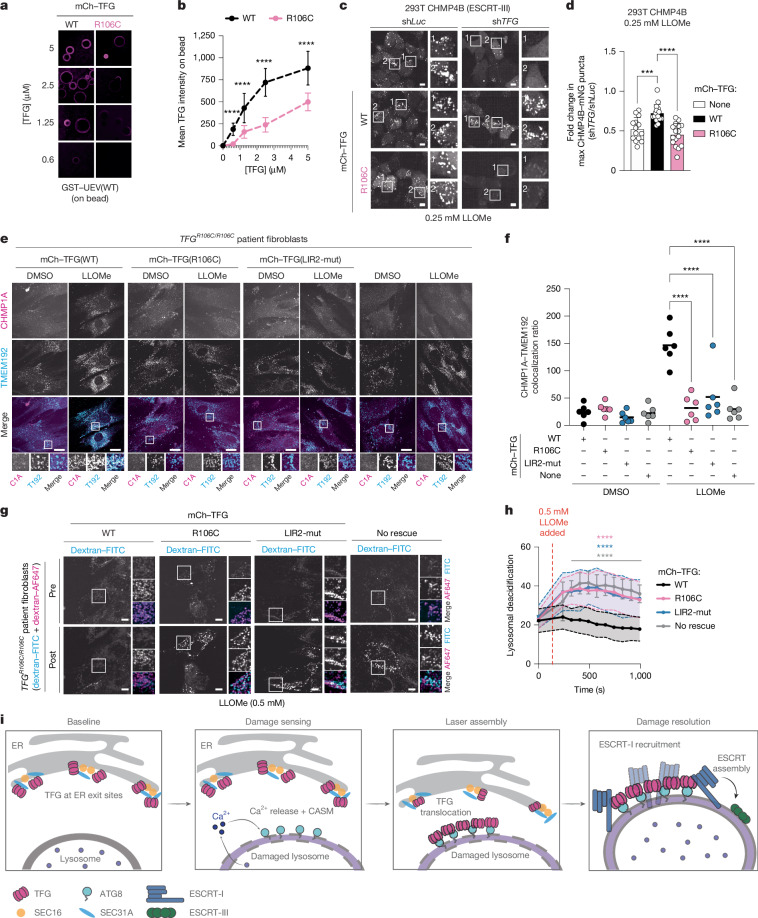
TFG(R106C) impairs ESCRT recruitment and sensitizes cells to lysosome damage. **a**,**b**, Representative images (**a**) and quantification (**b**) of binding between mCherry–TFG^1–210^ (wild type or R106C mutant) and GST–TSG101-UEV. Each point represents one bead (WT: *n* = 65 (0.6 µM), *n* = 77 (1.25 µM), *n* = 88 (2.5 µM), *n* = 29 (5 µM); R106C: *n* = 25 (0.6 µM), *n* = 44 (1.25 µM), *n* = 61 (2.5 µM), *n* = 40 (5 µM)). 5 µM, 2.5 µM, 1.25 µM: *P* < 1 × 10^−15^; 0.6 µM: *P* = 1.1 × 10^−7^. **c**,**d**, Representative images (**c**; *t* = 20 min) and quantification of fold change in the maximum number (**d**) of CHMP4B–mNG puncta across the time course. 293T cells expressing shRNA-resistant TFG(WT) or TFG(R106C) were treated with sh*TFG* or sh*Luc*, followed by treatment with 0.25 mM LLOMe. Each point represents one FOV (*n* = 14). **d**, None versus WT: *P* = 4.8 × 10^−4^; WT versus R106C: *P* = 2.5 × 10^−6^. **e**,**f**, Representative immunofluorescence images (**e**; *t* = 7 min) and quantification (**f**) of CHMP1A–TMEM192 colocalization in fibroblasts from individuals with HSP (*TFG*^*R106C/R106C*^) reconstituted with wild-type, R106C or LIR2-mutant (SAFDPL>SAADPA) TFG and treated with 0.5 mM LLOMe. Each point represents one FOV (*n* = 6). **f**, WT versus R106C: *P* = 2.3 × 10^−8^; WT versus LIR2-mut: *P* = 2.0 × 10^−6^; WT versus none: *P* = 1.5 × 10^−8^; **g**,**h**, Representative images (**g**) and quantification (**h**) of FITC versus Alexa Fluor 647 dextran fluorescence intensity following treatment with 0.5 mM LLOMe. Fibroblasts from individuals with HSP (*TFG*^*R106C/R106C*^) were reconstituted with wild-type, R106C or LIR2-mutant TFG. Each point represents one FOV (*n* = 11). **h**, *P* < 1 × 10^−15^ for all comparisons. **i**, Model of LASER-dependent ESCRT recruitment. Lysosomal damage triggers calcium leakage, CASM and ER–lysosome contacts. TFG dissociates from ERES and binds ATG8 proteins, forming LASER. TFG directly binds TSG101 to initiate ESCRT-mediated repair. Comparison of groups by two-way (**b**,**h**) or one-way (**d**,**f**) ANOVA. Data are mean ± s.d. Scale bars, 10 µm. Source data

The pathogenic R106C mutation—located in the CC domain of TFG—has been linked to autosomal recessive, early-onset HSP^34,40^. In vitro, this mutation distorts the ring-like structures formed by the PB1 and CC portions of TFG^34^. Consistent with a critical role for oligomerization, recombinant TFG(R106C) exhibited strongly reduced binding to the UEV domain of TSG101 (Fig. 5a,b and Extended Data Fig. 10g). Despite localizing correctly to lysosomes in LLOMe-treated conditions, and as predicted by the TSG101-binding defect, TFG(R106C) did not support lysosomal CHMP4B–mNG recruitment in knockdown–reconstitution experiments (Fig. 5c,d and Extended Data Fig. 10p,q).

We further dissected the role of the CC domain in skin fibroblasts from individuals with HSP who are homozygous for the R106C mutation. In agreement with the knockdown–reconstitution experiments in HEK293T cells, *TFG*^*R106C/R106C*^ fibroblasts exhibited defective LLOMe-dependent recruitment of endogenous CHMP1A to damaged lysosomes, which was rescued by re-expression of wild-type TFG but not of TFG(R106C) or TFG(LIR2-mut) (Fig. 5e,f). Notably, the repair defect occurred despite unchanged recruitment of TFG(R106C) to damaged lysosomes relative to wild-type TFG (Extended Data Fig. 10r,s). Defective ESCRT recruitment was paralleled by impaired ability of TFG(R106C) to support lysosomal repair, as shown by dextran–FITC unquenching (Fig. 5g,h).

Together, these data support the possibility that defective TFG oligomerization within LASER compromises its ability to recruit ESCRT and maintain lysosomal integrity and may underlie, at least in part, the neuropathological effects of the R106C mutation in TFG.

## Discussion

How detection of lysosomal membrane damage is coupled to its ESCRT-mediated repair has not been fully elucidated. Here we define LASER as a rapid, early-acting molecular assembly that bridges damage sensing to ESCRT-I activation, thereby resolving a central mechanistic gap in lysosomal membrane repair. Our results support a model in which ATG8 proteins, conjugated to lysosomal membrane phospholipids upon onset of damage and luminal calcium release, act as a physical anchor for oligomeric TFG. Within LASER, TFG binds to ATG8 proteins via LIR2, leading to its association with damaged lysosomes (Fig. 5i). Oligomeric TFG at sites of lysosomal membrane damage acts as a hub that helps to recruit TSG101 via combined affinity (PSAP-dependent) and avidity (CC-dependent) effects, thus accelerating the downstream assembly of ESCRT-II and ESCRT-III (Fig. 5i). In this way, LASER converts local damage signals into cooperative ESCRT polymerization, ensuring spatially restricted membrane repair.

It was previously shown that, upon CASM induction, binding between GABARAPL2 and ALIX promotes recruitment of ALIX to damaged lysosomes, thus leading to ESCRT assembly^18^. However, our data and other reports point to ALIX having a conditional, non-essential role in ESCRT recruitment to damaged lysosomes^7,8^. A recent report supports the strict requirement for ATG5–ATG12 in the recruitment of ESCRT subunits, while hinting that this complex may have additional roles, independent of ATG8 conjugation, in promoting ESCRT assembly^15^. However, the biochemical mechanisms through which CASM recruits ESCRT-I, and particularly TSG101, to damaged lysosomes were not established. Our data identify a crucial consequence of CASM induction: the recruitment of ERES-derived, oligomeric TFG to damaged lysosomes, forming LASER as a dedicated platform for TSG101 recruitment to trigger ESCRT assembly. As CASM supports multiple lysosomal repair and quality control pathways, including lipid transfer and membrane remodelling^15,18,42^, an important follow-up question will be how LASER is coordinated with other CASM-dependent repair pathways.

Lysosomal membrane damage occurs along a continuum, ranging from subtle lipid packing defects and ion leakage to progressive membrane ruptures and, eventually, irreversible permeabilization^1,6^. In this study, we primarily relied on LLOMe as a tractable and temporally precise model of lysosomal membrane damage, as LLOMe-derived poly-Leu oligomers recapitulate key biophysical features of progressive lysosomal injury, from induction of lipid packing defects to nanoscale membrane lesions and eventual permeabilization^6^. Our data support a model in which LASER activates ESCRT rapidly upon damage onset. Alternative, TFG-independent mechanisms for ESCRT recruitment (for example, ALG2–ALIX) may become increasingly important at more severe stages of damage^14,15,18^. However, our findings position LASER as a principal early trigger of ESCRT assembly at reversible stages of lysosomal injury.

LASER expands the repertoire of mechanisms that locally activate ESCRT polymerization on membranes. Similar to ubiquitin-dependent ESCRT-0-mediated activation during intraluminal vesicle formation, or CEP55- and LEMD2-driven activation during cytokinesis and nuclear envelope closure, LASER relies on low-affinity interactions amplified by oligomerization^11^. For example, the PSAP motif of HRS has relatively weak affinity for the UEV domain of TSG101, necessitating cooperativity via ESCRT-0 oligomerization^43^. Similarly, CC-dependent oligomerization was essential for TFG to bind and recruit TSG101 to lysosomes. Thus, LASER obeys a unifying principle of ESCRT regulation: spatially confined polymerization driven by cooperative, multivalent interactions that amplify weak motif recognition into membrane remodelling events. In addition to PSAP-mediated interactions, the ESCRT-I subunits TSG101 and UBAP1 bind directly to ubiquitin via their UEV and UBA domains, respectively^38^. It remains to be determined whether any ESCRT subunits engage in PSAP-independent interactions with TFG.

TSG101 can be found in an autoinhibited state mediated by an intramolecular UEV–PTAP interaction, which is displaced by the PSAP motif of HRS, triggering ESCRT-I dependent recruitment of ESCRT-II^11^. It remains to be determined whether, analogous to HRS, oligomeric TFG may help to relieve the autoinhibited state of TSG101, possibly in response to its engagement of ATG8 proteins within LASER. If so, LASER may not only recruit ESCRT-I but actively prime it for polymerization. Finally, the functional partnership between TFG and ATG8 proteins may extend to other membrane remodelling processes involving ESCRT, including LC3-associated phagocytosis^44^, phagophore closure^45^ and microautophagy-dependent ERES degradation^11^.

Although our data establish a requirement for TFG oligomerization via its PB1 and CC domains, whether TFG phase separation contributes to ESCRT recruitment remains unresolved. Phase-separated stress granules have been shown to accumulate rapidly at damaged lysosomes to stabilize the ruptured lysosomal membrane or to trigger specific signalling responses^46,47^. Conversely, phase separation of ESCRT-recruiting proteins has been shown to facilitate nuclear envelope reformation in mammalian cells, as well as MVB formation in plants^11,48^. TFG condensates with a hollow morphology were recently reconstituted and proposed to act as ‘molecular sieves’ that may concentrate COPII coat subunits within their internal space to regulate COPII vesicle formation^41^. In the context of LASER, such higher-order assemblies could further enhance local concentration and cooperativity of ESCRT subunits, reinforcing their rapid and spatially restricted activation at damaged lysosomal membranes.

The calcium-dependent mobilization of TFG and ALG2 from ERES suggests that ERES function as regulated reservoirs of membrane repair factors, which are released upon detection of specific alarm signals. TFG localization at ERES, mediated by interactions with SEC23^35^, may prevent its premature or aberrant activation of ESCRT polymerization, which could have deleterious effects on lysosomal membrane homeostasis. An important aspect of this model is the requirement for physical proximity between ERES and lysosomes. This proximity relies on VAPA and VAPB-dependent ER–lysosome membrane contact sites, which have been extensively shown to become expanded in the context of lysosomal membrane damage^22–24^, and may be critical for sensing of lysosome-derived calcium at ERES.

The requirement for VAP proteins may also reflect coordination between LASER and repair-promoting mechanisms for lipid exchange at ER–lysosome contacts^22–24^. ER–lysosome contacts enable directional lipid transfer by ER-bound OSBP-related proteins and VPS13, which are anchored to the lysosomal membrane by PI4P and RAB7, respectively^22–24^. The physical proximity between ER and damaged lysosomes may enable TFG to recruit ESCRT in close spatial proximity to the sites of phospholipid delivery, a mechanism reminiscent of the cooperation between ESCRT and VPS13 in MVB formation^49^. Thus, these findings establish ER–lysosome contacts as regulatory platforms that coordinate lysosomal damage sensing with ESCRT-dependent membrane remodelling and with lipid replenishment.

TFG has a role in spatially organizing ERES, and TFG loss of function has been linked to secretory defects^35,40^. The observation that TFG translocates from ERES to lysosomes upon damage suggests that the roles of TFG in secretory traffic and lysosomal membrane repair are separable. Moreover, our results with *TFG*^*R106C*^ mutant cells suggest that defective maintenance of lysosomal integrity could synergize with secretory defects in driving the neuropathic manifestations of TFG-linked HSP. Additional studies are required to fully elucidate the relationship between secretory and lysosome-repairing functions of TFG in both normal physiology and in the context of HSP.

The identification of LASER as a lysosomal repair-promoting factor was made possible by the use of a damage-sensitized genetic background, NPC1-deleted cells. Our genome-wide screen and follow-up studies suggest that defective lysosomal repair may be a common driver in many neurodegenerative diseases linked to lipid imbalance or defective ESCRT assembly. More broadly, our work establishes lysosomal membrane resealing as a multi-layered process in which damage sensing, membrane contact site remodelling and ESCRT polymerization are molecularly integrated.

## Methods

### Antibodies and chemicals

Reagents were obtained from the following sources: antibodies against HA (3724S, clone C29F4; 1:1,000 dilution for western blot), LC3B (3868S, clone D11; 1:1,000 dilution for western blot), VINCULIN (13901S, clone E1E9V; 1:1,000 dilution for western blot), LAMTOR4 (13140S, clone D4P60; 1:300 dilution for immunofluorescence), HRS (15087S, clone D7T5N; 1:1,000 dilution for western blot), TOM20 (42406S, clone D8T4N; 1:1,000 dilution for western blot), ATG5 (2630S; 1:1,000 dilution for western blot), ATG13 (13468S, clone E1Y9V; 1:1,000 dilution for western blot) from Cell Signaling Technology; LAMP2 (sc-18822, clone H4B4; 1:300 dilution for immunofluorescence), CHMP1A (sc-271617, clone B-5; 1:300 dilution for immunofluorescence) from Santa Cruz Biotechnology; TFG (ab156866, clone EPR8766; 1:300 dilution for immunofluorescence, 1:1,000 dilution for western blot) and NPC1 (ab36983; 1:1,000 dilution for western blot) from Abcam; SEC31A (612350, clone 32; 1:300 dilution for immunofluorescence) from BD Biosciences; SEC31A (17913-1-AP;1:300 dilution for immunofluorescence), TSG101 (67381-Ig, clone 2B7G8; 1:1,000 dilution for western blot), ALIX (67715-Ig, clone 1H9D9; 1:1,000 dilution for western blot), PDCD6/ALG2 (12303-1-AP; 1:1,000 dilution for western blot) from Proteintech; TFG (NBP2-62212, clone TFG-03; 1:300 dilution for immunofluorescence) from Novus; LLOMe (16008) from Cayman; DMSO (D2650-100mL) from Sigma-Aldrich; Pierce Protease Inhibitor Tablets (A32965) from Thermo Fisher; DMEM (11965), DMEM-Phenol Red (31053-028), 0.25% Trypsin-EDTA (25300062), 0.05% Trypsin-EDTA (25300054) from Gibco; U1866A (1638) from Tocris Bioscience; Puromycin (Gibco, A1113803) and Blasticidin (Gibco, A1113903).

### CRISPRi cell line generation and validation

K562 sgNT and K562 sg*NPC1* cell lines were generated using Cas9–sgRNA ribonuclear protein transfection (sg*NPC1* sgRNA sequence: GGACGATCCTTGGCTTGGAC) as previously described^26^, and knockout was validated by western blot (Extended Data Fig. 1b). In total, 2 × 10^5^ cells were transduced with Ef1a-dCas9-KRAB-BFP construct lentivirus in cell culture medium supplemented with 1 μl ml^−1^ Polybrene (Millipore Sigma, TR-1003-G). Cells were spinfected at 1,000*g* for 1 h at room temperature and cultured overnight. Viral supernatant was removed next day and replaced with fresh K562 medium. Cells were expanded and then sorted for BFP+ population on a BD FACS Aria and maintained as a pooled cell line.

To validate CRISPRi efficiency, cells were transduced with lentivirus for guides targeting Gal4 (negative control) or CD55 (cell surface marker). Cells were selected with puromycin (Gibco, A1113803) to obtain a pool of guide containing cells. Cells were incubated with Fc block (Biolegend, 422302) in FACS Buffer (0.5% BSA in PBS) and stained for CD55 (Biolegend, 311312). Knockdown efficiency was analysed by flow cytometry on the Thermo Fisher Attune NxT Flow Cytometer and analysed on FlowJo.

### sgRNA guide library plasmid production

The lentiviral expression vector used for CRISPR guide expression is available on Addgene as pLGR1002 (188320). The sgRNA sequences as well as the sgRNA library cloning procedure performed is as described^50,51^. sgRNA library representation was confirmed by next-generation sequencing.

### Guide library lentivirus production and titration

Guide library virus was prepared using the Mega Lentivirus protocol from the Weissman laboratory with modifications (https://weissman.wi.mit.edu/resources/Large_scale_lentivirus_production.pdf). A total of 18 million Lenti-X 293T cells (Takara Bio, 632180) were plated on a 15 cm plate. The next day, transfection mixture was prepared using 125 μl Mirus LT1 transfection reagent (Mirus Bio, MIR2304), 1.5 ml DMEM (Gibco, 11965-092), 18 μg psPax2 (packaging vector), 4 μg MD2G (env expression plasmid, VSVG) and 18 μg of lentiviral plasmid (sgRNA library). Transfection mixture was incubated for 15–20 mins at room temperature. Transfection mix was added to the 293T cells and incubated for 6 h. After 6 h the medium was replaced with fresh 293T medium supplemented with 2 μg ml^−1^ ViralBoost (ALSTEM, VB100). Lentiviral supernatant was collected 48 h after transfection and filtered (0.45 μm). Lentivirus was stored at −80 °C until use.

Lentivirus titration was performed in K562 cells prior to the screen. Multiplicity of infection (MOI) was determined by percent infection shown by BFP signal via flow cytometry. K562 sgNT and K562 sg*NPC1* cells (2 × 10^5^) were transduced with guide library lentivirus in a range of 5–250 μl in K562 medium supplemented with 1 μl ml^−1^ Polybrene (Millipore Sigma, TR-1003-G). Cells were left in virus for 24 h and then changed to regular medium. Forty-eight hours after infection, cells were analysed on the Attune NXT flow cytometer to determine BFP fluorescence and MOI.

Lentivirus for individual guide RNA experiments was produced in 6-well plates with adjusted protocol. Lenti-X 293T cells (0.8 × 10^6^) per well were seeded the day before transfection. Transfection mix consists of 7.5 μl Mirus transfection reagent (MIR 2700), 100 μl DMEM (Gibco, 11965-092), 1.2 μg psPAX2, 0.3 μg MD2G and 1.2 μg guide plasmid. The protocol is as described above.

### Lysosomal integrity survival screen

sgNT K562 dCas9-KRAB and sg*NPC1* K562 dCas9-KRAB cells were infected with genome-wide sgRNA library lentivirus to target 1,000× coverage of all guides at a low MOI to ensure roughly 1 guide per cell. Cells from each cell line (around 550 million) were pelleted and resuspended in pure virus supplemented with 1 μl ml^−1^ Polybrene (Millipore Sigma, TR-1003-G). Cells with virus were plated into 6-well plates and spinfected at 1,000*g* for 1 h. Cells were then supplemented with complete medium and cultured in flasks in shaking incubators at 100*g*. Cells were maintained in shaking conditions throughout the screen. After ~24 h, virus was removed and cells were resuspended in fresh medium. The next day BFP infection percentage was assessed (34% for sgNT, 45% for sg*NPC1*) and selection was started with 1 μg ml^−1^ puromycin (Gibco, A1113803). Cells were selected with 1 μg ml^−1^ puromycin for 6 days until population reached ~80–90% BFP^+^. Cells were grown for an additional three days to reach numbers necessary for the screen.

On day T0 of the screen, samples were split for 850× coverage of the library to each replicate. Two replicates from each cell line were frozen in medium with 10% DMSO as backup. Two replicates from each cell line were pelleted and stored at −80 °C for T0 gDNA samples. Two replicates from each cell line were seeded at 0.25 × 10^6^ cells per ml and treated with 0.5 mM LLOMe hydrochloride (Cayman Chemicals, 16008) or 0.01% DMSO mock treatment for another arm of the screen (not shown) and cultured for 2 days. Drug treatment was then removed from cells and replaced with fresh medium for two days. This cycle of drug treatment and recovery was repeated, after which cells were expanded for 6 days until cell numbers were sufficient to end the screen. Multiple cell pellets representing 850× coverage of the library were stored at −80 °C for each sample for gDNA processing.

### Genomic DNA processing, library preparation and sequencing

Cell pellets from the screen were processed using the NucleoSpin Blood XL Kit (Macherey Nagel, 740950) according to manufacturer’s directions and extracted DNA was quantified with DeNovix Nanodrop.

After DNA extraction, samples were processed for NGS sequencing using the protocol described^50^. DNA samples were amplified by PCR using NEBNext Ultra II Q5 MasterMix (NEB, M0544) with a common reverse primer (CAAGCAGAAGACGGCATACGAGATATGCTGTTTCCAGCTTAGCTCTT) and a unique forward primer per sample containing a 6 bp barcode (AATGATACGGCGACCACCGAGATCTACACGATCGGAAGAGCACACGTCTGAACTCCAGTCACnnnnnnGCACAAAAGGAAACTCACCCT) (sequences listed in Supplementary Table 1). For each sample total gDNA was amplified in multiple PCRs with an input of 10 μg per reaction. PCRs were run with the following cycling conditions: 98 °C for 30 s, followed by 24 cycles of 98 °C for 10 s and 62.6 °C for 75 s, and a final elongation at 65 °C for 5 mins. Reactions were pooled and purified with double-sided SPRI beads (Beckman Coulter, A63882). Samples were quantified on the Agilent TapeStation and pooled to generate a library with equal representation of each sample. Library was sequenced on the Illumina NextSeq 550 using a NextSeq High Output kit (75 cycles) (Illumina, 20024906). A custom sequencing primer (GTGTGTTTTGAGACTATAAGTATCCCTTGGAGAACCACCTTGTTGG) was added to the standard Illumina sequencing primer. Library runs were supplemented with PhiX DNA to increase sequence diversity.

### Computational analysis of screens

Two integrated analysis pipelines for pooled CRISPR screens were used to generate and process sgRNA read counts. The first pipeline was a branched version of ScreenProcessing (https://github.com/ucsf-lgr/ScreenProcessing) developed by the Weissman laboratory^52^ using hitThreshold of 4. The second was MAGeCK-Vispr^53,54^.

### Network analysis of screen hits

Significant (*P* < 0.05) sensitizing (score < 0) hits from sgNT and sg*NPC1* screens were combined (and likewise for protective (score > 0) hits). Coessentiality data from DepMap (22Q2, Achilles_gene_effect.csv) was pre-processed to obtain locus-adjusted correlations (Mendillo laboratory; https://github.com/mendillolab/fireworks/tree/master/generate_corr_matrices)^55^. A custom Python script (https://github.com/claire-goul/local_DepMapandBioGrid) was written to obtain the top correlated genes to gene hits (using the default parameters of threshold > 0.2 and num = 3). Physical protein–protein interactions for gene hits were obtained from the BioGrid website (Biogrid_MV-Physical_4.4.243_Human; https://downloads.thebiogrid.org/BioGRID/Release-Archive/BIOGRID-4.4.243/), using default parameters (minimum number of citations; that is, numcitations parameter, equal to 2)^56^. Biogrid interactions were then mapped onto the correlated genes obtained from the gene hits, and networks containing at least 2 gene hits and at least 3 nodes per network were plotted in Cytoscape (v3.10.3). Genes implicated in neurodegenerative disease were downloaded from the Monarch Initiative (https://monarchinitiative.org/MONDO:0005559#causal-gene)^57^ and overlaid manually onto the Cytoscape networks by importing the downloaded file into Cytoscape. A custom Python script (Python v3.9.6) was written to cluster networks in communities using the Python Louvain package (https://github.com/taynaud/python-louvain), using a resolution value of 2. Communities and the top Gene Ontology term (https://geneontology.org/) for each community were manually annotated on Cytoscape networks.

### Growth curves

Cells were seeded in 96-well plates at 10,000 cells per well in 100 μl complete medium, and the following day they were treated with 100 μl of LLOMe (2× concentration) prior to imaging, with images of each well taken every 4 h, on Incucyte S3 with a 10× objective (Sartorius).

### Confocal imaging of live cells

Live imaging of eGFP–UBAP1, EAP30–mNeonGreen, PDCD6IP–mNeonGreen, CHMP4B–mNG and mCherry–Gal3, as well as endogenously tagged mNG lines, was performed on OperaPhenix (Perkin Elmer). Two days prior to imaging, 96-well plates were coated overnight in fibronectin, and cells were seeded the following day with 30K–40K cells per well onto 96-well CellVis (P96-1.5H-N) or Greiner Screenstar COC plates (655866) in 100 μl phenol red-free DMEM. The next day, 50 μl of Hoescht dye was added to cells to stain nuclei 30 min before the start of the experiment. Imaging of non-overlapping frames (at minimum 4 per well) were taken; first, the nuclei were imaged, then cells were imaged at baseline, followed by addition of the indicated concentrations of either LLOMe (or DMSO vehicle control), and imaged every 4−6 min. Temperature and CO_2_ levels were maintained at 37 °C and 5%, respectively, during the experiment. All images were taken with a 40× water objective. Image analysis of number of spots per cell was performed on Harmony 3D analysis software (v5.3) for nuclei and spot selection and masking and quantification of nuclei/spot number and spot relative intensity for all frames and time points collected.

For i3 neurons: At day 4 post-differentiation, i3 neurons plated in 24-well glass bottom plates (CellVis, P24-1.5H-N) were transduced with concentrated mCherry–Gal3 or CHMP4B–mNG lentivirus resuspended in BrainPhys neuronal medium, and 24 h later changed into fresh BrainPhys medium. Live imaging was performed 72–96 h post-transduction on OperaPhenix (Perkin Elmer). Prior to live imaging, medium was changed into BrainPhys neuronal medium lacking Phenol Red.

### Immunofluorescence staining and confocal imaging

Cells were seeded on fibronectin-coated glass coverslips in 12-well plates the day before the experiment and treated with the indicated DMSO or LLOMe concentrations. Cells were then rinsed one time with PBS, permeabilized with 0.1% (w/v) saponin in PBS for 10 min at room temperature (or, for LC3B immunofluorescence, at −20 °C in ice-cold 100% methanol for 5 min) and rinsed once with PBS. Primary antibodies were diluted at 1:300 concentration in 5% normal donkey serum (Jackson ImmunoResearch, 017-000-121) and incubated for 1 h at room temperature. Coverslips were rinsed 3 times with DPBS and then labelled with fluorescently conjugated secondary antibodies (diluted 1:400 in 5% normal donkey serum in DPBS) for 1 h at room temperature, protected from light. Coverslips were rinsed with PBS four times and mounted on glass slides using VECTASHIELD Antifade Mounting Medium with or without DAPI (Vector Laboratories, H-1200 or H-1000). Confocal microscopy was performed on a spinning-disk Nikon Ti-E inverted microscope (Nikon Instruments) with Andor Zyla-4.5 cMOS camera (Andor Technology) using iQ3 acquisition (v3.6) and Andor Fusion software (v1.1.1) (Andor Technology). All images were taken with a 60× oil objective. Colocalization analysis from immunofluorescence was performed by importing raw, unprocessed, non-overlapping images to FIJI (v2.1.0/1.53c) or ImageJ (v2.16). Images of individual channels were thresholded independently to exclude background and converted to binary mask. Colocalization between lysosomes (LAMP2) and marker of interest (TFG or SEC31A) or TFG and ERES (SEC31A) was determined using the AND function of the image calculator. Confocal images of contact sites between TFG and LAMP were acquired using a 100× oil objective on a BC43 Andor Spinning-disk confocal microscope, and images shown in figures were pre-processed using deconvolution with Imaris Quant software (v10.2).

### Immuno-electron microscopy

Cells in culture dishes were initially fixed by incubation for 5 min with an equal volume of 2% paraformaldehyde in 0.1 M phosphate buffer, mixed with the culture medium to minimize osmotic stress. The solution was then replaced with freshly prepared 4% paraformaldehyde in the same buffer, and fixation was continued for 2 h at room temperature. After fixation, samples were washed three times in PBS, and residual aldehydes were quenched by incubation with 0.15% glycine in PBS for 10 min. Cells were scraped in PBS supplemented with 1% gelatin and transferred to microcentrifuge tubes. Next, gelatin concentration was increased to 12%, and samples were incubated for 10 min at 37 °C. Cells were collected by centrifugation, and the gelatin-embedded cell pellets were allowed to solidify on ice for 30 min. Solidified pellets were trimmed into small blocks and cryoprotected by infiltration with 2.3 M sucrose overnight at 4 °C. The blocks were then mounted onto specimen pins and stored in liquid nitrogen until further processing.

For cryo-ultramicrotomy, sucrose-infiltrated, gelatin-embedded samples were sectioned at a nominal thickness of 80 nm at −110 °C using a diamond knife (DiATOME) on an ultracryomicrotome (Leica). Ultrathin sections were collected on Formvar- and carbon-coated TEM grids using a pickup solution consisting of a 1:1 mixture of 2.3 M sucrose and 1.8% methylcellulose.

For immunogold labelling, sections on the grids were first incubated in PBS at 37 °C for approximately 30 min to remove residual embedding material, followed by washing in PBS containing 0.15% glycine. Nonspecific binding was blocked by incubation in PBS supplemented with 0.5% fish skin gelatin (FSG; Sigma) and 0.1% BSA (BSA-c; Aurion). Sections were incubated with a primary antibody against TFG (ab156866; 1:30 dilution), followed by with Protein A conjugated to 10-nm colloidal gold particles (Cell Microscopy Core, UMC Utrecht). Post-labelling fixation was performed using 1% glutaraldehyde. Grids were rinsed six times in Milli-Q water and contrasted for 5 min with 2% uranyloxalate-acetate (pH 7), and 10 min with uranylacetate/methylcellulose (pH 4) on ice. Finally, grids were picked up using the loop-out method with a rhenium wire loop and air-dried for 30 min prior to imaging.

Imaging was performed on a TECHNAI12 transmission electron microscope (Thermo Fisher) operated at 80 kV. For each condition, images were acquired from two independent grids. A more detailed description of the immunolabelling procedure has been reported previously^58^.

### Protein purification

Human codon-optimized truncated 6×His-TEV-mCherry TFG (WT, ΔPB1, LIR2-mut, ΔCC, PSAP, R106C mutant; residues 1–210) was expressed in *Escherichia coli* Rosetta (DE3) pLysS cells. Bacteria were grown in Luria broth (LB) medium until OD_600_ ≈ 0.8–1, induced with 0.5 mM isopropylthiogalactoside at 20 °C for 18 h. Cell pellets from 0.5 l of culture were frozen in liquid nitrogen, and subsequently resuspended in lysis buffer (50 mM 4-(2-hydroxyethyl)-1-piperazineethanesulfonic acid (HEPES) at pH 7.5, 500 mM KCl, 10 mM imidazole, 2 mM MgCl_2_, complete protease inhibitor (Roche)) followed by lysis with a tip sonicator (4× 50 mA; 30 s on/1 min off). Lysates were cleared by ultracentrifugation at 24,000*g* for 30 min at 4 °C (Beckman, Ti45 rotor). Cleared supernatants were applied to Ni-NTA resin (Qiagen, 30230) pre-washed in lysis buffer and incubated with rotation at 4 °C for 1 h, and the mixture was applied to a gravity column. Following washes with 15 bead volumes of lysis buffer, 6×His-tagged TFG constructs were rapidly eluted in 4 bead volumes of lysis buffer containing 300 mM imidazole. Elution was evaluated by Coomassie staining, flash frozen in liquid nitrogen, and stored at −80 °C.

Human 6×His-TEV-mCherry-P62 (Addgene #199780) was purified using the same protocol as for TFG, with additional separation on a HiLoad 16/600 Superdex column (Cytiva). Protein was eluted in buffer containing 25 mM HEPES pH 7.5, 500 mM KCl, and 1 mM DTT, protein containing fractions were pooled and concentrated with an Ultra Centrifugal Filter (50 kDa MWCO, Amicron), and frozen in liquid nitrogen.

Human 6×His–TSG101 UEV domain (WT, M95A, N45A or M95A/N45A) was expressed in *E. coli* strain BL21(DE3) Star cells, and bacteria were grown in Luria broth (LB) medium until OD600 ≈ 0.5, induced with 1 mM isopropylthiogalactoside at 37 °C for 4 h. Cell pellets from 0.5 l of culture were frozen in liquid nitrogen, and subsequently resuspended in lysis buffer (50 mM HEPES at pH 7.5, 150 mM NaCl, 2 mM MgCl_2_, complete protease inhibitor (Roche)), followed by lysis with a tip sonicator (4× 50 mA; 30 s on/1 min off). Lysates were cleared by ultracentrifugation at 24,000*g* for 30 min at 4 °C (Beckman, Ti45 rotor). Cleared supernatant was applied to GST resin pre-washed in lysis buffer and incubated with rotation at 4 °C for 30 min. Following washes with 10 bead volumes of lysis buffer, 6×His-tagged TFG constructs were eluted in 2 bead volumes of elution buffer (lysis buffer plus 20 mM glutathione, freshly made to pH 8) with 1× 10-min elution and 1× overnight elution. Elutions were evaluated by Coomassie staining, pooled, concentrated on an Amicron 30 kDA MWCO column, flash frozen in liquid nitrogen, and stored at −80 °C.

### Plasmids and cloning

Human codon-optimized truncated 6×His-TEV-mCherry TFG (WT, deltaPB1, LIR2MUT, deltaCC, PSAP, R106C mutant; residues 1–210) was cloned into pETDuet-His-TEV-mCherry (Addgene #199780). Human pGST2-TSG101 UEV plasmid was obtained from Addgene (Addgene #184810) and cDNA for M95A, N45A, M95A/N45A (Twist Biosciences) was subcloned into the same plasmid. pCDH-CHMP4B–mNG-3×HA IRES-Blast and pCDH-mCherry-GAL3-IRES-Blast were received from the laboratory of H. Stenmark, and human codon-optimized ALIX or EAP30 cDNA (Twist Biosciences) was subcloned into the digested pCDH-mNeonGreen-3×HA IRES Blast vector. Human codon-optimized UBAP1 cDNA (Twist Biosciences) was subcloned into the pLJM1 eGFP Hygro vector. For rescue construct cloning, TFG human codon-optimized cDNA (WT, R22W, R106C, P285L, LIR2 mutant, LIR3 mutant, double LIR mutant, deltaPB1 and deltaCC; IDT and Twist Biosciences) were cloned into pLJM1 Hygro plasmid using Gibson assembly along with an IRES-mCherry cDNA fragment.

Short-hairpin oligonucleotides (shRNAs) directed against *TFG* (TRCN0000078660), *TSG101* (TRCN0000007563), *PDCD6IP* (TRCN0000029395), *ATG5* (TRCN0000151474), *PDCD6* (TRCN0000053388, TRCN0000303757, TRCN0000303818), *ATG13* (TRCN0000172801), *FIP200* (TRCN0000315347) and luciferase (TRCN0000072243, used as a non-targeting control), were cloned into the pLKO.1 lentiviral vector (The RNAi Consortium, Broad Institute) according to the manufacturer’s instructions. *TFG* sgRNA sequences (sg1: TCAGCGGAGACCTGCGGAGG; sg2: CTCGCGTCCGCCCATTCAGG) were cloned into the pLG15 vector (Kampmann laboratory).

### Peptide binding and fluorescence polarization

A binding curve of FAM-p62 LIR peptide (SGGDDDWTHLSSKEVDl; 12.5 nM final) with recombinant LC3B or GABARAP was generated by incubating FAM-p62 LIR peptide with serially twofold-diluted recombinant LC3 or GABARAP (starting at 50 nM), in triplicate. Fluorescence polarization of FAM-peptide was measured on a Tecan Spark Multimode Microplate reader. Recombinant LC3B or GABARAP (5 μM final) was incubated with FAM-p62 LIR peptide (12.5 nM final) for 30 min, followed by incubation with 3 mM or serially twofold-diluted unlabelled p62-LIR peptide, TFG-LIR2 wild-type (AASMSAFDPLKNQDEI) or LIR3 wild-type (YQPRPGFTSLPGSTMT), TFG LIR2-mutant (AASMSAADPAKNQDEI) or LIR3-mutant (YQPRPGATSAPGSTMT) peptides, in triplicate for 15 min, followed by fluorescence polarization measurement. All peptides were ordered from GenScript. Curves were plotted in Prism v10.2.0 (GraphPad), and Kis were calculated using the Prism One site fit ki algorithm, while Kds were calculated using the Prism One site total and nonspecific binding algorithm.

### Mammalian cell culture

Human 293T sgNT and sg*NPC1* knockout cell lines were generated using Cas9/sgRNA ribonuclear protein transfection (sg*NPC1* sgRNA sequence: GCTGCTACTGTGTCCAGCGC) as previously described^26^. HEK293T, HeLa and U2OS cells and their derivatives, including HEK293T cells with ESCRT components tagged at their endogenous loci with mNG^59^, were cultured in DMEM base medium with 10% fetal bovine serum supplemented with 2 mM glutamine, penicillin and streptomycin. All cell lines were maintained at 37 °C and 5% CO_2_. The cells were free of mycoplasma contamination and routinely checked using mycoplasma PCR Detection kit (abm, G238) and/or DAPI staining. All wild-type parental cell lines, with the exception of the patient fibroblasts, were obtained from and authenticated by the UC Berkeley cell culture facility.

All K562 cell lines were maintained in Roswell Park Memorial Institute (RPMI) 1640 medium (Gibco, 11875085) supplemented with 10% FBS (Avantor Seradigm, 97068-085), 1% Penicillin-Streptomycin (Gibco, 15140122), and 1% Glutamax (Gibco, 35050-061) All cells were cultured at 37 °C in a humidified atmosphere containing 5% CO_2_. Cells were incubated in shaking conditions for the duration of the screen.

Human i3 neurons were obtained from M. Ward and cultured and differentiated according to the described protocol^60^. CRISPRi-i3 iPSCs containing NGN2-inducible cassette and dCas9-BFP-KRAB were a gift from M. Ward^61,62^. CRISPRi-i3 iPSCs were differentiated as described^60^. In brief, iPSCs were dissociated with Accutase (Gibco, A1110501) and seeded onto Matrigel (Corning, 354277) coated six-well plates. iPSCs were infected with lentiviruses concentrated in essential-8 medium (Gibco, A1516901) expressing sgNT or sgTFG. After 24 h, medium was replaced with fresh medium containing puromycin (1 μg ml^−1^). Medium was replaced daily with fresh medium containing puromycin for 72 h. After selection, cells were expanded for one passage before dissociating and seeding cells into induction medium containing N2 Supplement (Gibco, 17502048) in Knockout DMEM/F:12 (Thermo Fisher, 12660012) with non-essential amino acids (Gibco, 11140050), GlutaMax (Gibco, 35050-061) and doxycycline (2 μg ml^−1^, 35050-061). Medium was replaced with fresh induction medium for 72 h, before dissociating cells and seeding onto PLO (Sigma, P3655)/Laminin (R&D Systems, 3446-005-01) coated plates (prepared as described previously^60^) containing BrainPhys neuronal differentiation medium (StemCell Technologies, 5790) supplemented with B27+ (Gibco, A3582801) containing BDNF (Preprotech, 450-02), NT-3 (Preprotech, 450-03) and GDNF (Preprotech, 450-10). i3Neurons were given 60% medium changes (remove 40%, add back 60%) every 3 days until collected for experiments.

Patient fibroblasts were obtained from E. Lamantea^63^ and cultured in DMEM with 10% fetal bovine serum supplemented with 2 mM Glutamine, penicillin and streptomycin.

### Lentivirus production and infection

Lentiviruses were made by co-transfecting pLJM1, pCDH, pLenti-sgRNA or pLKO constructs with psPAX2 and pMD2G packaging plasmids into HEK293T cells using PEI transfection reagent. Viral supernatant was collected 48–72 h post-transfection and filtered using a 0.45-µm syringe filter. The virus was then concentrated using Lenti-X concentrator (Clontech 631231) according to manufacturer’s protocol and stored at −80 °C. For lentivirus infection, target cells were seeded in 6-well plates and infected the following day with lentivirus and 10 µg ml^−1^ Polybrene (Millipore TR-1003-G). After 24 h, virus was removed, and cells were changed into fresh medium containing either puromycin (1 μg ml^−1^), blasticidin (5 μg ml^−1^) or hygromycin B (200 μg ml^−1^) for selection. Experiments on shRNA infected cells were performed 4–5 days post-transduction. For neuron experiments, mCherry–Gal3 virus was concentrated and resuspended in BrainPhys neuronal differentiation medium containing the described supplements, and neurons were infected 3 days prior to the experiment with concentrated virus (no Polybrene), and medium was changed into fresh BrainPhys (containing no antibiotics) the day following infection.

### Immunoprecipitation

For GST–UEV immunoprecipitations with cell lysate, cells were rinsed once with PBS and lysed in lysis buffer (PBS, 1% NP-40, 1 mM DTT, 5 mM EDTA, EDTA-free protease inhibitor tablet) rotating for 15 min at 4 °C. Cell lysates were cleared by centrifugation in a microcentrifuge at 13,000 rpm for 10 min at 4 °C. Well mixed glutathione sepharose 4B resin slurry (Cytiva, 17-0756-05) was washed once in lysis buffer, then incubated with recombinant UEV domain (12 μl of GST slurry with lysis buffer containing 100 μg of UEV domain) for 30 min rotating at 4 °C. GST–UEV or empty beads were washed 3 times with lysis buffer, then incubated with cleared supernatant for 1 h at 4 °C, followed by 3 washes with lysis buffer and resuspended in a 1:1 ratio of 5× sample buffer and lysis buffer. Following incubation at 95 °C for 5 min, immunoprecipitated proteins were resolved by SDS–PAGE and analysed by immunoblotting.

### Dextran assay for lysosomal deacidification

For dextran experiments in patient fibroblasts, cells were incubated with fluorescently labelled dextrans 16–20 h prior to imaging. Specifically, 10 kDa dextran conjugated to Alexa Fluor 647 (dextran−647, Thermo Fisher D22914) and 40 kDa dextran conjugated to fluorescein isothiocyanate (dextran-Fluorescein, Thermo Fisher D1845) were added to the culture medium at a final concentration of 25 μg ml^−1^ each. Two hours before imaging, cells were washed two times with phosphate-buffered saline (PBS) to remove extracellular dextran, then incubated in phenol red-free Dulbecco’s modified Eagle’s medium (DMEM, Thermo Fisher 31053-028) for the chase period.

### Equilibrium binding assay using visual immunoprecipitation

For TSG101-UEV visual immunoprecipitation experiments, 90 μl glutathione sepharose 4B resin slurry (Cytiva, 17-0756-05) was washed with excess buffer (500 mM KCl, 50 mM HEPES), and beads were resuspended in 90 μl buffer. Ten micrograms recombinant GST-tagged UEV protein per μl of bead suspension was added and incubated with beads for 15 min nutating at 4 °C. Beads were spun down (1,200*g* for 2 min), washed once in excess buffer, and resuspended in 90 μl buffer.

For Flag–GFP–LC3B visual IP experiments, an 80–100% confluent plate of HEK293T cells expressing Flag–GFP–LC3B were resuspended in lysis buffer (50 mM HEPES at pH 7.3, 500 mM KCl, 1% Triton X-100 and protease inhibitor cocktail). Lysates were nutated for 15 min at 4 °C, after which unlysed precipitates were removed by spinning the lysates at 13,000 rpm for 10 min at 4 °C. Meanwhile, 30 μl of GFP-Trap agarose beads were washed with 500 μl of lysis buffer. Incubate washed beads with lysate for 30 min at 4 °C. After incubation, wash coated beads once with bulk lysis buffer and twice with wash buffer (50 mM HEPES at pH 7.3, 500 mM KCl) and resuspend beads in 60 μl wash buffer.

Three microlitres of UEV-coated GST beads or LC3B-coated agarose beads were incubated with 10 μl of recombinant TFG protein (at indicated concentration of 5 μM or lower) and samples were spotted onto glass slides and covered with glass coverslips. Confocal microscopy was performed on a spinning-disk Nikon Ti-E inverted microscope (Nikon Instruments) with Andor Zyla-4.5 cMOS camera (Andor Technology) using iQ3 acquisition software (Andor Technology). All images were taken with a 10× objective.

### Statistical analysis and graphs

Statistical analyses were performed using unpaired two-tailed Student’s *t*-tests for comparisons of two groups, one-way analysis of variance (ANOVA) or two-way ANOVA for group comparisons using Prism (v10.2.0, GraphPad). Details of each statistical test are given in the legend accompanying each figure. Curve fitting for enzyme kinetics and other statistical analyses for peptide binding and polarization experiments are detailed in the appropriate methods. Graphs were plotted with Prism (v10.2.0, Graphpad), or RStudio (v1.4.1717). All data are displayed as mean ± s.d. All *t*-tests were two-tailed, unpaired; one-way ANOVAs used Dunnett’s multiple comparisons test with a single pooled variance for comparison to control sample, or for comparisons among all pairs, Tukey’s test; all two-way ANOVAs used used Dunnett’s multiple comparisons test with a single pooled variance for comparison to control sample, or for comparisons among all pairs, Šidák multiple comparisons test with a single pooled variance.

### Reporting summary

Further information on research design is available in the Nature Portfolio Reporting Summary linked to this article.

## Online content

Any methods, additional references, Nature Portfolio reporting summaries, source data, extended data, supplementary information, acknowledgements, peer review information; details of author contributions and competing interests; and statements of data and code availability are available at 10.1038/s41586-026-10604-6.

## Supplementary information

Supplementary Figure 1 Full versions of all western blots and gels with molecular size markers

Reporting Summary

Supplementary Table 1 All statistically significant hits from the genome-wide CRISPRi screen in sgNT or sg*NPC1* cells

Peer Review file

## Source data

Source Data Fig. 1

Source Data Fig. 2

Source Data Fig. 3

Source Data Fig. 4

Source Data Fig. 5

Source Data Extended Data Fig. 1

Source Data Extended Data Fig. 3

Source Data Extended Data Fig. 4

Source Data Extended Data Fig. 5

Source Data Extended Data Fig. 6

Source Data Extended Data Fig. 7

Source Data Extended Data Fig. 8

Source Data Extended Data Fig. 9

Source Data Extended Data Fig. 10

## Data Availability

CRISPRi screen data are provided in Supplementary Table 1 and raw data are available at NCBI Gene Expression Omnibus (GEO) under accession GSE328484. Full versions of all blots and gels can be found in Supplementary Information Fig. 1. Data obtained from publicly available sources used in this paper were coessential genes from DepMap (22Q2, Achilles_gene_effect.csv), protein interactions (Biogrid_MV-Physical_4.4.243_Human; https://downloads.thebiogrid.org/BioGRID/Release-Archive/BIOGRID-4.4.243/), neurodegenerative causal genes from Monarch Initiative (https://monarchinitiative.org/MONDO:0005559#causal-gene) and Gene Ontology terms (https://geneontology.org/). Source data are provided with this paper.

## Code availability

The Python script for gene correlation analysis can be found at https://github.com/claire-goul/local_DepMapandBioGrid and enabled identification of relevant hits from the CRISPRi screen.
