# Supplementary material for: LASER couples damage sensing to ESCRT assembly for lysosome repair
Source: Nature. 2026 Jun 3;656(8126):216–26. doi: 10.1038/s41586-026-10604-6 (PMC13442042; doi:10.1038/s41586-026-10604-6)
Supplement: Supplementary file 1 [file 41586_2026_10604_MOESM1_ESM.pdf]

nature portfolio

https://doi.org/10.1038/s41586-026-10604-6

## Supplementary information

# LASER couples damage sensing to ESCRT assembly for lysosome repair

In the format provided by the authors and unedited

**Western blot - Source data - Page 1**

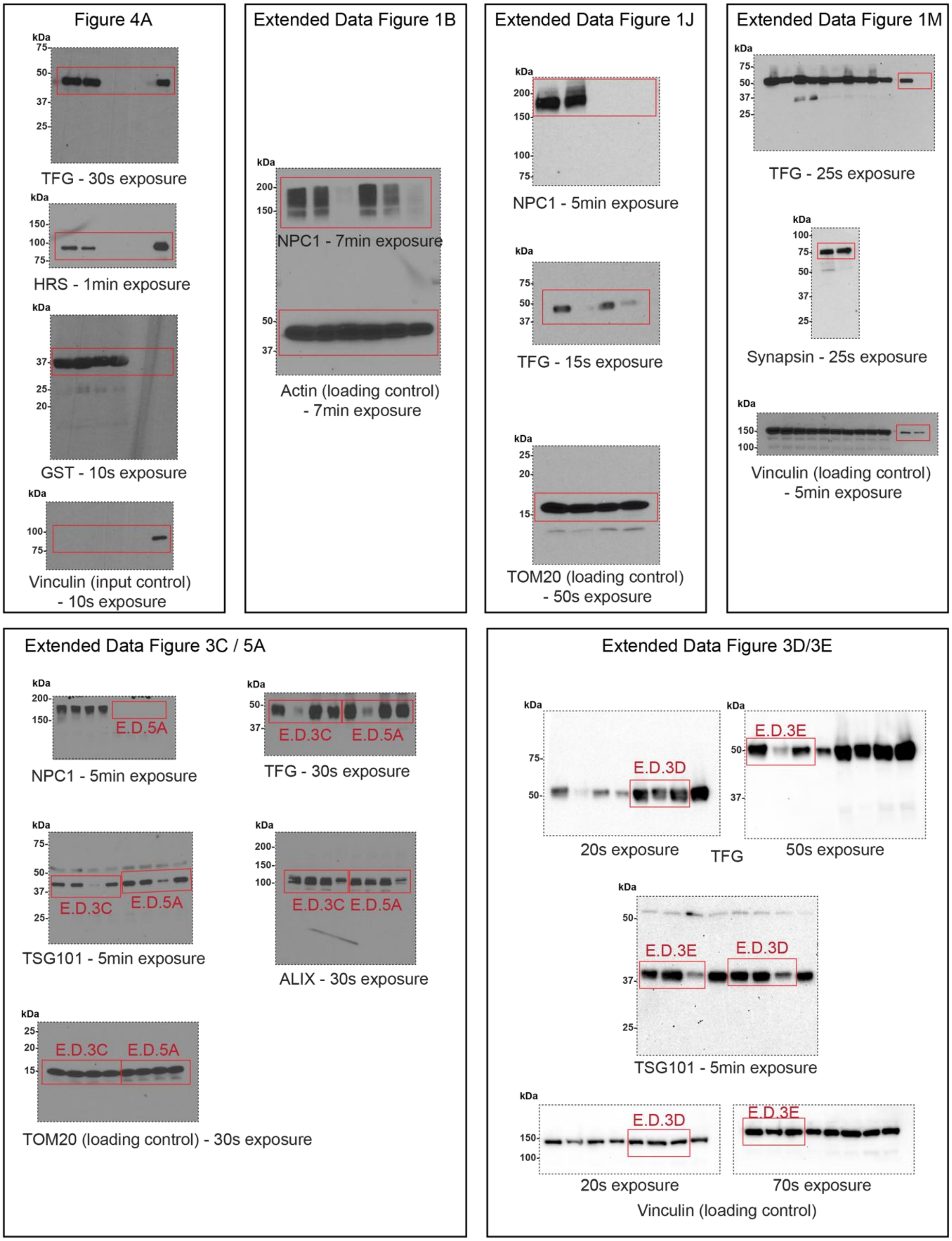

**Western blot - Source data - Page 2**

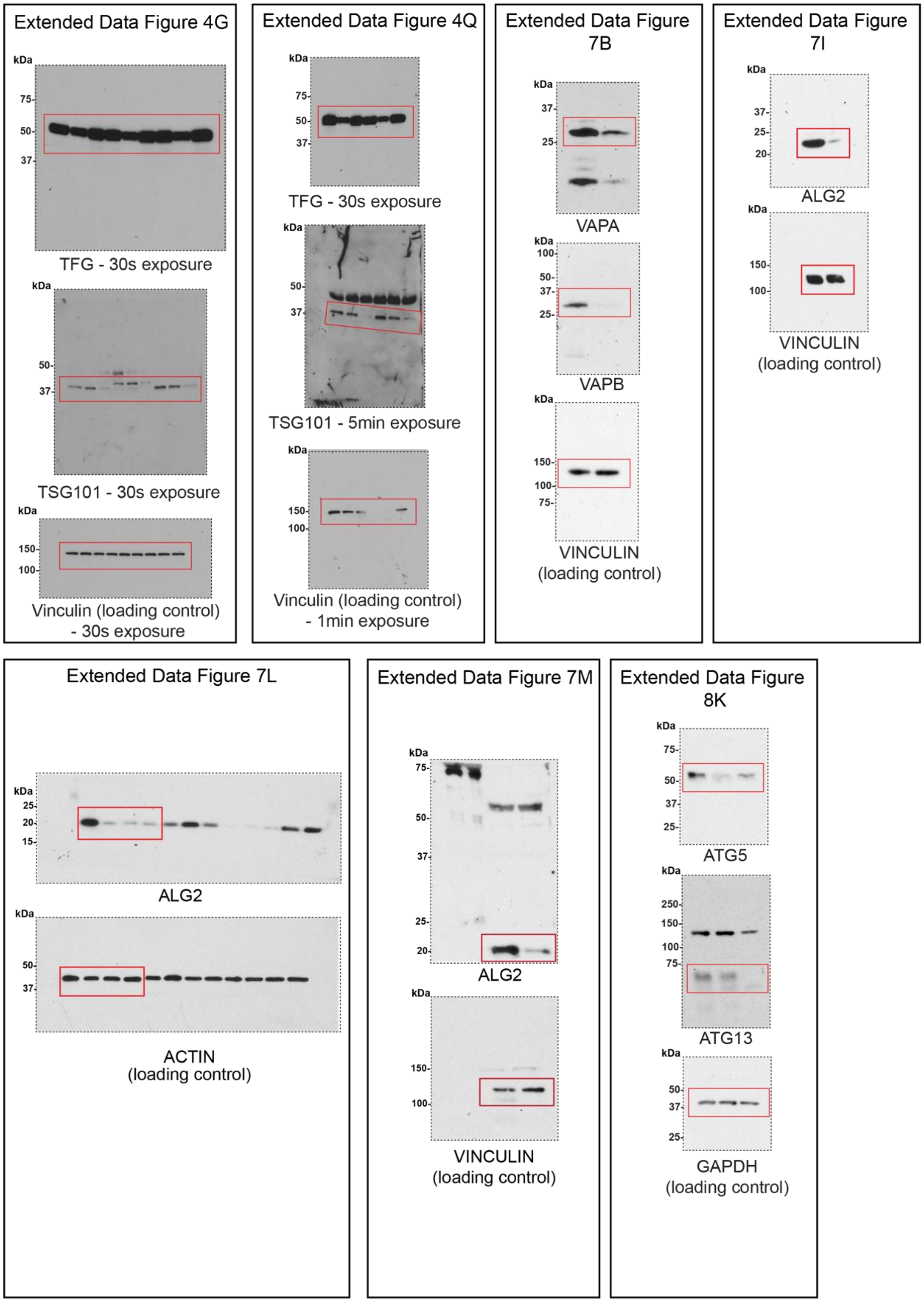


**Supplementary Information Figure 1.** Full versions of all western blots and gels with molecular weight markers. Red boxes are crops included in the paper.
