# Supplementary material for: LASER couples damage sensing to ESCRT assembly for lysosome repair
Source: Nature. 2026 Jun 3;656(8126):216–26. doi: 10.1038/s41586-026-10604-6 (PMC13442042; doi:10.1038/s41586-026-10604-6)
Supplement: Supplementary file 2 [file 41586_2026_10604_MOESM2_ESM.pdf]

nature portfolio

Corresponding author(s): Aakriti Jain, Roberto Zoncu

Last updated by author(s): 4/23/2026

Reporting Summary

Nature Portfolio wishes to improve the reproducibility of the work that we publish. This form provides structure for consistency and transparency in reporting. For further information on Nature Portfolio policies, see our Editorial Policies and the Editorial Policy Checklist.

Statistics

For all statistical analyses, confirm that the following items are present in the figure legend, table legend, main text, or Methods section.

n/a	Confirmed	
☐	☒	The exact sample size (*n*) for each experimental group/condition, given as a discrete number and unit of measurement
☐	☒	A statement on whether measurements were taken from distinct samples or whether the same sample was measured repeatedly
☐	☒	The statistical test(s) used AND whether they are one- or two-sided<br>*Only common tests should be described solely by name; describe more complex techniques in the Methods section.*
☐	☒	A description of all covariates tested
☐	☒	A description of any assumptions or corrections, such as tests of normality and adjustment for multiple comparisons
☐	☒	A full description of the statistical parameters including central tendency (e.g. means) or other basic estimates (e.g. regression coefficient) AND variation (e.g. standard deviation) or associated estimates of uncertainty (e.g. confidence intervals)
☐	☒	For null hypothesis testing, the test statistic (e.g. *F*, *t*, *r*) with confidence intervals, effect sizes, degrees of freedom and *P* value noted<br>*Give P values as exact values whenever suitable.*
☒	☐	For Bayesian analysis, information on the choice of priors and Markov chain Monte Carlo settings
☒	☐	For hierarchical and complex designs, identification of the appropriate level for tests and full reporting of outcomes
☒	☐	Estimates of effect sizes (e.g. Cohen's *d*, Pearson's *r*), indicating how they were calculated

Our web collection on statistics for biologists contains articles on many of the points above.

Software and code

Policy information about availability of computer code

Data collection	Incucyte S3 (Sartorius), OperaPhenix (PerkinElmer), spinning-disk Nikon Ti-E inverted microscope (Nikon) with Andor Zyla-4.5 cMOS camera (Andor Technology), Spark Multimode Microplate reader (Tecan), AKTA Pure (Cytiva), BC43 Spinning Disk Confocal microscope (Andor Technology)
Data analysis	RStudio (v 1.4.1717), ImageJ (v2.16), FIJI (v2.1.0/1.53c), Harmony3D Software (PerkinElmer, v5.3), GraphPad Prism (v10), Cytoscape (3.10.3), Python (3.9.6), MAGeCKVispr<br>and ScreenProcessing (GitHub), python-louvain (Github), Andor Fusion (v1.1.1), AKTA Pure Unicorn (Cytiva), Andor iQ3 (v3.6), local_DepMapandBiogrid (https://github.com/claire-goul/local_DepMapandBioGrid), Imaris Quant Software (v10.2)

For manuscripts utilizing custom algorithms or software that are central to the research but not yet described in published literature, software must be made available to editors and reviewers. We strongly encourage code deposition in a community repository (e.g. GitHub). See the Nature Portfolio guidelines for submitting code & software for further information.

Data

Policy information about availability of data

All manuscripts must include a data availability statement. This statement should provide the following information, where applicable:

- Accession codes, unique identifiers, or web links for publicly available datasets
- A description of any restrictions on data availability
- For clinical datasets or third party data, please ensure that the statement adheres to our policy

Source data are provided with this paper in the Supplementary Information . CRISPRi Screen data are provided in Supplementary Table 1 and raw data are available on NCBI GEO (Accession: GSE328484). Source data for all graphs are provided in Extended Data files. Full versions of all blots and gels can be found in Supplementary Information Fig. 1. Data obtained from publicly available sources used in this paper were coessential genes from DepMap (22Q2, Achilles_gene_effect.csv), protein interactions (Biogrid_MV-Physical_4.4.243_Human; https://downloads.thebiogrid.org/BioGRID/Release-Archive/BIOGRID-4.4.243/), neurodegenerative causal genes from monarch initiative (https://monarchinitiative.org/MONDO:0005559#causal-gene), and GO terms (https://geneontology.org/).

Research involving human participants, their data, or biological material

Policy information about studies with human participants or human data. See also policy information about sex, gender (identity/presentation), and sexual orientation and race, ethnicity and racism.

Reporting on sex and gender	N/A
Reporting on race, ethnicity, or other socially relevant groupings	N/A
Population characteristics	N/A
Recruitment	N/A
Ethics oversight	N/A

Note that full information on the approval of the study protocol must also be provided in the manuscript.

Field-specific reporting

Please select the one below that is the best fit for your research. If you are not sure, read the appropriate sections before making your selection.

☒ Life sciences ☐ Behavioural & social sciences ☐ Ecological, evolutionary & environmental sciences

For a reference copy of the document with all sections, see nature.com/documents/nr-reporting-summary-flat.pdf

Life sciences study design

All studies must disclose on these points even when the disclosure is negative.

Sample size	No sample size calculation was performed. Each imaging experiment was performed with at least 2 biological replicates and multiple fields of view per sample were acquired as indicated in figure legends. Sample size for all Opera imaging experiments were chosen to maximize number of images taken given the time interval needed (5 minutes or less) for high resolution imaging of our reporters. For each IF experiment, each coverslip was prepared with 2 technical replicates. For growth curves and binding assays, we performed at least 2 technical replicates per experiment (limited by plate size or number of conditions).
Data exclusions	No data exclusions were made.
Replication	All experiments were repeated in technical replicates, as indicated in the figure legends. All major experiments were performed with at least 3 biological replicates.
Randomization	No in vivo data was acquired in this study. All experiments were conducted exclusively in cell culture without the use of animal subjects- randomization mitigates bias due to heterogeneity among subjects, and here all cells used were from the same parental line at the same passage. Treatment conditions were assigend according to a predetermined layout and processed under identical controlled parameters (temperature, CO2, media, seeding density, passage number), eliminating much of the heterogeneity randomization is intended to address. We hope this addresses the concern and are happy to provide further clarification if needed.
Blinding	This study was conducted entirely in cell culture without animal subjects. All experiments were performed by automated or semi-automated methods (automated imaging on Opera phenix, plate reader, or incucyte; or random selection of fields of view to image in immunofluorescence experiments), precluding the observer bias that blinding is designed to address.

Reporting for specific materials, systems and methods

We require information from authors about some types of materials, experimental systems and methods used in many studies. Here, indicate whether each material, system or method listed is relevant to your study. If you are not sure if a list item applies to your research, read the appropriate section before selecting a response.

Materials & experimental systems

n/a	Involved in the study
☐	☒ Antibodies
☐	☒ Eukaryotic cell lines
☒	☐ Palaeontology and archaeology
☒	☐ Animals and other organisms
☒	☐ Clinical data
☒	☐ Dual use research of concern
☒	☐ Plants

Methods

n/a	Involved in the study
☒	☐ ChIP-seq
☒	☐ Flow cytometry
☒	☐ MRI-based neuroimaging

Antibodies

Antibodies used

Antibodies used were: against HA (Catalog # 3724S, Clone C29F4; 1:1000 dilution for Western Blot), LC3B (Catalog # 3868S, Clone D11; 1:1000 dilution for Western Blot), VINCULIN (Catalog # 13901S, Clone E1E9V; 1:1000 dilution for Western Blot), LAMTOR4 (Catalog # 13140S, Clone D4P60; 1:300 dilution for IF), HRS (Catalog # 15087S, Clone D7T5N; 1:1000 dilution for Western Blot), TOM20 (Catalog # 42406S, Clone D8T4N; 1:1000 dilution for Western Blot), ATG5 (Catalog # 2630S; 1:1000 dilution for Western Blot), ATG13 (Catalog # 13468S, Clone E1Y9V; 1:1000 dilution for Western Blot) from Cell Signaling Technology; LAMP2 (Catalong # sc-18822, Clone H4B4; 1:300 dilution for IF), CHMP1A (Catalog # sc-271617, Clone B-5; 1:300 dilution for IF) from Santa Cruz Biotechnology; TFG (Catalog # ab156866, Clone EPR8766; 1:300 dilution for IF, 1:1000 dilution for Western Blot) and NPC1 (Catalog # ab36983; 1:1000 dilution for Western blot) from Abcam; SEC31A (Catalog # 612350, Clone 32; 1:300 dilution for IF) from BD Biosciences; SEC31A (Catalog # 17913-1-AP;1:300 dilution for IF), TSG101 (Catalog # 67381-Ig, Clone 2B7G8; 1:1000 dilution for Western blot), ALIX (Catalog # 67715-Ig, Clone 1H9D9; 1:1000 dilution for Western blot), PDCD6/ALG2 (Catalog # 12303-1-AP; 1:1000 dilution for Western blot) from Proteintech; TFG (Catalog # NBP2-62212, Clone TFG-03; 1:300 dilution for IF) from Novus

Validation

Cell Signaling Technology (CST): CST states that it tests antibodies in several applications including western blotting (WB), immunofluorescence (ICC/IF), immunohistochemistry (IHC), flow cytometry (FC), and ELISA, and that it has passed rigorous testing standards when it is recommended for a particular application.
The HA-Tag (C29F4) antibody (#3724S) is validated on the CST website by western blot (WB) in COS-7 cells expressing HA-tagged Akt. This antibody has 31 citations, with papers displaying validation by western blot of cells expressing HA tagged proteins.
The LC3B (D11) antibody (#3868S) is validated on the CST website by WB and IF of HCT116 or HCT116 LC3B knockout cells treated with or without chloroquine, and FC of HCT116 or HCT117 LC3B knockout cells. This antibody has 2076 citations on the CST website that have displayed validation by WB, IF, IHC, ICC, and flow cytometry.
The Vinculin (E1E9V) antibody (#13901S) is a rabbit recombinant monoclonal antibody validated for WB, IHC, and FC in several human cell types, with WB of cell extracts shown on the CST website, and has 803 citations.
The LAMTOR4 (D4P60) antibody (#13140S) is validated for IF, IHC, IP, and WB in human samples with WB of cell extracts and IF shown on the CST website. It has 21 citations with use by both WB and IF, IHC.
The HRS (D7T5N) antibody (#15087S) is validated for WB, IP, and IF/ICC in human samples, with WB of cell extracts from several cell lines, IP, and IF of untreated, EGF treated, or wortmannin treated cells on the CST website. It has 29 citations with use of the antibody by WB and IF/ICC.
The TOM20 (D8T4N) antibody (#42406S) is validated for WB, IF, IHC, and IP in human, samples, with WB and IF of high and low expressing cell types shown on the CST website, and has 488 citations.
The ATG5 antibody (#2630S) is validated for WB and IP in human samples, with WB of cell extracts shown on the CST website, and has 391 citations.
The ATG13 (E1Y9V) antibody (#13468S) is validated for WB, IP, and IF in human samples, with IF of untreated or nutrient starved cells, IPs from MCF7 cell extracts, or WB of cell extracts from several cell lines shown on the CST website, and has 89 citations.
Santa Cruz Biotechnology: The LAMP2 (H4B4) antibody (#sc-18822) is validated by WB, and IF, IHC(P), and FC on the SCBT website, with IF images in HeLa cells and WB of cell extracts from several mammalian cell types shown on the SCBT website, and has 39 citations.
The CHMP1A (B-5) antibody (#sc-271617) validated WB, IP, IF, IHC(P), and ELISA, with WB of cell extracts shown on the CST website, and has 6 citations.
Abcam: The TFG (EPR8766) antibody (#ab156866) is validated for WB, IF/ICC, and IHC-P in human samples. IF of MCF-7 and HeLa cells, and WB of cell lysates from several human cell types, is shown on the Abcam website, and this antibody has 8 citations.
The NPC1 antibody (#ab36983) is validated by WB, IF, IP, and electron microscopy, and has 29 citations.
BD Biosciences: The SEC31A (Clone 32) antibody (#612350) is validated by WB of cell extracts and IF on the BD Biosciences website.
Proteintech: The SEC31A antibody (#17913-1-AP) validated for WB, IF/ICC, and IP, with WB of cell extracts from several human cell lines, and IF of HeLa cells shown on the Proteintech website, and has 13 citations.
The TSG101 (2B7G8) antibody (#67381-Ig) is a mouse monoclonal antibody validated for WB, IHC, IF/ICC, FC (intracellular), and IP, and has 69 citations. WB of cell lysates from several human cell lines is shown on the Proteintech website.
The ALIX (1H9D9) antibody (#67715-Ig) is validated for WB, IHC, and IF/ICC, with WB of cell extracts from various human cell lines as well as disappearance of the band in shRNA knockdown shown on the Proteintech website, and has 19 citations.
The PDCD6/ALG2 antibody (#12303-1-AP) is validated for WB, IP, IHC, and IF/ICC, with WB of cell lysates from several human cell lines shown on the Proteintech website, and has 23 citations.

Novus Biologicals: The TFG (TFG-03) antibody (#NBP2-62212) is validated by WB in Jurkat cell lysate and by WB of clonal Halo-TFG tagged cells, and is cited by 6 publications.

Eukaryotic cell lines

Policy information about cell lines and Sex and Gender in Research

Cell line source(s)	K562, U2OS, 293T, HeLa, RPE-1 cells from the UC Berkeley Cell Culture Facility (RRID SCR_017924)<br>i3 iPSC cells from Michael Ward lab (NIH).<br>WT and R106C mutant TFG patient fibroblast cells from Eleonora Lamantea (Fondazione IRCCS Instituto Neurologoico Carlo Besta).
Authentication	K562, U2OS, 293T, HeLa, and RPE-1 cells were authenticated by the UC Berkeley cell culture facility using short tandem repeat analysis using the GenePrint 10 System from Promega.<br><br>i3 iPSCs from Michael Ward were authenticated as described in published work (PMID 29924488), by genomic DNA extraction and PCR to confirm AAVS1 insertion of NGN2 and sequencing.<br><br>WT and R106C mutant TFG patient fibroblasts that we received were authenticated by whole exome sequencing as previously described (PMID 29971521)
Mycoplasma contamination	Cell lines were routinely tested by PCR based detection to confirm negative mycoplasma contamination.
Commonly misidentified lines<br>(See ICLAC register)	No commonly misidentified cell lines were used.

Plants

Seed stocks	N/A
Novel plant genotypes	N/A
Authentication	N/A
