# Supplementary material for: LASER couples damage sensing to ESCRT assembly for lysosome repair
Source: Nature. 2026 Jun 3;656(8126):216–26. doi: 10.1038/s41586-026-10604-6 (PMC13442042; doi:10.1038/s41586-026-10604-6)
Supplement: Supplementary file 3 [file 41586_2026_10604_MOESM4_ESM.pdf]

nature portfolio

Peer Review File

LASER couples damage sensing to ESCRT assembly for lysosome repair

Corresponding Author: Dr Roberto Zoncu

Any redactions in this file are there to maintain patient confidentiality, the confidentiality of unpublished data, or to remove third-party material.

This file contains all reviewer reports in order by version, followed by all author rebuttals in order by version.

Version 0:

Reviewer comments:

Referee #1

(Remarks to the Author)
The ESCRT complex plays important roles in fast lysosome repair. This study by Goul et al. identifies a lysosome damage sensor that recruits the ESCRT complex for membrane repair. Through genome-wide CRISPRi screens, the authors identify Trk-fused gene (TFG) as an ESCRT-associated factor that supports cell survival during lysosome damage. Knockdown of TFG significantly reduced the recruitment of ESCRT subunits to damaged lysosomes. TFG constitutively associates with ERES membranes, which the authors show to be reduced upon lysosome damage by LLOME. Regarding the mechanism of TFG recruitment, the authors show that an LIR motif of TFG is essential for its lysosomal recruitment following LLOME treatment. The authors further identify a PSAP motif within TFG that mediates interaction with TSG101 and thus ESCRT recruitment. Finally, the authors show that oligomerization deficient mutants of TFG fail to recruit ESCRT with defects in lysosome repair. Overall, this is an impressive study identifying TFG as an important factor in ESCRT recruitment to damaged lysosomes. I believe the comments below will further strengthen the paper.

1. ALIX is known to act in parallel with TSG101 for ESCRT-III recruitment. It is therefore difficult to envision how the TFG-TSG101 interaction could affect ALIX recruitment. The defects of ALIX recruitment in shTFG cells are marginal if any. Previous data showed that TSG101 knockdown delays but not abolishes ESCRT recruitment (PMID: 29622626, Fig. S7). It would be important to assess if TFG depletion abolishes ESCRT recruitment even 2 h after LLOME.
2. The upstream factor recruiting ESCRT to damaged lysosomes is often described as ALG-2 in literature, which is somewhat connected to TFG. ALG-2 was previously shown to promote TFG localization to ERES for polymerization (PMID: 27813252). What is the relationship between TFG-mediated and ALG-2 mediated ESCRT recruitment? Are they parallel or independent? Is the ALG-2-binding motif of TFG required for its function in ESCRT recruitment and lysosome repair? Previous data from Noboru Mizashima's group showed that ALG-2 knockdown did not affect ESCRT recruitment to damaged lysosome (PMID: 35274304). If TFG, rather than ALG-2, is essential, the authors should make it clear using TFG and ALG-2 KD/KO cells.
3. The repair defects of the PSAP-AAAA mutant are not shown. If the defect of this mutant is TSG101 recruitment, the mutant itself should still be recruited to lysosomes, which was not shown. Does TSG101 knockdown affect TFG recruitment?
4. The name "LASER" suggests that this is an LC3-specific complex. Is there evidence to support TFG recruitment by LC3 rather than GABARAP proteins? Testing TFG recruitment in ATG5, ATG7 and ATG8 knockout cells are recommended to validate the requirements of LC3/GABARAPs. It seems the authors did not show a repair defect of the TFG-LIR2 mutant. Functionally, does TFG depletion cause a level of repair defect similar to the depletion of ESCRT or ATG5?
5. Does TFG colocalize with LC3 and TSG101 on damaged lysosomes? If the recruitment is through direct binding, these proteins would be highly colocalized. Are TFG oligomerization-deficient mutants recruited to lysosomes?

Minor
6. The paper from Yaowen Wu's group is cited (ref 17), but it is not introduced that the main finding of that paper is a role of CASM/ATG8-ylation in ESCRT recruitment, which is highly relevant to the current study.
7. It is not defined what the "maximum" puncta mean in image quantifications. Wouldn't the average number/intensities of puncta be more appropriate? Error bars not defined, n numbers not described in legends; some graphs show a few dots per condition, does each dot represent one cell or the mean of one experiment?

8. Is CASM sufficient for the recruitment of TFG and ESCRT? Does LMP-independent CASM (eg., PMID 34597140) recruit TFG and ESCRT? Note that while LMP triggers CASM, the activation of CASM does not necessarily indicate LMP. It may be therefore not accurate to state CASM as a unifying marker for lysosome damage.
9. The high basal levels of galectin 3-mCherry puncta could be misleading, since galectin 3-mCherry is degraded in lysosomes, leaving behind a stable mCherry signal. An alternative to galectin 3-mCherry would provide a cleaner background.
10. Knockdown efficiency is not shown for ATG5 and ATG13.
11. It seems TFG is not essential for ESCRT-III assembly in U2OS cells, as all quantification data from U2OS cells did not show clear defects of LLOME-triggered ESCRT puncta formation. For example, in ED Fig 5F and 5P, the major difference is the basal puncta, but in each tested group the amounts of new puncta triggered by LLOME are the same. Similarly, marginal difference is observed in Fig 3J. If the quantification is reliable, the authors might want to discuss that in certain cell types, there are likely additional factors in parallel of TFG that mediate ESCRT recruitment. Is it possible that ALIX is expressed in high levels in U2OS which compensates for the loss of the TFG-TSG101 axis?
12. Any insights/explanations why TFG oligomers at ERES do not recruit ESCRT and how the damage of lysosomes drives TFG disassociation from ERES?

(Remarks on code availability)
The code provides a README file with enough instructions for installing and running, and the generation of the network part can run without errors. Some process in Fig. 1C (e.g., annotate genes with evidence, etc) is not provided, but is predictably repeatable.

Referee #2

(Remarks to the Author)
In this manuscript Goul and co-workers have used a genome-wide CRISPR screen with NPC1 KO cells to identify proteins that trigger recruitment of ESCRT proteins to damaged lysosome membranes in order to mediate their repair. The ER exit site protein TFG, associated with several neurological disorders, was identified as a major hit in the screen. Immunofluorescence microscopy showed that TFG mainly co-localized with the ER exit site marker SEC16 in untreated cells, whereas a major fraction was detected on LAMP2-positive lysosomes within 5 minutes of incubation with the lysosome-damaging agent LLOMe. The authors found that recruitment of TFG to damaged lysosomes depends on conjugation of ATG8 proteins to single membranes (CASM), and they identified a LIR sequence in TGF, LIR2, which abolished recruitment when mutated. The authors propose that TFG and ATG8 family proteins assemble into a complex, LASER, which promotes damage-induced ESCRT polymerization and membrane repair. The authors obtained biochemical evidence that TFG binds to the UEV domain of the ESCRT-I component TSG101 via a PSAP motif, a motif previously found to mediate interactions of ESCRT-0 and viral Gag proteins with TSG101. Mutation of the PB1 and CC domains of TFG, known to be involved in oligomerization, caused reduced binding of TFG to TSG101 and inhibited recruitment of ESCRT-III to damaged endosomes, suggesting that oligomers of TFG mediate ESCRT recruitment by avidity effects. The HSP-associated R106C mutation of TFG showed strongly reduced binding to the UEV domain of TSG101 in vitro, and patient-derived fibroblasts homozygous for this mutation showed decreased ESCRT-II recruitment, increased deacidification and increased Galectin-3 recruitment in response to LLOMe-induced lysosome damage. This suggests that the neuropathological effects of the R106C mutation may be linked to compromised lysosome integrity.

The topic of lysosomal membrane repair is very timely, and the manuscript makes a convincing case that the LASER complex plays an important role in this response. Novel molecular interactions are identified and characterized, conclusions are in general well supported by data, and quantifications are adequate. The question is whether there is a sufficient conceptual advance here for publication in Nature (see specific comment 1), but there is no doubt that the present manuscript represents a high-quality and high-impact study.

Specific comments:

1. Previous studies have shown that ESCRTs are required for repair of damaged lysosome membranes, and that the ESCRT-I subunit TSG101 and the ESCRT adaptor ALIX are involved in ESCRT-III recruitment (refs. 8,9). It has also been shown that CASM of the membrane of the damaged lysosome is required for ESCRT-mediated lysosome repair (Corkery et al., EMBO Rep. 2023; Ogura et al., EMBO Rep. 2023), and ALIX has been identified as a molecular link between ATG8 proteins and ESCRTs (Ogura et al., EMBO Rep. 2023). One may therefore argue that the concept of cooperation between ESCRTs and the ATG8 conjugation machinery in lysosomal membrane repair has already been established. The new and interesting contribution provided by this manuscript is another (and presumably more important) link between ATG8 proteins and ESCRTs, namely LASER. It may be worth discussing that depletion of ALIX is essentially without effect of ESCRT-III recruitment, whereas depletion of TSG101 causes a profound but incomplete effect, and that co-depletion of TSG101 and ALIX results in a complete inhibition of ESCRT-III recruitment (refs. 8,9). This can now be explained by the present data when reconciled with those of Ogura et al.

2. Why were the cells challenged for 8 days with LLOMe during CRISPRi screens? LLOMe activity typically declines within hours, so the rationale for extended treatment windows (days) needs justification. In Figure 1A it is stated "2 days drug treatment." The discrepancy should be clarified.

3. The assembly of the LASER complex on damaged lysosomes via ATG8-LIR interactions is convincingly shown, but the mechanisms remain incompletely characterized. The mechanisms that trigger lipid conjugation and association of ATG8

proteins on damaged lysosomes have been characterized by previous studies, but two issues remain unexplained: What is the signal for dissociation of TFG from ER exit sites in response to lysosome damage, and why does TSG101 fail to be recruited to TFG at ER exit sites? The authors speculate that binding of TFG to SEC16 might prevent its binding to TSG101, but they do not provide any evidence for this.

4. Since ER–lysosome contacts increase upon lysosomal damage (as reported previously), is TFG recruitment linked to enrichment of ERES at damaged lysosomes? This is hinted at in Extended Figure 8, but the authors should clarify the mechanism.

5. It has been shown previously that ALG-2 acts as a Ca2+-sensitive adaptor to concentrate and polymerize TFG at ERES (Kanadome et al., FEBS J. 2017. Do the authors find ALG2 important for TFG translocation to lysosomes? Additional studies manipulating ALG2 levels (KO or transient knockdown) should be carried out to clarify this further. Related to this, what happens to TFG recruitment/localization in the presence or absence of Ca2+?

6. How does TFG loss affect cholesterol levels? Could the observed phenotype upon lysosome damage be lipid-driven?

7. Can the authors insert the LIR2 domain from TFG to other ERES proteins such as SEC13, SEC31A or SEC16 and investigate whether those proteins exhibit the same behavior as TFG upon lysosome damage?

8. Among the sensitizing hits in Extended Data Figure 2 there are several ESCRT proteins annotated as direct hits in the screen, and the figure also includes “coessential genes” from DepMap. The latter leads to some confusion since the network annotated as “ESCRT-I/II complex” contains the ESCRT-0 subunit HGS and the ESCRT-0 binding protein STAMBP. As the authors mention in the Introduction, ESCRT-0 is not involved in recruitment of the other ESCRTs to damaged lysosomes, so this raises questions about the relevance of including the DepMap data in the figure.

9. Also related to Extended Data Figure 2, the ESCRT-II components of the “ESCRT-I/II complex” in this figure are all from DepMap and therefore fall into the same category as HGS and STAMBP. In the absence of experimental data showing that ESCRT-II is required for lysosome repair (it may be recruited to damaged lysosomes as a passenger with ESCRT-I) the authors should be cautious with implicating ESCRT-II in the repair process.

10. Studies with patient-derived fibroblasts are inherently associated with difficulties in obtaining appropriate controls, and the results with these would be strengthened by rescue experiments with wild-type and mutant TFG.

11. Sometimes mutations of LIR motifs affect interactions with ATG8 proteins because of indirect effects on protein folding or stability. Can this be excluded in the case of TFG? A biochemical experiment showing direct interactions between TFG and an ATG8 protein would be informative.

12. In contrast to Extended Figure 4D-E, in Fig. 1E Galectin-3 staining at 90 min post-LLOMe appears unchanged in shLuc. This should be explained.

13. Fig. 1E–G -F/ Extended Fig. 4D: Why do LLOMe concentrations vary between experiments?

14. Fig. 2C,F: Fluorescence and EM images suggest that TFG might localize inside lysosomes. Could the authors speculate on possible mechanisms or interpretations?

15. Fig. 3H: These data should be shown in the Extended figures.

(Remarks on code availability)

Referee #3

(Remarks to the Author)
I co-reviewed this manuscript with one of the reviewers who provided the listed reports.

(Remarks on code availability)

Referee #4

(Remarks to the Author)
Lysosome repair has emerged as an important aspect of organelle quality control and has been implicated in multiple human diseases, including neurodegeneration. Over the past decades, several factors such as lipid transfer proteins, ESCRTs, and CASM have been identified as contributing to this process. However, the early steps that connect the sensing of lysosomal damage to the recruitment of repair machinery, such as ESCRTs, remain poorly understood.
The senior author’s group has been at the forefront of lysosome biology, publishing a series of impactful studies in recent years. In the present work, they employ an elegant genetic screening approach that identifies a novel factor, TFG, as a key

player in lysosome repair. Through comprehensive analyses including live-cell imaging and biochemical assays, the authors delineate a new bridging mechanism that links damage sensing to ESCRT recruitment. I find the discovery of this mechanism convincing and believe it represents a major advance in the lysosome biology field. Below, I provide several suggestions that may help further refine the mechanistic insights and improve data presentation.

Major Comments

1. Specificity of the TFG–ATG8 mechanism

The study shows that TFG recognizes ATG8 on damaged lysosomes, thereby facilitating ESCRT recruitment. However, ATG8 proteins are activated in multiple contexts, including autophagy. If TFG recruitment depends solely on its association with ATG8, one could envision that TFG may also act on autophagosomes or other ATG8-decorated membranes. Indeed, TFG has previously been reported to associate with LC3 to regulate autophagosome biogenesis (PMID: 33932238), and ESCRT subunits have been implicated in autophagosome closure (PMID: 30030437). It would therefore be important to clarify whether this mechanism is unique to lysosome repair or extends to other ATG8-dependent pathways.

In my view, lysosome repair is distinct from autophagy because it requires the recruitment of broader ESCRT components, whereas autophagy primarily involves ESCRT-III. Additional factors may favor TFG recruitment specifically to damaged lysosomes. These may include:

ER–lysosome contact sites, as supported by EM data in the current study and prior work (Tan et al., PMID: 36071159).

Calcium-regulated assembly, as TFG oligomerization is known to be modulated by ALG-2 (PMID: 27813252), a factor also implicated in lysosome repair.

To strengthen this point, I suggest testing:

a. Whether TFG recruitment to damaged lysosomes requires ER–lysosome contacts.

b. Whether ER exit sites (ERES) physically contact damaged lysosomes.

c. Whether calcium signaling and ALG-2 are involved for TFG recruitment. Notably, the enlargement of TFG puncta upon lysosomal damage (Figure S7C) supports a role for calcium-enhanced assembly.

d. As an additional experiment, testing whether TFG–ATG8 interactions contribute to autophagosome closure by recruiting ESCRTs could broaden the appeal of this work to autophagy researchers.

2. Patient fibroblast experiments (Figure 5)

The R106C TFG mutant is defective not only in lysosome repair but also in ER exit site function and autophagy (PMID: 33932238). These broader defects could indirectly contribute to the lysosomal phenotypes observed. I recommend either testing an additional TFG mutant or explicitly discussing these alternative possibilities in the manuscript.

3. TFG function in NPC1KO cells

Since TFG was identified in NPC1KO cells, it would be interesting to know whether its role is more critical in this background than in WT cells. Specifically, did the authors compare the severity of ESCRT recruitment defects and lysosomal damage in the absence of TFG between WT and NPC1KO cells?

4. Biochemical validation of TFG–ATG8 interaction

An in vitro pulldown experiment could validate the interaction between TFG and ATG8 proteins and clarify the requirement of the LIR2 motif. Notably, a previous study (PMID: 33932238) reported that LIR3 mediates this interaction, raising a potential inconsistency. It would be valuable for the authors to address this issue experimentally or in discussion.

Minor Comments and Suggestions for Presentation

1. Starting from Figure 1e, lysosomal damage and factor recruitment are visualized as Galectin-3 puncta or other foci. While lysosome specialists can interpret these as bona fide damage markers, general readers may confuse them with protein aggregates. Including co-localization with lysosomal markers early in the figures would improve clarity.

2. In Figures 1b and S1C,D, the y-axis is labeled “Rho,” but the legends describe p-values. This is confusing and should be corrected.

3. In Figure 1b, TFG should be highlighted for emphasis.

4. In Figures S1C,D, I suggest using both text color (black/blue) and spot color to highlight the identified genes for clarity.

5. Figure 5A: The ΔPB mutant shows puncta resembling liquid droplets at 5 and 2.5 μM TFG. The authors should clarify this phenotype.

6. In Figure 3, specify the exact amino acid substitutions in the LIR mutants for accessibility to broader readers.

(Remarks on code availability)

Version 1:

Reviewer comments:

Referee #1

(Remarks to the Author)

The authors fully addressed my previous comments. I support publication.

Although ESCRT has been known to play a key role in lysosomal repair since 2018, how ESCRT is recruited to damaged lysosomes has been an essential unanswered question in the field. The revised manuscript identifies TFG as a key mediator of ESCRT recruitment, providing a convincing mechanistic link between calcium-dependent noncanonical autophagy (also known as CASM, conjugation of ATG8 to single membrane) and ESCRT-mediated lysosomal repair.

(Remarks on code availability)

Referee #2

(Remarks to the Author)
The authors have successfully addressed most of the points I raised, and the revision has significantly improved the quality of the manuscript. Nevertheless, two major concerns remain:

1. The recent paper by Corkery et al. (ref. 26) shows that ESCRT recruitment to damaged lysosomes can occur even in the absence of CASM, although CASM makes recruitment more efficient. Thus, LASER only represents one out of several alternative mechanisms for ESCRT recruitment to damaged lysosomes, and two such mechanisms have already been described previously. This reduces the priority for publication in Nature.

2. The mechanisms for TFG recruitment to damaged lysosomes and for prevention of ESCRT recruitment to ER exit sites (my original comment no. 3) have still not been fully addressed. It makes sense that calcium release from damaged lysosomes is a trigger of LASER recruitment since it is known to induce CASM. It is also interesting that VAP proteins are involved, but the molecular mechanism between CASM, TFG release from ER exit sites and LASER recruitment to damaged lysosomes is still not resolved. Regarding the lack of ESCRT recruitment to ERES, the authors speculate that ERES lack cofactors that enable ESCRT recruitment to damaged lysosomes. They need to provide experimental data that support this hypothesis.

Minor comment:

Even though the authors now cite previous studies on CASM and ESCRT recruitment, they do not make full justice to these studies: "…the molecular links between lysosome-bound ATG8 proteins and ESCRT components remain incompletely understood", "We cannot rule out alternative, TFG-independent mechanisms for ESCRT recruitment." A more detailed description of the previous work is required, as is an integration of the LASER data with the data from previous studies.

(Remarks on code availability)

Referee #3

(Remarks to the Author)
I co-reviewed this manuscript with one of the reviewers who provided the listed reports.

(Remarks on code availability)

Referee #4

(Remarks to the Author)
The authors have carried out extensive experiments to address my questions, leading to a significant improvement in the quality of the work. I believe the manuscript is now ready for publication.

(Remarks on code availability)

Version 2:

Reviewer comments:

Referee #2

(Remarks to the Author)
The authors have successfully addressed the remaining points I raised.

(Remarks on code availability)

Referee #3

(Remarks to the Author)
I co-reviewed this manuscript with one of the reviewers who provided the listed reports.

(Remarks on code availability)

Open Access This Peer Review File is licensed under a Creative Commons Attribution 4.0 International License, which permits use, sharing, adaptation, distribution and reproduction in any medium or format, as long as you give appropriate credit to the original author(s) and the source, provide a link to the Creative Commons license, and indicate if changes were made.
In cases where reviewers are anonymous, credit should be given to 'Anonymous Referee' and the source.
The images or other third party material in this Peer Review File are included in the article's Creative Commons license, unless indicated otherwise in a credit line to the material. If material is not included in the article's Creative Commons license and your intended use is not permitted by statutory regulation or exceeds the permitted use, you will need to obtain permission directly from the copyright holder.
To view a copy of this license, visit https://creativecommons.org/licenses/by/4.0/

Referee #1 (Remarks to the Author):

The ESCRT complex plays important roles in fast lysosome repair. This study by Goul et al. identifies a lysosome damage sensor that recruits the ESCRT complex for membrane repair. Through genome-wide CRISPRi screens, the authors identify Trk-fused gene (TFG) as an ESCRT-associated factor that supports cell survival during lysosome damage. Knockdown of TFG significantly reduced the recruitment of ESCRT subunits to damaged lysosomes. TFG constitutively associates with ERES membranes, which the authors show to be reduced upon lysosome damage by LLOME. Regarding the mechanism of TFG recruitment, the authors show that an LIR motif of TFG is essential for its lysosomal recruitment following LLOME treatment. The authors further identify a PSAP motif within TFG that mediates interaction with TSG101 and thus ESCRT recruitment. Finally, the authors show that oligomerization deficient mutants of TFG fail to recruit ESCRT with defects in lysosome repair. Overall, this is an impressive study identifying TFG as an important factor in ESCRT recruitment to damaged lysosomes. I believe the comments below will further strengthen the paper.

We thank the Reviewer for acknowledging the significance of our findings.

1. ALIX is known to act in parallel with TSG101 for ESCRT-III recruitment. It is therefore difficult to envision how the TFG-TSG101 interaction could affect ALIX recruitment. The defects of ALIX recruitment in shTFG cells are marginal if any. Previous data showed that TSG101 knockdown delays but not abolishes ESCRT recruitment (PMID: 29622626, Fig. S7). It would be important to assess if TFG depletion abolishes ESCRT recruitment even 2 h after LLOME.

The key initial publications[1,2] showed that TSG101 and Alix synergize to recruit the ESCRT-III machinery to damaged lysosomes, but with TSG101 playing a more important role. In our hands, TFG loss phenocopies TSG101 loss, and TFG is required for TSG101 recruitment, placing them along the same pathway (**Reviewer Fig. 1A-1B; Extended Data Fig. 7A, 7H-7J; all references to figures in this document refer to the revised manuscript**). The requirement for Alix appears to be more context specific. In NPC1-KO, TFG ablation decreased Alix recruitment more strongly than in wild-type cells (**Extended Data Fig. 6C-6D, 9F-9G**). Moreover, unlike in wild-type cells, knocking down Alix in NPC1-KO cells strongly suppressed ESCRT-III recruitment (**Reviewer Fig. 1C-1G; Fig. 1G-1H; Extended Data Fig. 9A-9C**). These data indicate that TFG primarily acts upstream of TSG101 in the key pathway for ESCRT-III recruitment and that the relative role of Alix may be context specific.

In response to the reviewer's other point, the defects in ESCRT-III recruitment upon knocking down TFG closely mirrored loss of TSG101, even after approximately 2 h (**Reviewer Fig. 1H-1J; Extended Data Fig. 5F**).

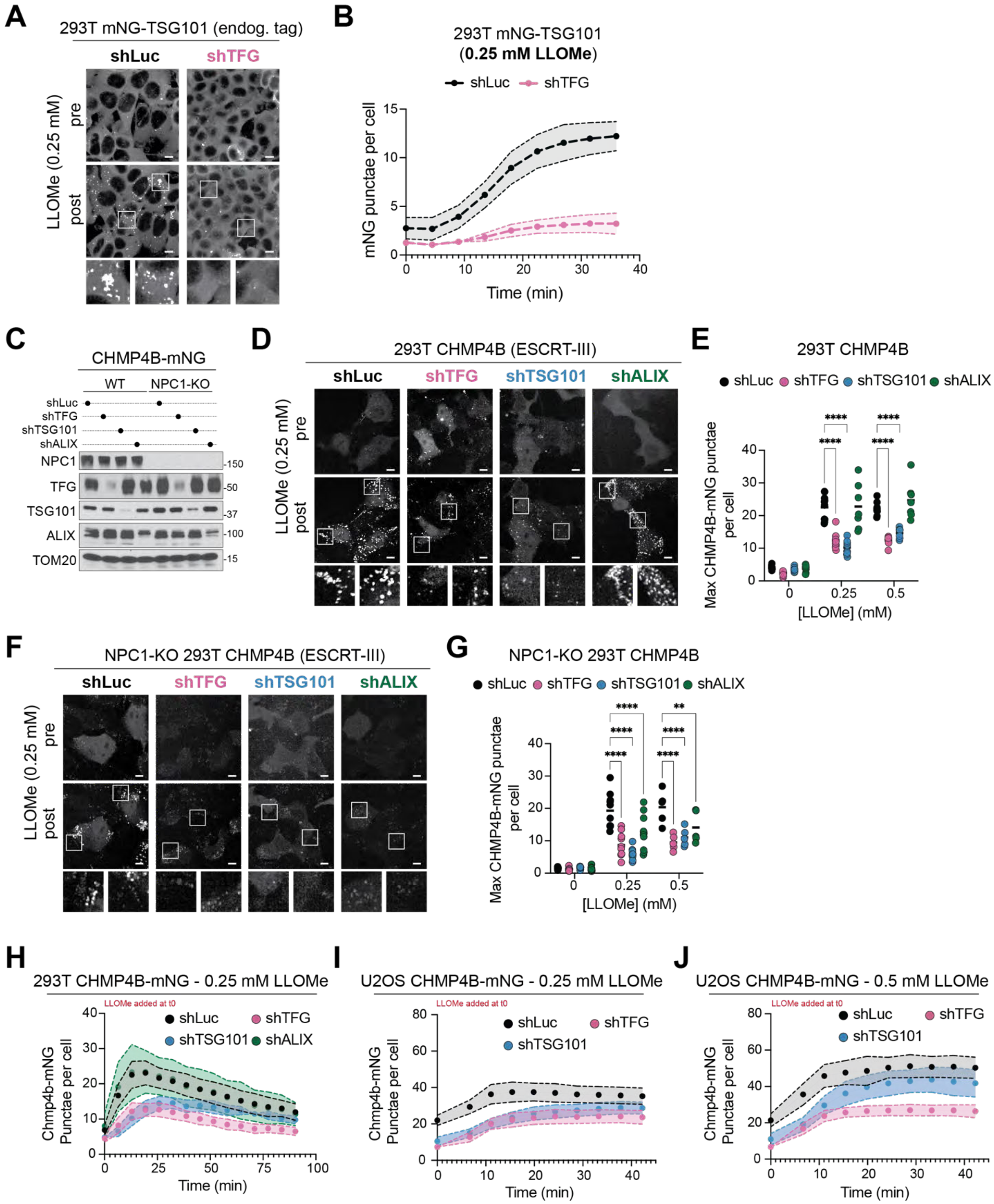

Reviewer Figure 1. TFG depletion phenocopies TSG101 loss and is obligate for ESCRT-III recruitment, whereas ALIX requirement is context-specific.

(**A, B**) Representative confocal images (**A**) and quantification (**B**) of maximum mNG-TSG101 puncta formed in 293T cells treated with DMSO (vehicle control) or 0.25 mM LLOMe following treatment with shRNA targeting TFG (pink) compared to Luc control (black). Images are shown for 25 minutes of treatment. N = 16 fields of view (FOVs). Scale bars, 10 µm.

(**C**) Western blot analysis of wild-type (WT) or NPC1 knockout (KO) CHMP4B-mNG expressing 293T cells following knockdown with shRNAs targeting Luciferase (Luc) control, TFG, TSG101, or ALIX.
(**D, E**) Representative confocal images (**D**) and quantification **(E)** of maximum CHMP4B-mNG puncta formed in 293T cells treated with DMSO (vehicle control), 0.25 mM, or 0.5 mM LLOMe following treatment with shRNA targeting TFG (pink), TSG101 (blue), or ALIX (green), compared to Luc control (black). Images are shown for 5 minutes of treatment. N = 8 FOVs. Scale bars, 10 µm.
(**F, G**) Representative confocal images **(F)** and quantification **(G)** of maximum CHMP4B-mNG puncta formed in 293T NPC1 KO cells treated with DMSO (vehicle control), 0.25 mM, or 0.5 mM LLOMe following treatment with shRNA targeting TFG (pink), TSG101 (blue), or ALIX (green), compared to Luc control (black). Images are shown for 5 minutes of treatment. N = 9 FOVs. Scale bars, 10 µm.
(**H**) CHMP4B-mNeonGreen puncta formation over time in HEK-293T transduced with shRNAs targeting Luc control, TFG, ALIX, or TSG101 and treated with 0.25 mM LLOMe. N = 8 FOVs.
(**I, J**) CHMP4B-mNeonGreen puncta formation over time in U2OS transduced with shRNAs targeting Luc control, TFG, or TSG101 and treated with 0.25 mM (**I**) or 0.5 mM **(J)** LLOMe. N = 12 FOVs.
Comparison of groups carried out by two-way ANOVA (**E, G**). Error bars represent mean +/- SD. **** p<0.001, **p<0.01.

2. The upstream factor recruiting ESCRT to damaged lysosomes is often described as ALG-2 in literature, which is somewhat connected to TFG. ALG-2 was previously shown to promote TFG localization to ERES for polymerization (PMID: 27813252). What is the relationship between TFG-mediated and ALG-2 mediated ESCRT recruitment? Are they parallel or independent? Is the ALG-2-binding motif of TFG required for its function in ESCRT recruitment and lysosome repair? Previous data from Noboru Mizashima's group showed that ALG-2 knockdown did not affect ESCRT recruitment to damaged lysosome (PMID: 35274304). If TFG, rather than ALG-2, is essential, the authors should make it clear using TFG and ALG-2 KD/KO cells.

We performed the suggested experiment, and our new data agree with the Mizushima lab's work[3]. When we knock down ALG2 in both 293T and U2OS cells, we see no defect in ESCRT-III recruitment upon LLOMe treatment (**Reviewer Fig. 2A-2F; Extended Data Fig. 14D-14I**). Moreover, TFG is also recruited to LLOMe-damaged lysosomes regardless of whether ALG2 is expressed or not (**Reviewer Fig. 2G-2I; Extended Data Fig. 14A-14C**). Finally, ALG2 was still recruited to damaged lysosomes in TFG-depleted cells (**Reviewer Fig. 2J**). Together, these data further indicate that the TFG-TSG101 pathway is dominant for ESCRT recruitment and that ALG2 is largely independent.

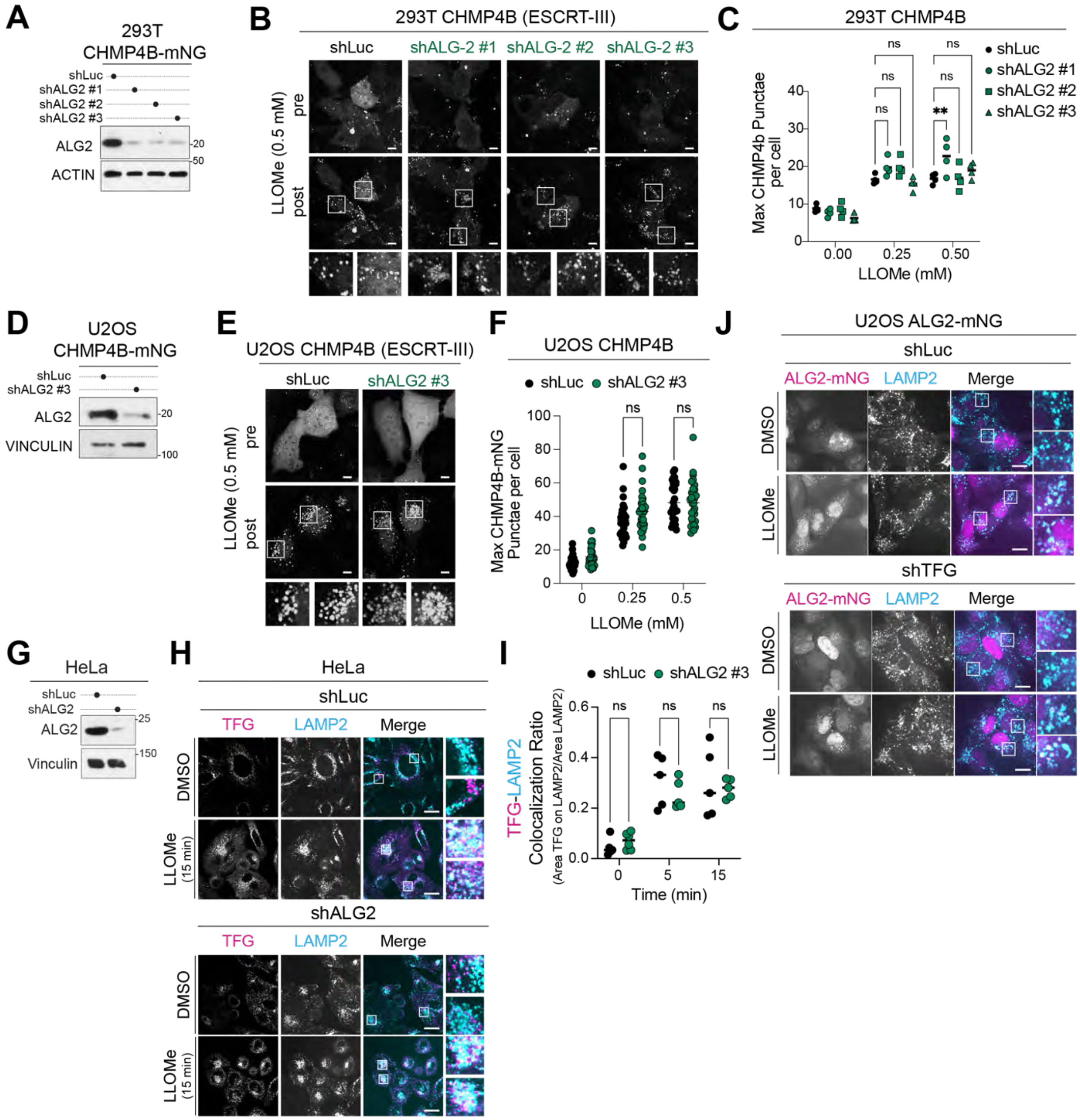

Reviewer Figure 2. TFG-TSG101 is obligate for ESCRT recruitment, whereas ALG2 is largely dispensable and functions in a TFG-independent pathway.

(**A**) Western blot analysis of wild-type (WT) CHMP4B-mNeonGreen (mNG) expressing 293T cells following knockdown with shRNAs targeting Luciferase (Luc) control or ALG2.

(**B, C**) Representative confocal images (**B**) and quantification (**C**) of maximum CHMP4B-mNG puncta formed in 293T cells treated with DMSO (vehicle control), 0.25 mM, or 0.5 mM LLOMe following treatment with shRNA targeting ALG2 (green) compared to Luc control (black). Images are shown for 15 minutes of treatment. N = 4 FOVs. Scale bars, 10 µm.

(**D**) Western blot analysis of wild-type (WT) CHMP4B-mNeonGreen (mNG) expressing U2OS cells following knockdown with shRNAs targeting Luciferase (Luc) control or ALG2.

(**E, F**) Representative confocal images (**E**) and quantification (**F**) of maximum CHMP4B-mNG puncta formed in U2OS cells treated with DMSO (vehicle control), 0.25 mM, or 0.5 mM LLOMe following treatment with shRNA targeting ALG2 (green) compared to Luc control (black). Images are shown for 15 minutes of treatment. N = 15 FOVs. Scale bars, 10 µm.

(**G**) Western blot analysis of HeLa cells following knockdown with shRNAs targeting Luciferase (Luc) control or ALG2.

(**H, I**) Representative confocal images (**H**) of TFG and LAMP2 immunofluorescence staining and quantification (**I**) of colocalization ratio between TFG and LAMP2 in HeLa cells. Cells were transduced with shRNAs targeting ALG2 or Luc, followed by treatment with vehicle control (DMSO) or 0.5 mM LLOMe for 5 or 15 minutes. Images are shown for 15 minutes of treatment. N = 5 FOVs. Scale bars, 10 µm.
(**J**) Representative confocal images of ALG2-mNeonGreen (mNG) expressing U2OS cells with LAMP2 immunofluorescence staining following treatment with 0.5 mM LLOMe and knockdown with shRNAs targeting Luc or TFG. Scale bars, 10 µm.
Comparison of groups carried out by two-way ANOVA (**C, F, I**). **p<0.01, ns=not significant.

3. The repair defects of the PSAP-AAAA mutant are not shown. If the defect of this mutant is TSG101 recruitment, the mutant itself should still be recruited to lysosomes, which was not shown. Does TSG101 knockdown affect TFG recruitment?

Thank you for the suggestions. We now show that PSAP>AAAA mutant has a pronounced repair defect, as shown by Dextran-FITC deacidification assay (**Reviewer Fig. 3A-3B; Fig. 4G-4H**). Also, as suggested by the reviewer, the PSAP>AAAA TFG mutant was recruited normally to damaged lysosomes, despite its inability to support ESCRT recruitment (**Reviewer Fig. 3C-3D; Extended Data Fig. 19J-19K**). Finally, knocking down TSG101 did not significantly hamper TFG localization to damaged lysosomes, again supporting a model where TFG is upstream of ESCRT-I recruitment (**Reviewer Fig. 3E-3G**).

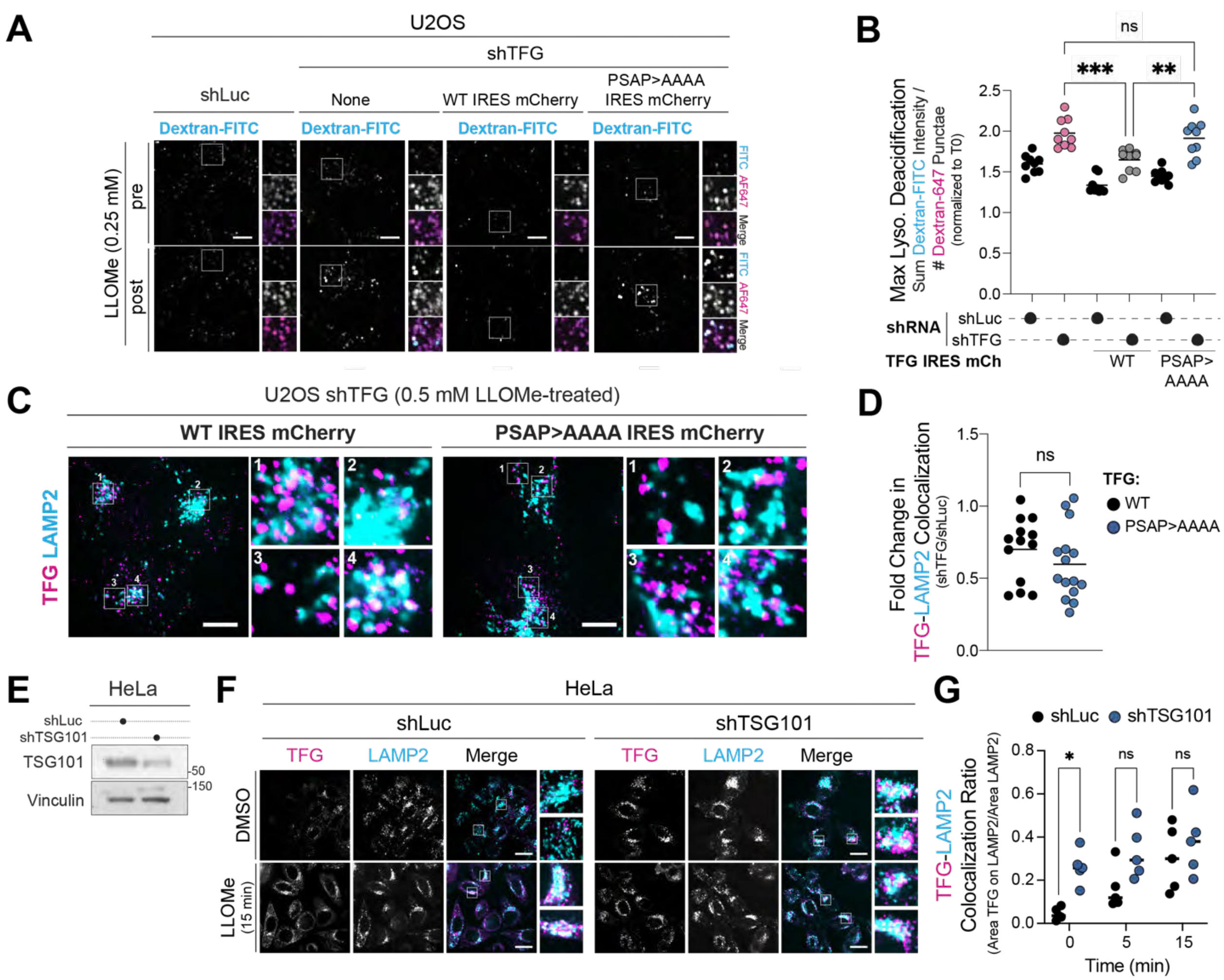

Reviewer Figure 3. TFG PSAP>AAAA translocates to damaged lysosomes but fails to promote their repair.
(**A, B**) Representative confocal images (**A**) and quantification (**B**) of total fluorescence intensity ratio between internalized dextran (FITC vs TMR) punctae following treatment with 0.25 mM LLOMe. U2OS cells were transduced with shRNAs targeting Luc or TFG and reconstituted with wild-type or PSAP mutant (PSAP>AAAA) TFG. N = 10 FOVs. Scale bars, 10 μm.
(**C, D**) Representative confocal images (**C**) of TFG and LAMP2 immunofluorescence staining and quantification (**D**) of colocalization ratio between TFG and LAMP2 in U2OS cells. Cells were transduced with shRNAs targeting TFG or Luc control and reconstituted with wild-type or PSAP>AAAA mutant TFG, followed by treatment with vehicle control (DMSO) or 0.5 mM LLOMe. Images are shown for 15 minutes of treatment. N = 14 FOVs. Scale bars, 5 μm.
(**E**) Western blot analysis of HeLa cells following knockdown with shRNAs targeting Luc control or TSG101.
(**F, G**) Representative confocal images (**F**) of TFG and LAMP2 immunofluorescence staining and quantification (**G**) of colocalization ratio between TFG and LAMP2 in HeLa cells. Cells were transduced with shRNAs targeting TSG101 or Luc, followed by treatment with DMSO or 0.5 mM LLOMe for 5 or 15 minutes. Images are shown for 15 minutes of treatment. N = 5 FOVs. Scale bars, 10 μm.
Comparison of groups carried out by unpaired t-test (**D**) or one-way ANOVA (**B, G**). ***p<0.001, **p<.01, *p<.05, ns=not significant.

4. The name "LASER" suggests that this is an LC3-specific complex. Is there evidence to support TFG recruitment by LC3 rather than GABARAP proteins? Testing TFG recruitment in ATG5, ATG7 and ATG8 knockout cells are recommended to validate the requirements of LC3/GABARAPs. It seems the authors did not show a repair defect of the TFG-LIR2 mutant. Functionally, does TFG depletion cause a level of repair defect similar to the depletion of ESCRT or ATG5?

Our peptide binding assays showed that TFG binds to both LC3 and GABARAP via its LIR2 (**Extended Data Fig. 18A-18D**). Based on this and on additional new data, we agree that the correct name for the pathway is LC3/GABARAP-Assisted Stimulator for ESCRT Recruitment. Additionally, we now confirm that TFG still gets recruited to damaged lysosomes upon loss of general autophagy regulator, ATG13, further validating the role of CASM in TFG recruitment (**Reviewer Fig. 4A-4C; Extended Data Fig. 16E-16G**). Finally, we now show that expressing the LIR2 mutant of TFG results in a repair defect, as shown by Dextran-FITC deacidification assay (**Reviewer Fig. 4D, 4E; Fig. 3H-3I**), and that ATG5 and TSG101 depletion leads to a repair defect as with TFG depletion (**Reviewer Fig. 4F, 4G; Extended Data Fig. 17I-17J**).

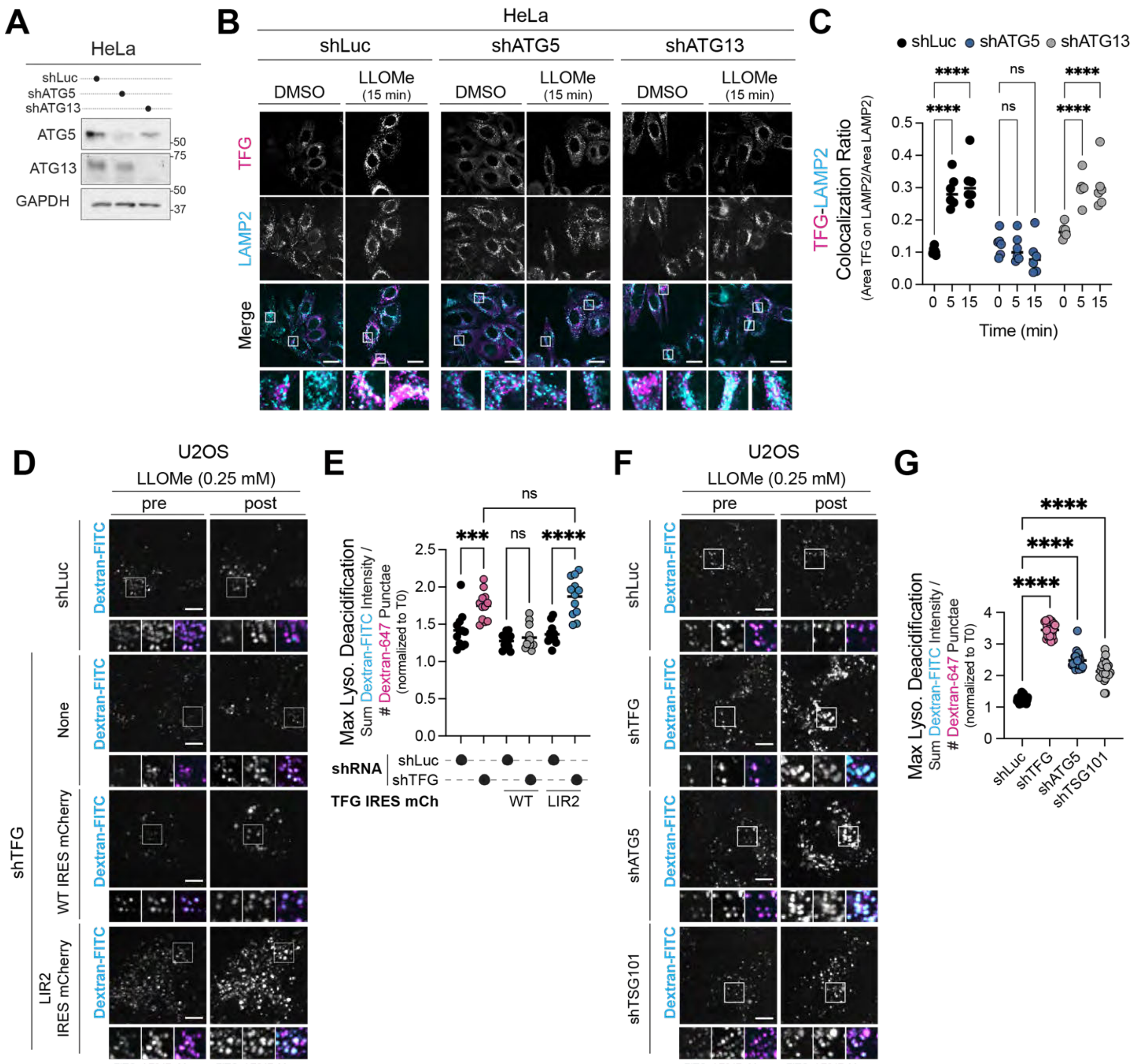

Reviewer Figure 4. TFG translocation to damaged lysosomes is ATG5-dependent but ATG13-independent, and an intact LIR2 is essential for lysosomal repair.

(**A**) Western blot analysis of wild-type (WT) HeLa cells following knockdown with shRNAs targeting Luc control, ATG5, or ATG13.

(**B, C**) Representative confocal images (**B**) of TFG and LAMP2 immunofluorescence staining and quantification (**C**) of colocalization ratio between TFG and LAMP2 in HeLa cells. Cells were transduced with shRNAs targeting ATG5, ATG13, or Luc, followed by treatment with DMSO (vehicle control) or 0.5 mM LLOMe for 5 or 15 minutes. Images are shown for 15 minutes of treatment. N = 6 FOVs. Scale bars, 10 µm.

(**D, E**) Representative confocal images (**D**) and quantification (**E**) of total fluorescence intensity ratio between internalized dextran (FITC vs TMR) punctae following treatment with 0.25 mM LLOMe. U2OS cells were transduced with shRNAs targeting Luc or TFG and reconstituted with wild-type or LIR2 mutant TFG. N = 12 FOVs. Scale bars, 10 µm.

(**F, G**) Representative confocal images (**F**) and quantification (**G**) of total fluorescence intensity ratio between internalized dextran (FITC vs TMR) punctae following treatment with 0.25 mM LLOMe. U2OS cells were transduced with shRNAs targeting Luc, TFG, ATG5, or TSG101. N = 24 FOVs. Scale bars, 10 µm.

Comparison of groups carried out by two-way ANOVA (**C, E, G**). ****p<0.0001, ***p<0.001, ns=not significant.

5. Does TFG colocalize with LC3 and TSG101 on damaged lysosomes? If the recruitment is through direct binding, these proteins would be highly colocalized. Are TFG oligomerization-deficient mutants recruited to lysosomes?

We now show that TFG highly colocalizes with LC3B, GABARAP and TSG101 on damaged lysosomes (**Reviewer Fig. 5A-5F; Extended Data Fig. 10E-10F, 15A-15D**). We also tested the ability of various oligomerization-deficient mutants of TFG to be recruited to damaged lysosomes. Interestingly, we find that all tested mutants (PB1-deleted, CC-deleted, and R106C) showed a normal pattern of lysosomal colocalization (**Reviewer Fig. 5G-5J; Extended Data Fig. 20E-20H**). These data suggest that oligomerization through these domains may primarily support binding to TSG101 rather than to ATG8 proteins. More mechanistic dissection will be required to elucidate the relationship between TFG's oligomerization and phase-separation mediating motifs (including the PQ-rich tail), and its various interactors.

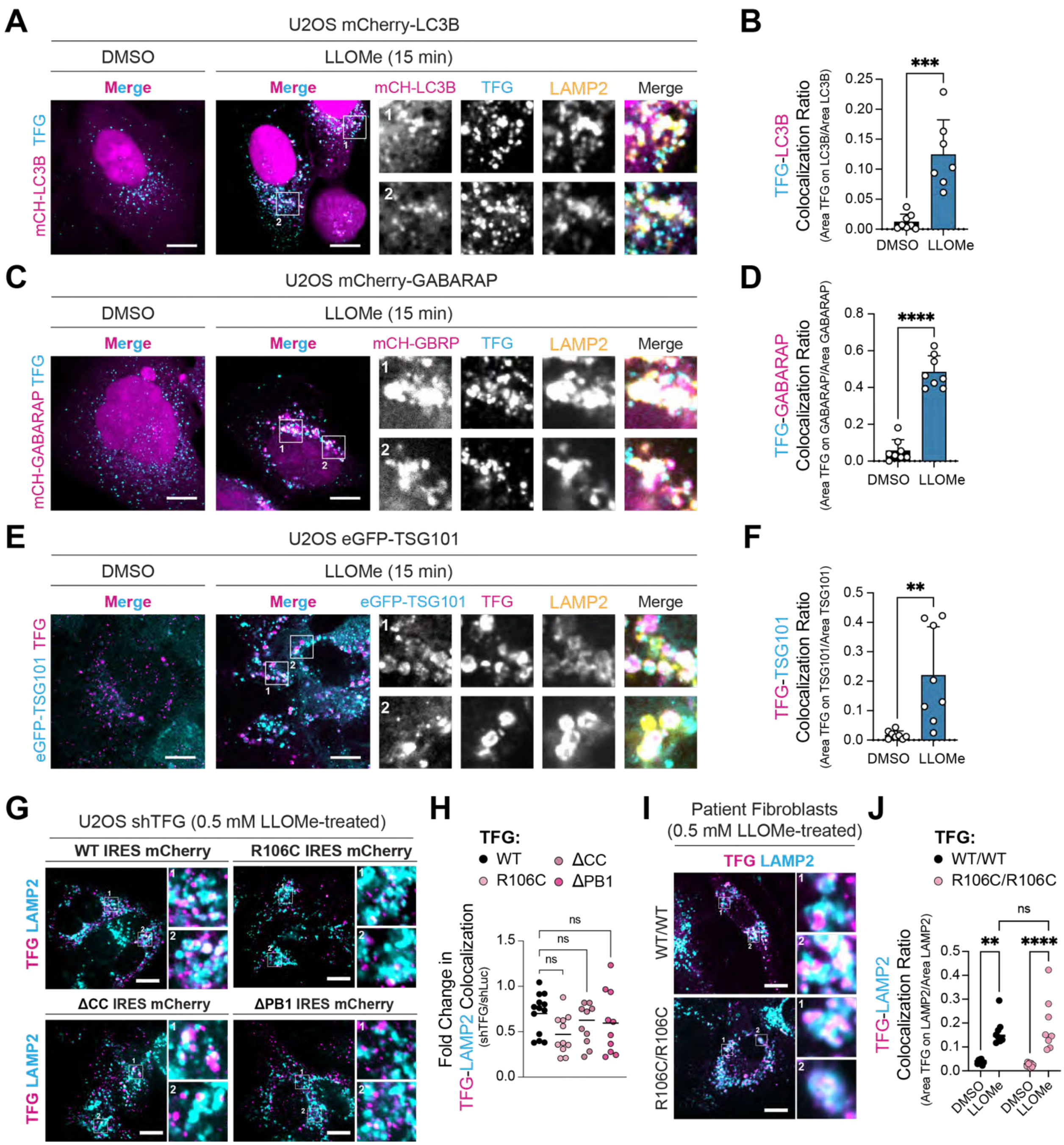

Reviewer Figure 5. TFG co-localizes with ATG8 proteins and TSG101 at damaged lysosomes.
(**A, B**) Representative confocal images (**A**) of TFG, mCherry-LC3B, and LAMP2 immunofluorescence staining and quantification (**B**) of colocalization ratio between TFG and mCherry-LC3B in U2OS cells expressing mCherry-LC3B. Cells were treated with DMSO or 0.5 mM LLOMe for 15 minutes. N = 7 FOVs. Scale bars, 5 µm.
(**C, D**) Representative confocal images (**C**) of TFG, mCherry-GABARAP, and LAMP2 immunofluorescence staining and quantification (**D**) of colocalization ratio between TFG and mCherry-GABARAP in U2OS cells expressing mCherry-GABARAP. Cells were treated with DMSO or 0.5 mM LLOMe for 15 minutes. N = 8 FOVs. Scale bars, 5 µm.
(**E, F**) Representative confocal images (**E**) of TFG, eGFP-TSG101, and LAMP2 immunofluorescence staining and quantification (**F**) of colocalization ratio between TFG and eGFP-TSG101 in U2OS cells expressing eGFP-TSG101. Cells were treated with DMSO or 0.5 mM LLOMe for 15 minutes. N = 8 FOVs. Scale bars, 5 µm.
(**G, H**) Representative confocal images (**G**) of TFG and LAMP2 immunofluorescence staining and quantification (**H**) of colocalization ratio between TFG and LAMP2 in U2OS cells transduced with hairpins targeting Luc control or TFG

reconstituted with wild-type TFG, R106C mutant TFG, or TFG lacking the PB1 or coiled-coil (CC) domains. Cells were treated with 0.5 mM LLOMe for 15 minutes. N = 10 FOVs. Scale bars, 5 µm.
(**I, J**) Representative confocal images (**I**) of TFG and LAMP2 immunofluorescence staining and quantification (**J**) of colocalization ratio between TFG and LAMP2 in $TFG^{R106C/R106C}$ or control ($TFG^{WT/WT}$) patient fibroblast cells treated with 0.5 mM LLOMe for 15 minutes. N = 8 FOVs. Scale bars, 5 µm.
Comparison of groups carried out by unpaired t-test (**B, D, F**) or one-way ANOVA (**H, J**). Error bars represent mean +/- SD. ****p<0.0001, ***p<0.001, **p<0.01, ns=not significant.

Minor

6. The paper from Yaowen Wu's group is cited (ref 17), but it is not introduced that the main finding of that paper is a role of CASM/ATG8-ylation in ESCRT recruitment, which is highly relevant to the current study.

We agree and now mention this work in the revised introduction.

7. It is not defined what the "maximum" puncta mean in image quantifications. Wouldn't the average number/intensities of puncta be more appropriate? Error bars not defined, n numbers not described in legends; some graphs show a few dots per condition, does each dot represent one cell or the mean of one experiment?

Here, "maximum" refers to the maximum number of puncta per cell observed following LLOMe treatment over a defined time course (up to 35-40 min) for each condition. We have also clarified the definition of the error bars (typically mean ± standard deviation) and specified what each data point represents in the corresponding figure legends of the revised manuscript (generally one field of view, comprising tens to hundreds of cells depending on imaging magnification).

8. Is CASM sufficient for the recruitment of TFG and ESCRT? Does LMP-independent CASM (eg., PMID 34597140) recruit TFG and ESCRT? Note that while LMP triggers CASM, the activation of CASM does not necessarily indicate LMP. It may be therefore not accurate to state CASM as a unifying marker for lysosome damage.

We agree that CASM is more universal than LMP and may indicate, more generally, lysosomal stress. Following the reviewer's suggestion, we stimulated the cells with the TRPML1 agonist, ML-SA5, which was previously shown to trigger lysosomal membrane ATG8ylation. We saw that, in fact, TRPML1 activation was sufficient to induce CASM and lysosomal TFG recruitment to a level comparable to LLOMe treatment, and that this response was abolished by calcium chelation by BAPTA-AM (**Reviewer Fig. 6A-6D; Fig. 2F-2G; Extended Data Fig. 15E-15F**). Strikingly, TRPML1 activation was also sufficient to induce ESCRT-III recruitment to (undamaged) lysosomes, a response also blocked by BAPTA-AM in accordance with previous reports[1] (**Reviewer Fig. 6E-6H; Extended Data Fig. 13A-13D**). Together, these data point to calcium induced LASER assembly as a key driver for ESCRT recruitment.

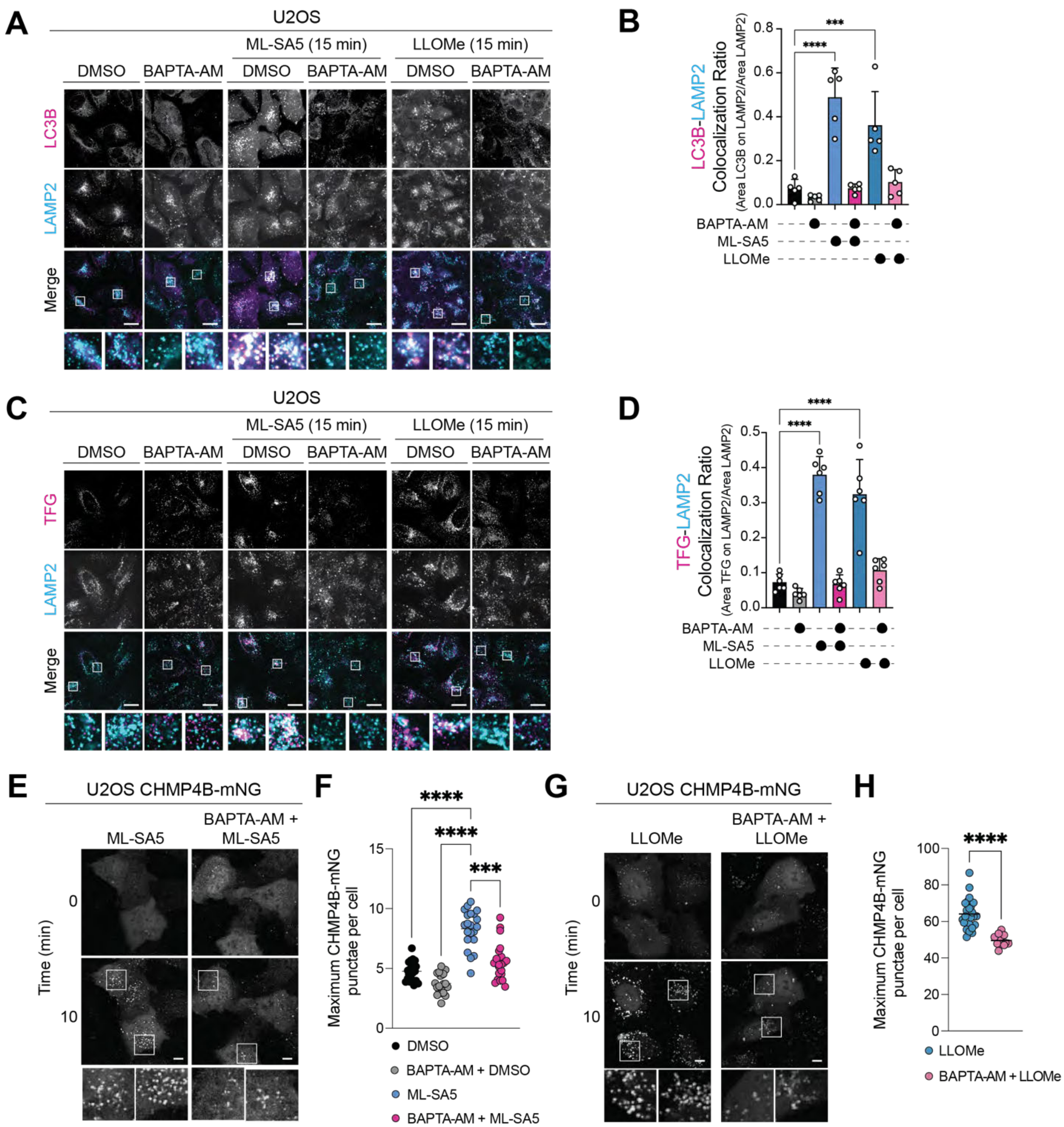

Reviewer Figure 6. Lysosomal calcium is necessary and sufficient for TFG recruitment and ESCRT assembly.
(**A, B**) Representative confocal images (**A**) of LC3B and LAMP2 immunofluorescence staining and quantification (**B**) of colocalization ratio between LC3B and LAMP2 in U2OS cells. Cells were treated with vehicle control (DMSO) or 20 µM BAPTA-AM, followed by treatment with either 4 µM MLSA5 or 0.5 mM LLOMe for 15 minutes. N = 5 FOVs. Scale bars, 10 µm.
(**C, D**) Representative confocal images (**C**) of TFG and LAMP2 immunofluorescence staining and quantification (**D**) of colocalization ratio between TFG and LAMP2 in U2OS cells. Cells were treated with vehicle control (DMSO) or 20 µM BAPTA-AM, followed by treatment with either 4 µM MLSA5 or 0.5 mM LLOMe for 15 minutes. N = 6 FOVs. Scale bars, 10 µm.
(**E, F**) Representative confocal images (**E**) and quantification (**F**) of maximum CHMP4B-mNeonGreen puncta in U2OS cells upon treatment with DMSO or 4 µM ML-SA5, with or without pre-treatment for 1 hour with 20 µM BAPTA-AM. Images are shown for 10 minutes of treatment. N = 21 FOVs. Scale bars, 10 µm.

(**G, H**) Representative confocal images (**G**) and quantification (**H**) of maximum CHMP4B-mNeonGreen puncta in U2OS cells upon treatment with LLOMe, with or without pre-treatment for 1 hour with 20 μM BAPTA-AM. Images are shown for 10 minutes of treatment. N = 24 FOVs. Scale bars, 10 μm.
Comparison of groups carried out by one-way ANOVA (**B, D, F**) or unpaired t-test (**H**). Error bars represent mean +/- SD. ****p<0.0001, ***p<0.001.

9. The high basal levels of galectin 3-mCherry puncta could be misleading, since galectin 3-mCherry is degraded in lysosomes, leaving behind a stable mCherry signal. An alternative to galectin 3-mCherry would provide a cleaner background.

We thank the reviewer for highlighting this concern. As an alternative to Gal3-mCherry overexpression to monitor lysosomal repair, we incubated cells with Dextran-FITC (pH sensitive) and Dextran-647 (pH insensitive). Upon treatment with LLOMe, lysosomes get deacidified and Dextran-FITC de-quenches leading to increased Dextran-FITC intensity per Dextran-647 puncta. Using this experimental paradigm, we found that TFG KD leads to increased lysosomal deacidification (i.e. damage) compared to Luc control (**Reviewer Fig. 4F, 4G; Extended Data Fig. 17I-17J**), consistent with our findings using the Gal3-mCh probe (**Fig. 1E, 1F; Extended Data Fig. 4C-4E**).

10. Knockdown efficiency is not shown for ATG5 and ATG13.

We have now included western blots for shATG5 and shATG13 KD efficiency (**Reviewer Fig. 4A; Extended Data Fig. 16E**).

11. It seems TFG is not essential for ESCRT-III assembly in U2OS cells, as all quantification data from U2OS cells did not show clear defects of LLOME-triggered ESCRT puncta formation. For example, in ED Fig 5F and 5P, the major difference is the basal puncta, but in each tested group the amounts of new puncta triggered by LLOME are the same. Similarly, marginal difference is observed in Fig 3J. If the quantification is reliable, the authors might want to discuss that in certain cell types, there are likely additional factors in parallel of TFG that mediate ESCRT recruitment. Is it possible that ALIX is expressed in high levels in U2OS which compensates for the loss of the TFG-TSG101 axis?

We appreciate the reviewers' point. Given the many ESCRT-interacting proteins on the endolysosomal surface, we cannot exclude additional ESCRT recruiting mechanisms, such as Alix, playing an accessory or compensatory role, and this could be indeed cell-type specific as, in our hands, it is more evident in U2OS than others (**Fig. 1G-1H; Extended Data Fig. 5G-5J**). Still, even in U2OS, the key observation is that TFG loss phenocopies TSG101 loss, placing them in the same pathway.

12. Any insights/explanations why TFG oligomers at ERES do not recruit ESCRT and how the damage of lysosomes drives TFG disassociation from ERES?

We provide three new pieces of data that start to delineate a model for TFG translocation from ERES to lysosomes during damage. The first is that blocking CASM by ATG5-KD not only blocks TFG association with LAMP2 but also prevents TFG dissociation from ERES marker, SEC31A (**Reviewer Fig. 7A, 7B**). The second is that co-depletion of VAPA/B, which are required for ER-lysosome contacts during damage-repair, also blocks TFG translocation to lysosomes (**Reviewer Fig. 7C-7E; Extended Data Fig. 12B-12D**). Combined, these results suggest that upon damage, ERES are brought into proximity to lysosomes via canonical, VAP-mediated, ER-lysosome contacts. In turn, this proximity may enable TFG to be captured by LC3B, providing the driving

force for TFG to separate from ERES. Further investigation is required to fully elucidate this model.

In terms of the identity of the most upstream signal that triggers TFG association with damaged lysosomes, our new data implicate calcium release from the lysosomal lumen. Specifically, we show that TRPML1 stimulation is sufficient to recruit TFG to lysosomes to a level similar to LLOMe stimulation, and that both TRPML1 and LLOMe dependent TFG recruitment are abolished by the calcium chelator BAPTA-AM (**Reviewer Fig. 6A-6D; Fig. 2F-2G**).

Finally, as to why ESCRTs (TSG101) do not bind to ERES, we point out that the membrane microenvironment of ERES is likely very different from the endolysosomal membrane. Differences in lipid composition or lack of specific cofactors, including perhaps ATG8 proteins themselves, could explain why ESCRT has not been observed at ERES under any conditions.

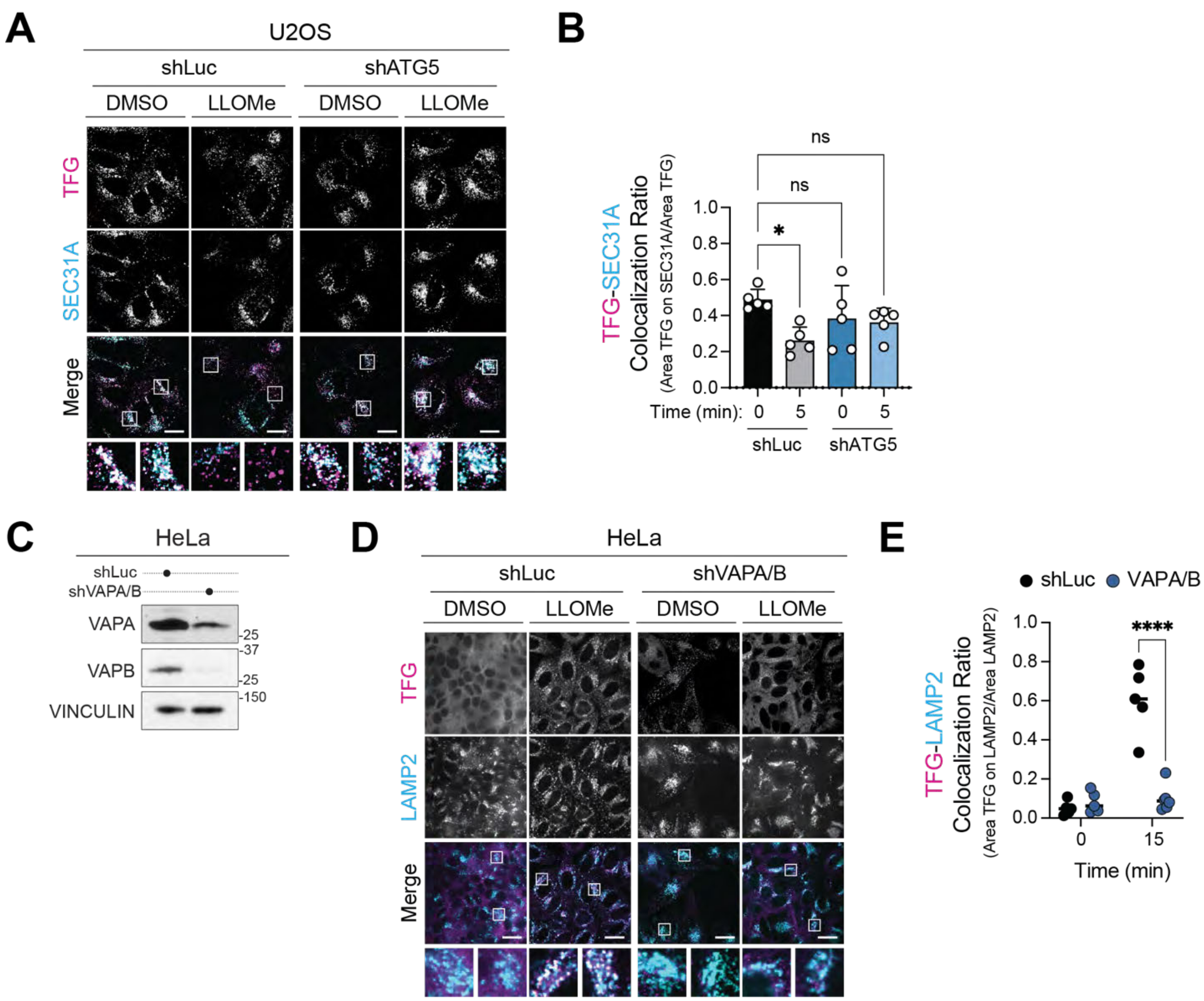

Reviewer Figure 7. CASM and VAPA/B are required for TFG release from ERES upon lysosomal damage.
(**A, B**) Representative confocal images of TFG and SEC31A immunofluorescence staining (**A**) and quantification (**B**) of the fraction of TFG colocalized with SEC31A in U2OS cells following treatment with shRNAs targeting Luc control or ATG5, followed by treatment with DMSO (vehicle control) or 0.5 mM LLOMe for 5 minutes. N = 5 FOVs. Scale bars, 10 µm.
(**C**) Western blot analysis of HeLa cells following knockdown with shRNAs targeting Luc or VAPA and VAPB.
(**D, E**) Representative confocal images of TFG and LAMP2 immunofluorescence staining (**D**) and quantification (**E**) of the fraction of LAMP2 colocalized with TFG in HeLa cells following treatment with shRNAs targeting Luc or VAPA and

VAPB followed by treatment with DMSO (vehicle control) or 0.5 mM LLOMe for 15 minutes. N = 5 FOVs. Scale bars, 10 μm.
Comparison of groups carried out by one-way ANOVA (**B, E**). Error bars represent mean +/- SD. ****p<0.0001, *p<0.05, ns=not significant.

Referee #1 (Remarks on code availability):
The code provides a README file with enough instructions for installing and running, and the generation of the network part can run without errors. Some process in Fig. 1C (e.g., annotate genes with evidence, etc) is not provided, but is predictably repeatable.

We are glad the reviewer could run our code; the annotation of genes with neurodegeneration mutations was added manually and we have now added this in the Methods.

Referee #2 (Remarks to the Author):

In this manuscript Goul and co-workers have used a genome-wide CRISPR screen with NPC1 KO cells to identify proteins that trigger recruitment of ESCRT proteins to damaged lysosome membranes in order to mediate their repair. The ER exit site protein TFG, associated with several neurological disorders, was identified as a major hit in the screen. Immunofluorescence microscopy showed that TFG mainly co-localized with the ER exit site marker SEC16 in untreated cells, whereas a major fraction was detected on LAMP2-positive lysosomes within 5 minutes of incubation with the lysosome-damaging agent LLOMe. The authors found that recruitment of TFG to damaged lysosomes depends on conjugation of ATG8 proteins to single membranes (CASM), and they identified a LIR sequence in TGF, LIR2, which abolished recruitment when mutated. The authors propose that TFG and ATG8 family proteins assemble into a complex, LASER, which promotes damage-induced ESCRT polymerization and membrane repair. The authors obtained biochemical evidence that TFG binds to the UEV domain of the ESCRT-I component TSG101 via a PSAP motif, a motif previously found to mediate interactions of ESCRT-0 and viral Gag proteins with TSG101. Mutation of the PB1 and CC domains of TFG, known to be involved in oligomerization, caused reduced binding of TFG to TSG101 and inhibited recruitment of ESCRT-III to damaged endosomes, suggesting that oligomers of TFG mediate ESCRT recruitment by avidity effects. The HSP-associated R106C mutation of TFG showed strongly reduced binding to the UEV domain of TSG101 in vitro, and patient-derived fibroblasts homozygous for this mutation showed decreased ESCRT-II recruitment, increased deacidification and increased Galectin-3 recruitment in response to LLOMe-induced lysosome damage. This suggests that the neuropathological effects of the R106C mutation may be linked to compromised lysosome integrity.

The topic of lysosomal membrane repair is very timely, and the manuscript makes a convincing case that the LASER complex plays an important role in this response. Novel molecular interactions are identified and characterized, conclusions are in general well supported by data, and quantifications are adequate. The question is whether there is a sufficient conceptual advance here for publication in Nature (see specific comment 1), but there is no doubt that the present manuscript represents a high-quality and high-impact study.

We thank the reviewer for acknowledging the significance and rigor of our study.

Specific comments:

1. Previous studies have shown that ESCRTs are required for repair of damaged lysosome membranes, and that the ESCRT-I subunit TSG101 and the ESCRT adaptor ALIX are involved in ESCRT-III recruitment (refs. 8,9). It has also been shown that CASM of the membrane of the damaged lysosome is required for ESCRT-mediated lysosome repair (Corkery et al., EMBO Rep. 2023; Ogura et al., EMBO Rep. 2023), and ALIX has been identified as a molecular link between ATG8 proteins and ESCRTs (Ogura et al., EMBO Rep. 2023). One may therefore argue that the concept of cooperation between ESCRTs and the ATG8 conjugation machinery in lysosomal membrane repair has already been established. The new and interesting contribution provided by this manuscript is another (and presumably more important) link between ATG8 proteins and ESCRTs, namely LASER. It may be worth discussing that depletion of ALIX is essentially without effect of ESCRT-III recruitment, whereas depletion of TSG101 causes a profound but incomplete effect, and that co-depletion of TSG101 and ALIX results in a complete inhibition of ESCRT-III recruitment (refs. 8,9). This can now be explained by the present data when reconciled with those of Ogura et al.

We agree with the reviewer that our identification of TFG in lysosomal membrane repair provides a key missing link between CASM and ESCRT recruitment. This motivates the renaming of ATG8-ylation plus TFG recruitment collectively as LASER. As the reviewer points out, it is worth further emphasizing that LASER is more critical to early ESCRT assembly than previously proposed ALIX and ALG2. This is supported by our new data which shows that while TFG loss phenocopies TSG101 loss, both ALIX and ALG2 are largely dispensable for ESCRT-III recruitment to damaged lysosomes (**Reviewer Fig. 8A-8I; Fig. 1G-1H; Extended Data Fig. 14D-14I**). Interestingly, unlike in wild-type cells, in NPC1-KO cells ALIX knockdown impaired ESCRT recruitment (**Reviewer Fig. 8J-8K; Extended Data Fig. 9A-9C**), suggesting a context- and extent-of-damage specific role for this protein. We have updated the Discussion to convey these important differences and the prominence of TFG versus previously proposed mechanisms.

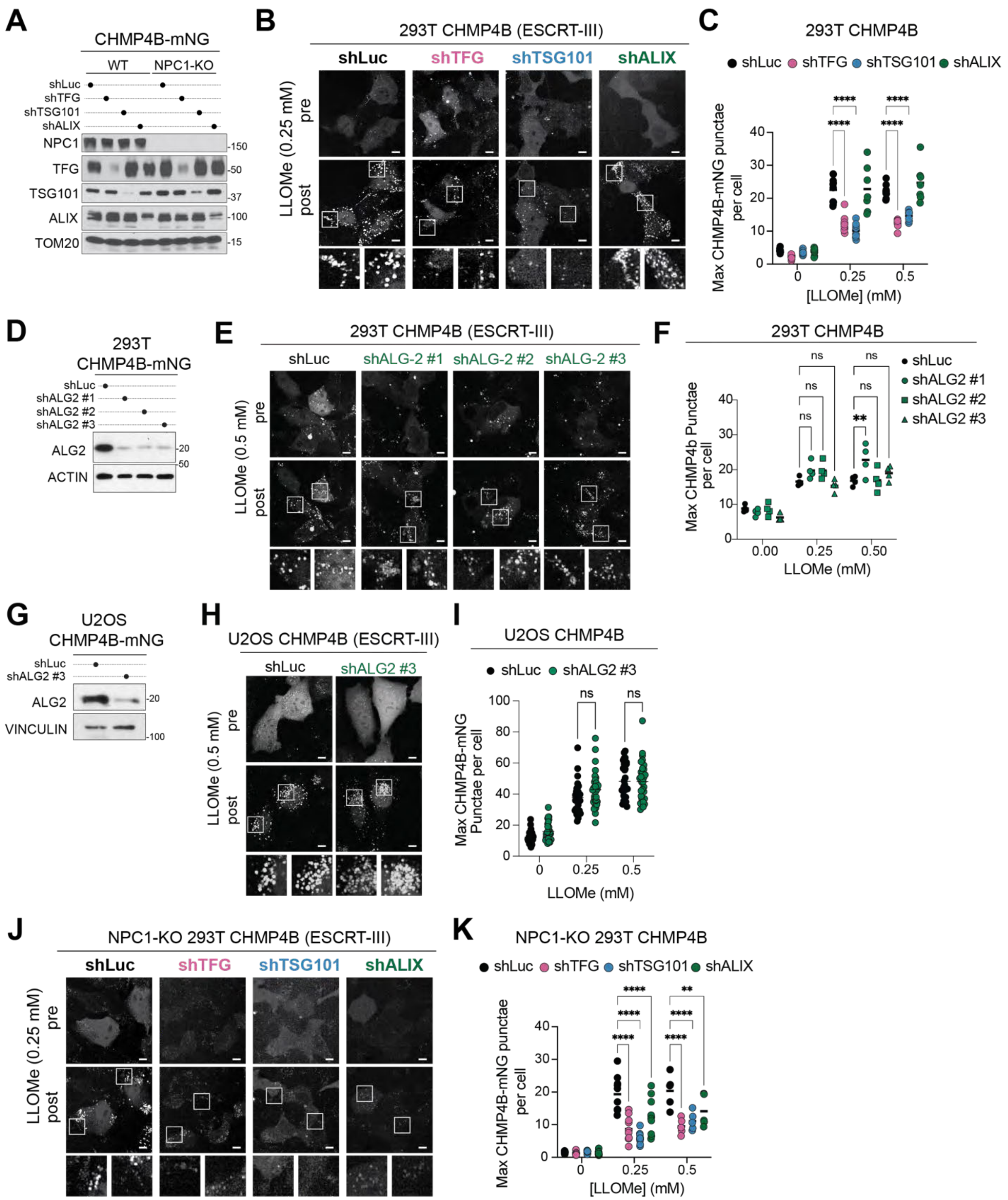

Reviewer Figure 8. TFG is critical for ESCRT recruitment and its loss phenocopies TSG101 depletion, whereas ALG2 and ALIX are largely dispensable (except for ALIX in NPC1-KO cells).

(**A**) Western blot analysis of wild-type (WT) or NPC1 knockout (KO) CHMP4B-mNG expressing 293T cells following knockdown with shRNAs targeting Luciferase (Luc) control, TFG, TSG101, or ALIX.

(**B, C**) Representative confocal images (**B**) and quantification **(C)** of maximum CHMP4B-mNG puncta formed in 293T cells treated with DMSO (vehicle control), 0.25 mM, or 0.5 mM LLOMe following treatment with shRNA targeting TFG (pink), TSG101 (blue), or ALIX (green), compared to Luc control (black). Images are shown for 5 minutes of treatment. N = 8 FOVs. Scale bars, 10 µm.

(**D**) Western blot analysis of wild-type (WT) CHMP4B-mNeonGreen (mNG) expressing 293T cells following knockdown with shRNAs targeting Luciferase (Luc) control or ALG2.
(**E, F**) Representative confocal images (**B**) and quantification (**C**) of maximum CHMP4B-mNG puncta formed in 293T cells treated with DMSO (vehicle control), 0.25 mM, or 0.5 mM LLOMe following treatment with shRNA targeting ALG2 (green) compared to Luc control (black). Images are shown for 15 minutes of treatment. N = 4 FOVs. Scale bars, 10 µm.
(**G**) Western blot analysis of wild-type (WT) CHMP4B-mNeonGreen (mNG) expressing U2OS cells following knockdown with shRNAs targeting Luciferase (Luc) control or ALG2.
(**H, I**) Representative confocal images (**E**) and quantification (**F**) of maximum CHMP4B-mNG puncta formed in U2OS cells treated with DMSO (vehicle control), 0.25 mM, or 0.5 mM LLOMe following treatment with shRNA targeting ALG2 (green) compared to Luc control (black). Images are shown for 15 minutes of treatment. N = 15 FOVs. Scale bars, 10 µm.
(**J, K**) Representative confocal images **(J)** and quantification **(K)** of maximum CHMP4B-mNG puncta formed in 293T NPC1 KO cells treated with DMSO (vehicle control), 0.25 mM, or 0.5 mM LLOMe following treatment with shRNA targeting TFG (pink), TSG101 (blue), or ALIX (green), compared to Luc control (black). Images are shown for 5 minutes of treatment. N = 9 FOVs. Scale bars, 10 µm.
Comparison of groups carried out by two-way ANOVA (**C, F, I, K**). ****p<.001, **p<0.01, ns=not significant.

2. Why were the cells challenged for 8 days with LLOMe during CRISPRi screens? LLOMe activity typically declines within hours, so the rationale for extended treatment windows (days) needs justification. In Figure 1A it is stated "2 days drug treatment." The discrepancy should be clarified.

We apologize for the confusion. In the growth-based CRISPRi screen, we did in fact add back fresh LLOMe according to a 2 days on-2 days off schedule for a total of 8 days to get sufficient (half-maximal) death for reliable changes in sgRNA abundance. We have updated the figure legend and methods accordingly to clarify these points.

3. The assembly of the LASER complex on damaged lysosomes via ATG8-LIR interactions is convincingly shown, but the mechanisms remain incompletely characterized. The mechanisms that trigger lipid conjugation and association of ATG8 proteins on damaged lysosomes have been characterized by previous studies, but two issues remain unexplained: What is the signal for dissociation of TFG from ER exit sites in response to lysosome damage, and why does TSG101 fail to be recruited to TFG at ER exit sites? The authors speculate that binding of TFG to SEC16 might prevent its binding to TSG101, but they do not provide any evidence for this.

We provide three new pieces of data that start to delineate a model for TFG translocation from ERES to lysosomes during damage. The first is that blocking CASM by ATG5-KD not only blocks TFG association with LAMP2 but also prevents dissociation from ERES marker, SEC31A (**Reviewer Fig. 9A, 9B**). The second is that co-depletion of VAPA/B, which are required for ER-lysosome contacts during damage-repair, also blocks TFG translocation to lysosomes (**Reviewer Fig. 9C-9E; Extended Data Fig. 12B-12D**).

The third piece of evidence is that calcium release from the lysosomal lumen is what triggers to CASM-dependent TFG recruitment to damaged lysosomes. Specifically, we show that TRPML1 stimulation was sufficient to recruit TFG to lysosomes to a level similar to LLOMe stimulation, and that both TRPML1 and LLOMe dependent TFG recruitment are abolished by the calcium chelator BAPTA-AM (**Reviewer Fig. 9F-9I; Fig. 2F-2G; Extended Data Fig. 15E-15F**).

Combined, these three results suggest that upon damage, calcium induces CASM and aided by the VAP-mediated proximity between ERES and lysosomes, TFG can translocate to ATG8-ylated lysosomes. It is possible that calcium may have additional functions, such as modulating TFG association to ERES proteins and/or its oligomeric state. These will be topics for future investigations.

Finally, as to why ESCRTs (TSG101) don't bind to ERES, we point out that ESCRT recruitment and assembly are highly cooperative processes that require both scaffolding and activating factors, such as ubiquitin in the context of intraluminal vesicle formation. It is, therefore,

conceivable that ERES lack such cofactors that are, instead, present and cooperate with TFG on the membranes of damaged lysosomes.

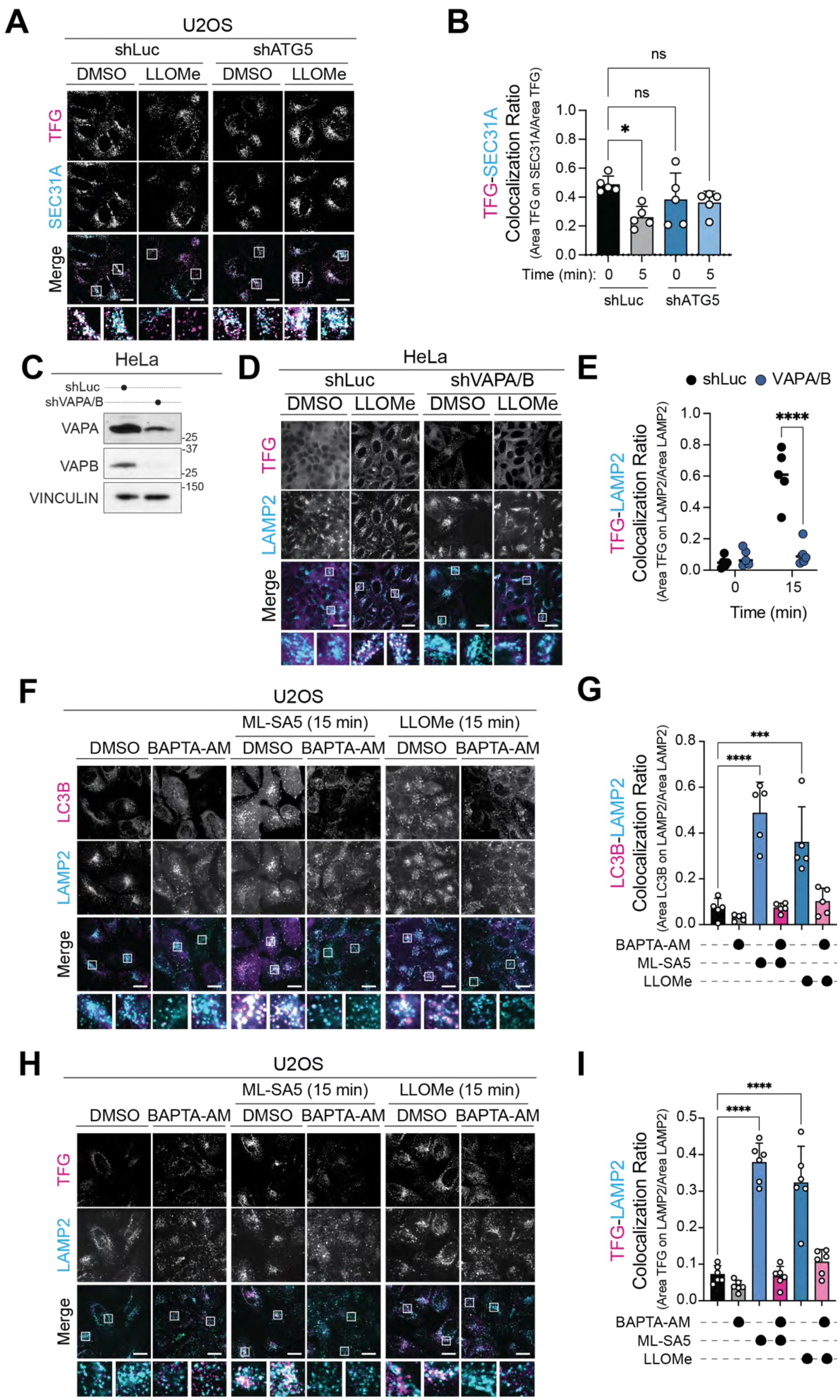

A
U2OS
shLuc
shATG5
DMSO
LLOMe
DMSO
LLOMe
TFG
SEC31A
Merge
B
TFG-SEC31A
Colocalization Ratio
(Area TFG on SEC31A/Area TFG)
ns
ns
*
1.0
0.8
0.6
0.4
0.2
0.0
Time (min): 0 5 0 5
shLuc
shATG5
C
HeLa
shLuc
shVAPA/B
VAPA
VAPB
VINCULIN
-25
-37
-25
-150
D
HeLa
shLuc
shVAPA/B
DMSO
LLOMe
DMSO
LLOMe
TFG
LAMP2
Merge
E
shLuc
VAPA/B
TFG-LAMP2
Colocalization Ratio
(Area TFG on LAMP2/Area LAMP2)
****
1.0
0.8
0.6
0.4
0.2
0.0
0
15
Time (min)
F
U2OS
ML-SA5 (15 min)
LLOMe (15 min)
DMSO
BAPTA-AM
DMSO
BAPTA-AM
DMSO
BAPTA-AM
LC3B
LAMP2
Merge
G
LC3B-LAMP2
Colocalization Ratio
(Area LC3B on LAMP2/Area LAMP2)
***
****
0.8
0.6
0.4
0.2
0.0
BAPTA-AM
ML-SA5
LLOMe
H
U2OS
ML-SA5 (15 min)
LLOMe (15 min)
DMSO
BAPTA-AM
DMSO
BAPTA-AM
DMSO
BAPTA-AM
TFG
LAMP2
Merge
I
TFG-LAMP2
Colocalization Ratio
(Area TFG on LAMP2/Area LAMP2)
****
****
0.5
0.4
0.3
0.2
0.1
0.0
BAPTA-AM
ML-SA5
LLOMe

Reviewer Figure 9. TFG translocation from ERES to lysosomes depends on ATG5, lysosomal calcium release and VAPA/B, but is ATG13-independent.

(**A, B**) Representative confocal images of TFG and SEC31A immunofluorescence staining (**A**) and quantification (**B**) of the fraction of TFG colocalized with SEC31A in U2OS cells following treatment with shRNAs targeting Luc control or ATG5, followed by treatment with DMSO (vehicle control) or 0.5 mM LLOMe for 5 minutes. N = 5 FOVs. Scale bars, 10 µm.

(**C**) Western blot analysis of HeLa cells following knockdown with shRNAs targeting Luc or VAPA and VAPB.

(**D, E**) Representative confocal images of TFG and LAMP2 immunofluorescence staining (**D**) and quantification (**E**) of the fraction of LAMP2 colocalized with TFG in HeLa cells following treatment with shRNAs targeting Luc or VAPA and VAPB followed by treatment with DMSO (vehicle control) or 0.5 mM LLOMe for 15 minutes. N = 5 FOVs. Scale bars, 10 µm.

(**F, G**) Representative confocal images (**F**) of LC3B and LAMP2 immunofluorescence staining and quantification (**G**) of colocalization ratio between LC3B and LAMP2 in U2OS cells. Cells were treated with vehicle control (DMSO) or 20 µM BAPTA-AM, followed by treatment with either 4 µM MLSA5 or 0.5 mM LLOMe for 15 minutes. N = 5 FOVs. Scale bars, 10 µm.

(**H, I**) Representative confocal images (**H**) of TFG and LAMP2 immunofluorescence staining and quantification (**I**) of colocalization ratio between TFG and LAMP2 in U2OS cells. Cells were treated with vehicle control (DMSO) or 20 µM BAPTA-AM, followed by treatment with either 4 µM MLSA5 or 0.5 mM LLOMe for 15 minutes. N = 6 FOVs. Scale bars, 10 µm.

Comparison of groups carried out by one-way (**B G, I**) or two-way ANOVA (**E**). Error bars represent mean +/- SD. ****p<0.0001, ***p<0.001, *p<0.05, ns=not significant.

4. Since ER–lysosome contacts increase upon lysosomal damage (as reported previously), is TFG recruitment linked to enrichment of ERES at damaged lysosomes? This is hinted at in Extended Figure 8, but the authors should clarify the mechanism.

We now show that abolishing ER-lysosome contacts by double knockdown of VAPA/B prevents TFG recruitment to damaged lysosomes (**Reviewer Fig. 9C-9E above**; **Extended Data Fig. 12B-12D**). This experiment shows that this general proximity between lysosomes and ER, to which ERES are contiguous, is important for TFG recruitment. However, this proximity does not seem to involve the formation of direct ERES-lysosome contacts. The evidence is that co-staining the ERES marker SEC31A with LAMP2 shows no increase in colocalization between these two compartments upon LLOMe-induced lysosomal damage in either HeLa or U2OS cells (**Reviewer Fig. 10**; **Extended Data Fig. 11A-11D**). Together with the observation that TFG-SEC31A colocalization decreases upon damage (**Extended Data Fig. 11E-11J**), these data support a model in which TFG translocates from ERES to damaged lysosomes in a VAP-dependent manner.

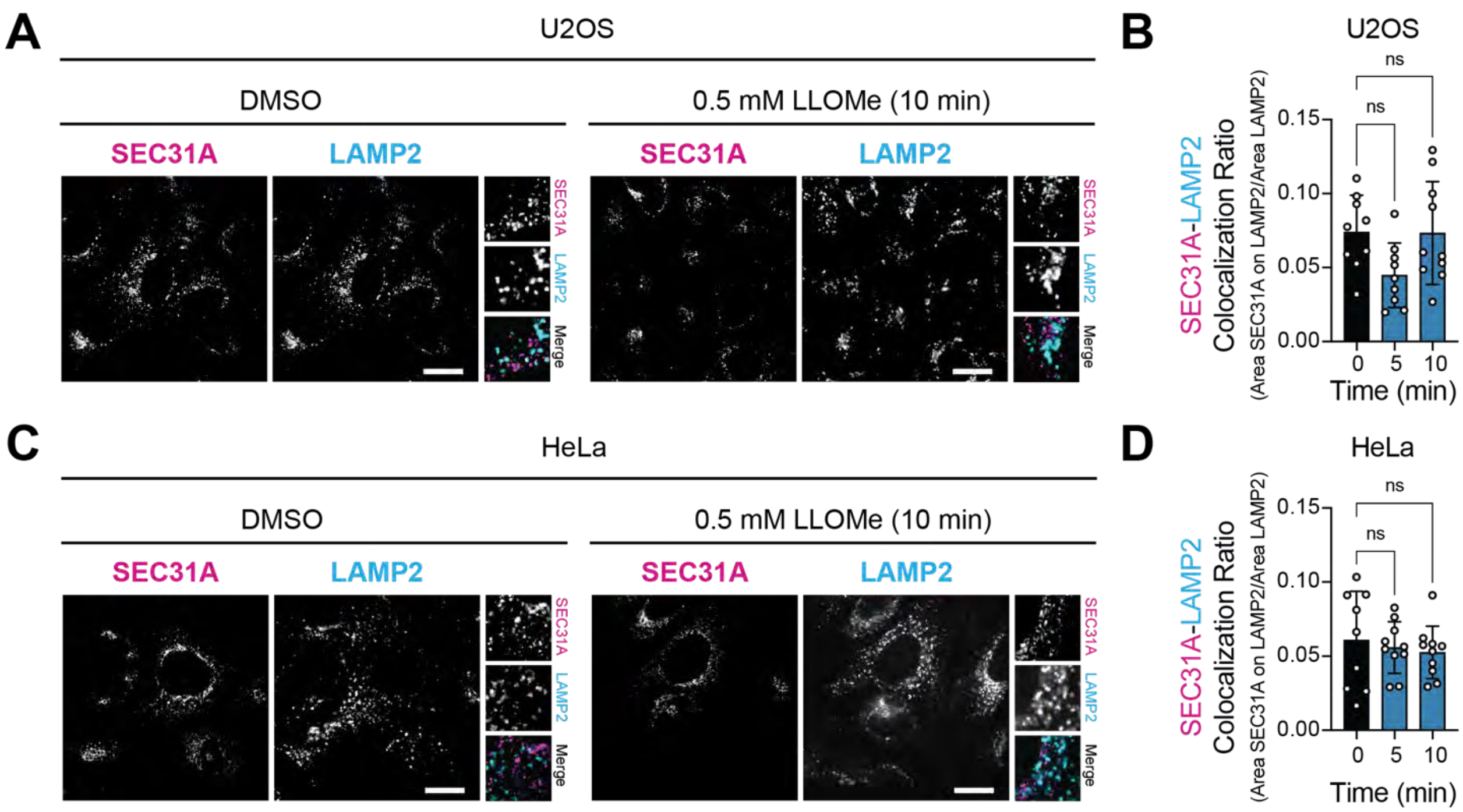

Reviewer Figure 10. Unlike TFG, SEC31A does not co-localize with damaged lysosomes.
(**A-D**) Representative confocal images of LAMP2 and SEC31A immunofluorescence staining (**A, C**) and quantification (**B, D**) of the fraction of SEC31A colocalized with LAMP2 in U2OS (**A, B**) or HeLa (**C, D**) cells following treatment with DMSO (vehicle control) or 0.5 mM LLOMe for 10 minutes. N = 10 FOVs. Scale bars, 10 µm.
Comparison of groups carried out by one-way ANOVA (**B, D**). Error bars represent mean +/- SD. ns=not significant.

5. It has been shown previously that ALG-2 acts as a Ca2+-sensitive adaptor to concentrate and polymerize TFG at ERES (Kanadome et al., FEBS J. 2017s). Do the authors find ALG2 important for TFG translocation to lysosomes? Additional studies manipulating ALG2 levels (KO or transient knockdown) should be carried out to clarify this further. Related to this, what happens to TFG recruitment/localization in the presence or absence of Ca2+?

We found that ALG2 knockdown had no effect on ESCRT-III assembly on damaged lysosomes (**Reviewer Fig. 8D-8I above; Extended Data Fig. 14D-14I**). Moreover, TFG recruitment to LLOMe-damaged lysosomes was not affected by ALG2 knockdown (**Reviewer Fig. 11A-11C; Extended Data Fig. 14A-14C**). Conversely, ALG2 was still recruited to damaged lysosomes in TFG-depleted cells (**Reviewer Fig. 11D**). Together, these data further indicate that the TFG-TSG101 pathway is dominant for ESCRT recruitment and independent of ALG2.

To test the role of Ca^{2+} in TFG recruitment to damaged lysosomes, we stimulated the cells with the TRPML1 agonist, ML-SA5, which was previously shown to trigger lysosomal membrane ATG8-ylation. We saw that, in fact, TRPML1 activation was sufficient to induce CASM and lysosomal TFG recruitment independent from, and to a level comparable to, LLOMe treatment, and that this response was abolished by calcium chelation by BAPTA-AM (**Reviewer Fig. 9F-9I above; Fig. 2F-2G; Extended Data Fig. 15E-15F**). Consistently, TRPML1 activation was also sufficient to induce ESCRT-III recruitment to (undamaged) lysosomes, a response also blocked by BAPTA-AM in accordance with previous reports[1] (**Reviewer Fig. 11E-11H; Extended Data Fig. 13A-13D**). Together, these data point to calcium induced LASER assembly as the key driver for ESCRT recruitment.

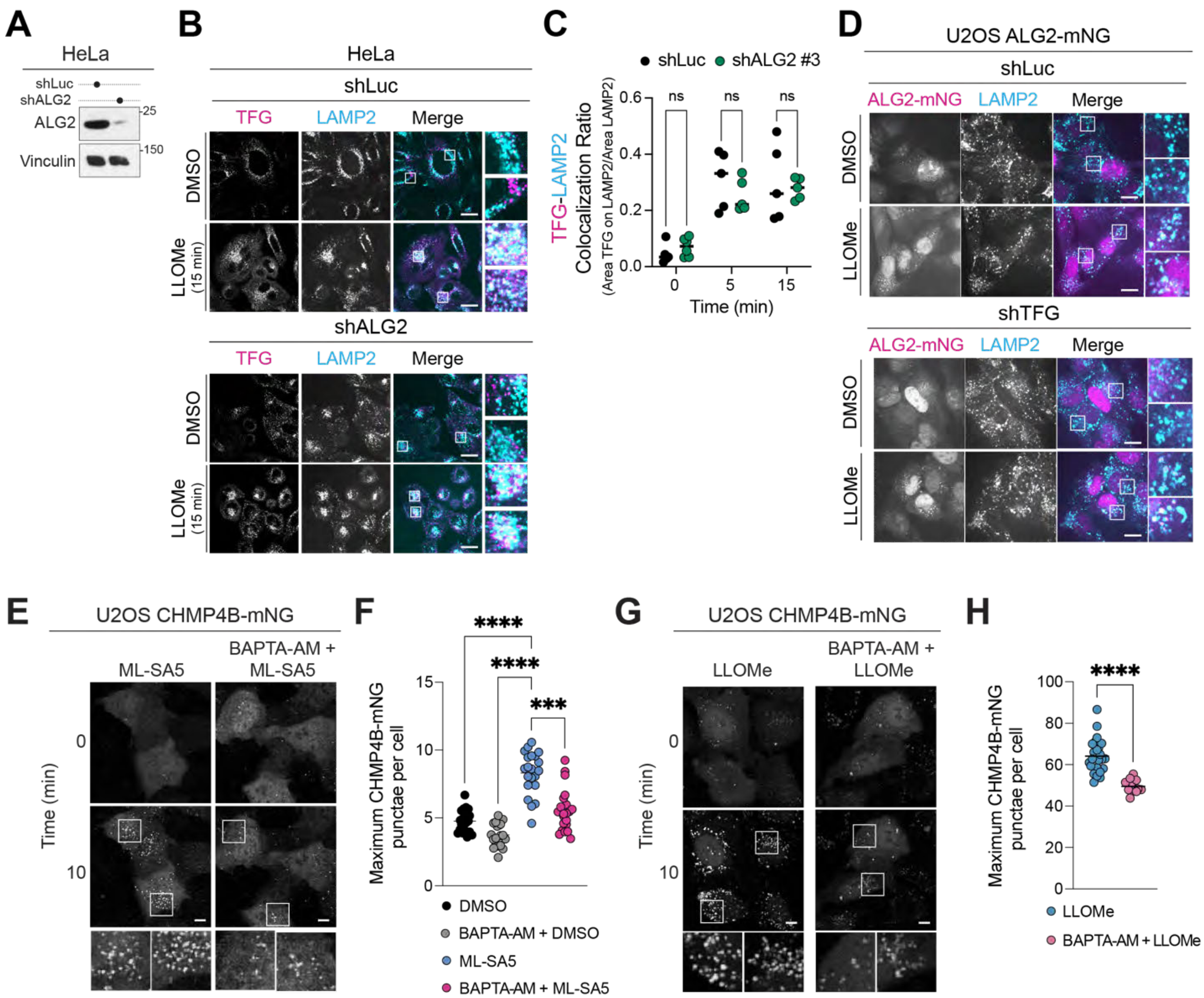

Reviewer Figure 11. TFG and ALG2 function independently and, like TFG, CHMP4B recruitment to damaged lysosomes is TRPML1-induced and calcium-dependent.
(**A**) Western blot analysis of HeLa cells following knockdown with shRNAs targeting Luciferase (Luc) control or ALG2.
(**B, C**) Representative confocal images (**B**) of TFG and LAMP2 immunofluorescence staining and quantification (**C**) of colocalization ratio between TFG and LAMP2 in HeLa cells. Cells were transduced with shRNAs targeting ALG2 or Luc, followed by treatment with vehicle control (DMSO) or 0.5 mM LLOMe for 5 or 15 minutes. Images are shown for 15 minutes of treatment. N = 5 FOVs. Scale bars, 10 μm.
(**D**) Representative confocal images of ALG2-mNeonGreen (mNG) expressing U2OS cells with LAMP2 immunofluorescence staining following treatment with 0.5 mM LLOMe and knockdown with shRNAs targeting Luc or TFG. Scale bars, 10 μm.
(**E, F**) Representative confocal images (**E**) and quantification (**F**) of maximum CHMP4B-mNeonGreen puncta in U2OS cells upon treatment with DMSO or 4 μM ML-SA5, with or without pre-treatment for 1 hour with 20 μM BAPTA-AM. Images are shown for 10 minutes of treatment. N = 21 FOVs. Scale bars, 10 μm.
(**G, H**) Representative confocal images (**G**) and quantification (**H**) of maximum CHMP4B-mNeonGreen puncta in U2OS cells upon treatment with LLOMe, with or without pre-treatment for 1 hour with 20 μM BAPTA-AM. Images are shown for 10 minutes of treatment. N = 24 FOVs. Scale bars, 10 μm.
Comparison of groups carried out by two-way (**C**), one-way (**F**) ANOVA or unpaired t-test (**H**). ****p<0.0001, ***p<0.001, ns=not significant.

6. How does TFG loss affect cholesterol levels? Could the observed phenotype upon lysosome damage be lipid-driven?

We checked this possibility by performing Filipin staining upon TFG knockdown and found no difference compared to Luc control cells, whereas treatment with the NPC1 inhibitor, U18666A, induced clear lysosomal cholesterol accumulation, regardless of TFG status (**Reviewer Fig. 12**).

We also point out that TFG is essential for ESCRT recruitment in both NPC1-KO and WT cells, indicating that TFG is likely required downstream of many kinds of membrane damage (cholesterol imbalance, chemical agents, etc.) (**Fig. 1G-1H; Extended Data Fig. 9**).

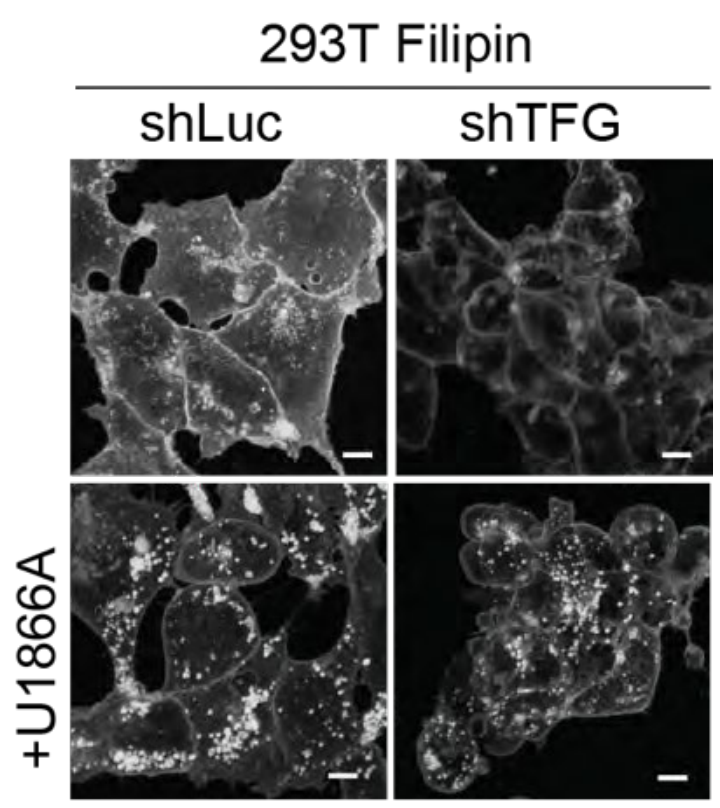

Reviewer Figure 12. TFG depletion does not induce lysosomal cholesterol sequestration.
Representative confocal images of 293T cells stained with filipin, with or without treatment with 5 µg/mL U1866A for 16 hours. Cells were treated with shRNAs targeting Luciferase (Luc) control or TFG. Scale bars, 10 µm.

7. Can the authors insert the LIR2 domain from TFG to other ERES proteins such as SEC13, SEC31A or SEC16 and investigate whether those proteins exhibit the same behavior as TFG upon lysosome damage?

That is an interesting idea; however, given that all these proteins have important roles in other multimeric protein complexes (ex. SEC13 in COPII vesicles and GATOR2 complex), we are concerned that any result would be difficult to interpret.

8. Among the sensitizing hits in Extended Data Figure 2 there are several ESCRT proteins annotated as direct hits in the screen, and the figure also includes "coessential genes" from DepMap. The latter leads to some confusion since the network annotated as "ESCRT-I/II complex" contains the ESCRT-0 subunit HGS and the ESCRT-0 binding protein STAMBP. As the authors mention in the Introduction, ESCRT-0 is not involved in recruitment of the other ESCRTs to damaged lysosomes, so this raises questions about the relevance of including the DepMap data in the figure.

Thank you for pointing this out. We retain the DepMap coessentiality analysis because it was critical for identifying TFG as coessential with early ESCRT pathway components. Importantly, this coessentiality analysis is agnostic the specific membrane-remodeling context in which ESCRT proteins function. Therefore, ESCRT-0 components cluster with ESCRT-I/II based on shared genetic dependencies across many cellular processes, even though ESCRT-0 has not been shown to be required for ESCRT recruitment to damaged lysosomes. We view this as a strength of the unbiased network-based approach, which groups proteins that function together in the broader ESCRT pathway rather than only in lysosomal repair.

To avoid confusion, we have relabeled this subnetwork as Early ESCRT (0/I/II) and clarified in the figure legend which nodes represent direct CRISPRi screen hit versus DepMap coessential genes.

9. Also related to Extended Data Figure 2, the ESCRT-II components of the "ESCRT-I/II complex" in this figure are all from DepMap and therefore fall into the same category as HGS and STAMBP. In the absence of experimental data showing that ESCRT-II is required for lysosome repair (it may be recruited to damaged lysosomes as a passenger with ESCRT-I) the authors should be cautious with implicating ESCRT-II in the repair process.

We understand the reviewer's concern. The current model of ESCRT-mediated repair is based on our understanding of ILV formation, where ESCRT-II is an essential bridge between ESCRT-I and ESCRT-III. While it is true that this concept has not been as thoroughly established in the context of lysosomal repair, we note that TFG depletion led to failed ESCRT-II recruitment, as it did for ESCRT-I and ESCRT-III.

10. Studies with patient-derived fibroblasts are inherently associated with difficulties in obtaining appropriate controls, and the results with these would be strengthened by rescue experiments with wild-type and mutant TFG.

We completely agree with the reviewer and now provide these rescue experiments. Indeed, re-expression of wild-type TFG, but not R106C-mutant or LIR2-mutant TFG, in the R106C homozygous fibroblasts restored lysosomal recruitment of CHMP1A following LLOMe treatment (**Reviewer Fig. 13A-13B; Fig. 5E-5F**). Wild-type TFG, but not R106C-mutant or LIR2-mutant TFG, also rescued the lysosomal repair defect in the R106C fibroblasts, as measured by the dextran-FITC dequenching assay (**Reviewer Fig. 13C-13D; Fig. 5G-5H**). Together, these results strengthen the conclusion that the observed lysosomal phenotypes in the HSP patient fibroblasts are due to the loss of functional TFG.

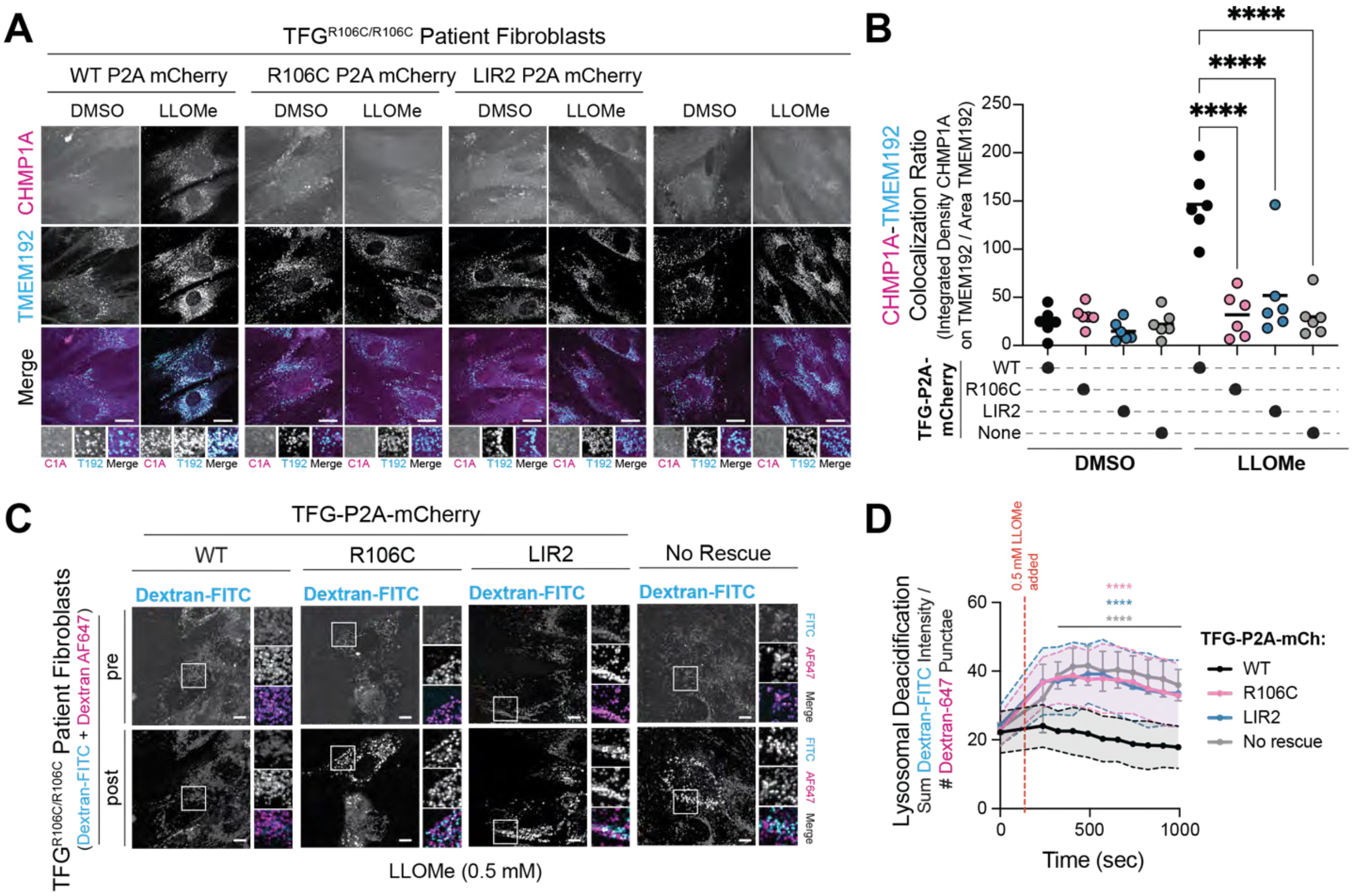

Reviewer Figure 13. Wild-type TFG rescues CHMP1A recruitment and lysosomal repair in the HSP patient (TFGR^{106C}) fibroblasts, whereas the R106C and LIR2 mutants of TFG fail to do so.
(**A, B**) Representative immunofluorescence images (**A**) and quantification (**B**) of colocalization between CHMP1A and TMEM192 in fibroblasts from HSP patients with homozygous R106C mutation (TFG$^{R106C/R106C}$) reconstituted with either wild-type (WT), R106C mutant, or LIR2 mutant TFG, treated with 0.5 mM LLOMe. Images are shown following 7 minutes of treatment. Scale bars, 10 µm. N = 6 FOVs.
(**C, D**) Representative confocal images (**C**) and quantification (**D**) of total fluorescence intensity ratio between internalized dextran (FITC vs TMR) punctae following treatment with 0.5 mM LLOMe. Fibroblasts from HSP patients with homozygous R106C mutation (TFG$^{R106C/R106C}$) were reconstituted with either wild-type (WT), R106C mutant, or LIR2 mutant TFG. Scale bars, 10 µm. N = 11 FOVs.
Comparison of groups carried out by one-way (**B**) or two-way ANOVA (**D**). Error bars represent mean +/- SD. ****p<0.0001.

11. Sometimes mutations of LIR motifs affect interactions with ATG8 proteins because of indirect effects on protein folding or stability. Can this be excluded in the case of TFG? A biochemical experiment showing direct interactions between TFG and an ATG8 protein would be informative.

We thank the reviewer for raising this important point. We assessed whether TFG and ATG8 could directly interact in an equilibrium binding assay. We purified a truncated mCherry-TFG$^{1\text{-}210}$ (the full length, including the C-terminal intrinsically disordered region, could not be purified due to aggregation and precipitation), both wild-type and LIR2-mutant, and combined it with agarose beads coated with Flag-GFP-LC3B. Recombinant wild-type TFG exhibited detectable binding to bead-bound LC3B (**Reviewer Fig. 14A-14B**). Despite identical expression and stability to wild-type (ruling out destabilizing effects of the LIR2 mutation) (**Reviewer Fig. 14C**), the LIR2 mutant of TFG$^{1\text{-}210}$ failed to display any detectable binding to LC3B (**Reviewer Fig. 14A-14B**).

In line with the lower affinity of the TFG-LIR2 peptide for LC3B versus the affinity of the p62-LIR peptide (~60 µM for TFG-LIR2 versus ~2 µM for p62) (**Extended Data Fig 18A-18D**), the binding of wild-type mCherry-TFG$^{1\text{-}210}$ to LC3B containing beads was much weaker than the same amount of mCherry-p62 (**Reviewer Fig. 14C-14D**). Despite this weak TFG-LC3B affinity

under these *in vitro* conditions (perhaps due to a lack of the C-terminal region and/or a need for more complex reconstitution), this interaction is clearly critical for TFG recruitment and subsequent ESCRT assembly and lysosomal repair, as further shown by our new data in the HSP patient fibroblasts (**Reviewer Fig. 13A-13D above**; **Fig. 5E-5H**).

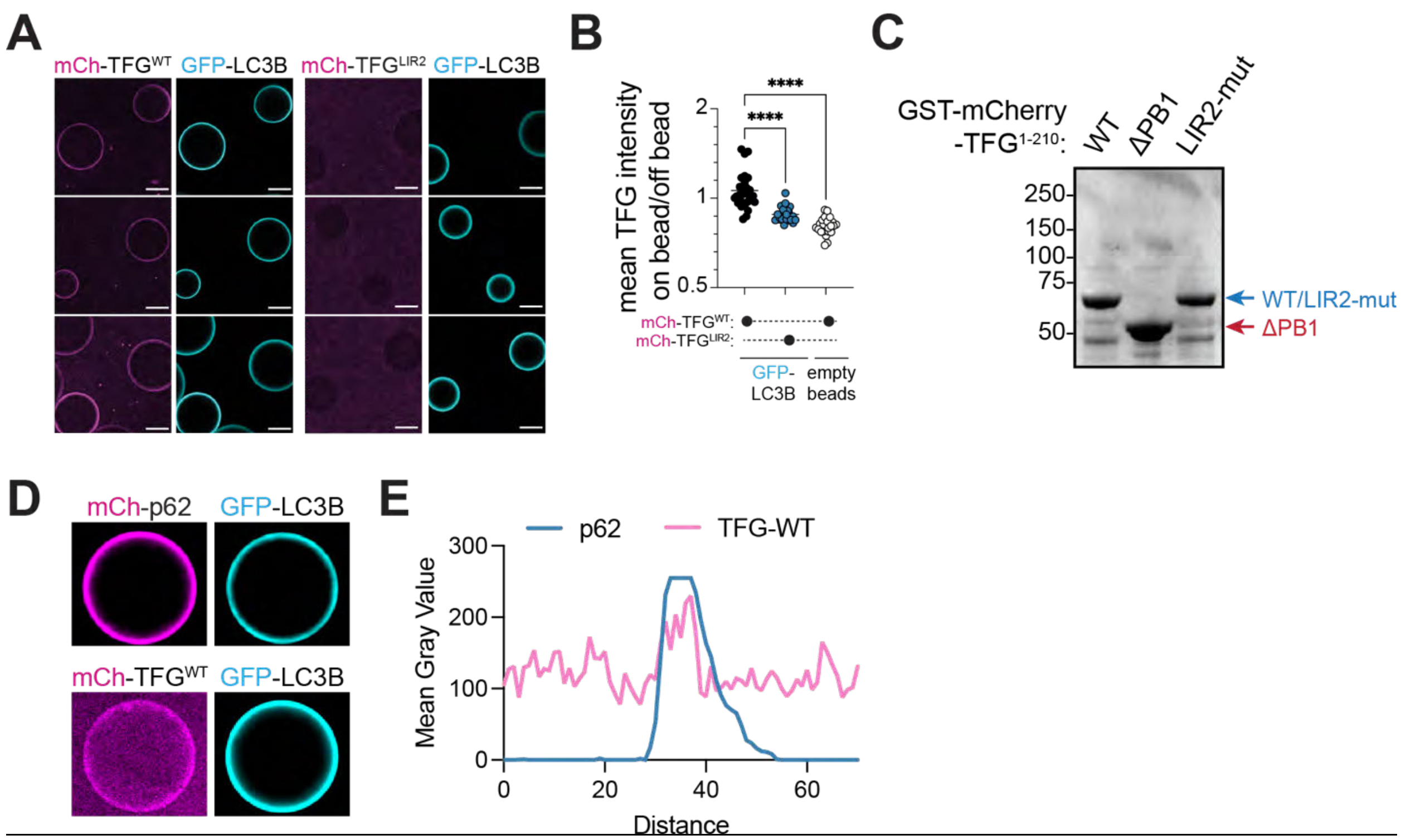

Reviewer Figure 14. Recombinant TFG$^{1\text{-}210}$ binds to LC3B in vitro in a LIR2-dependent manner, but with lower affinity than p62.

(**A, B**) Representative confocal images (**A**) and quantification (**B**) of binding between recombinant mCherry-TFG$^{1\text{-}210}$, wild-type (WT; *left*) or LIR2-mutant (*right*), and GFP-trap beads coated with Flag-GFP-LC3B. Quantification of 16-30 individual beads.

(**C**) Coomassie staining of purified recombinant 6xHis-mCherry-TFG$^{1\text{-}210}$ WT versus mutant (ΔPB1 and LIR2-mut) proteins.

(**D, E**) Representative confocal images (**D**) and line graph quantification (**E**) of bead bound mCherry-p62 (*top*) versus mCherry-TFG$^{1\text{-}210}$ WT (*bottom*) recombinant proteins. GFP-trap beads were coated with Flag-GFP-LC3B.

12. In contrast to Extended Figure 4D-E, in Fig. 1E Galectin-3 staining at 90 min post-LLOMe appears unchanged in shLuc. This should be explained.

Fig. 1E-1F was performed in wild-type 293T cells, whereas **Extended Fig. 4D-4E** was performed in NPC1-KO 293T cells (now clearly indicated in the revised submission). Both assays used a 15-min pulse of low-dose LLOMe (0.125 mM) to elicit mild lysosomal membrane damage and assess subsequent repair. Under these conditions, persistent or propagated damage leads to increased galectin recruitment, whereas efficient repair yields minimal signal. Consistent with this design, wild-type 293T cells exhibit negligible galectin recruitment, indicating effective repair, while TFG depletion increases galectin accumulation (**Fig. 1E-1F**). In NPC1-KO 293T cells, which are more damage-prone, TFG loss further augments galectin recruitment, although baseline damage is elevated even in shLuc controls (**Extended Data Fig. 4D-4E**).

13. Fig. 1E–G -F/ Extended Fig. 4D: Why do LLOMe concentrations vary between experiments?

As noted above, the galectin recruitment assays (**Fig. 1E-1F; Extended Data Fig. 4D-4E**) use a brief (15 min) pulse of low-dose LLOMe (0.125 mM) to induce minimal lysosomal damage and allow evaluation of repair during the chase period. In contrast, ESCRT recruitment (CHMP4B and UBAP1) is assessed using sustained LLOMe exposure without a chase, enabling direct measurement of repair machinery recruitment rather than repair capacity. Thus, the galectin assay probes the extent of lysosomal repair, whereas the ESCRT assay probes the ability to initiate repair.

14. Fig. 2C, F: Fluorescence and EM images suggest that TFG might localize inside lysosomes. Could the authors speculate on possible mechanisms or interpretations?

Our imaging and biochemical data clearly shows that the bulk of TFG localizes to the cytoplasmic face of the lysosomal membrane. However, the reviewer raises the intriguing possibility that a fraction of TFG, bound to polymerized ESCRT, may be internalized into the lysosomal as part of the ILV budding process. The resolution limits of confocal imaging and the sparse labeling nature of immunogold EM makes it difficult to quantify what this fraction could be.

Of note, we had concerns about the **original Fig. 2C-2E and Extended Data Fig. 7C-7G**. In those engineered RPE-1 cells in which endogenous SEC16 is Halo-tagged, we observed an abnormal degree of Halo-SEC16 clustering outside of ERES. Therefore, we have replaced those original panels with equivalent data in naïve, non-engineered, RPE-1 cells, which confirm colocalization between endogenous TFG and LAMP2 (**Extended Data Fig. 10C-10D**), and the decreased colocalization between TFG and SEC31A (**Extended Data Fig. 11H-11J**) upon LLOMe treatment.

15. Fig. 3H: These data should be shown in the Extended figures.

We thank the reviewer for their suggestion and have now moved these data to **Extended Data Fig. 18F-18G**.

Referee #3 (Remarks to the Author):

I co-reviewed this manuscript with one of the reviewers who provided the listed reports.

We thank the reviewer for their input.

Referee #4 (Remarks to the Author):

Lysosome repair has emerged as an important aspect of organelle quality control and has been implicated in multiple human diseases, including neurodegeneration. Over the past decades, several factors such as lipid transfer proteins, ESCRTs, and CASM have been identified as contributing to this process. However, the early steps that connect the sensing of lysosomal damage to the recruitment of repair machinery, such as ESCRTs, remain poorly understood.
The senior author's group has been at the forefront of lysosome biology, publishing a series of impactful studies in recent years. In the present work, they employ an elegant genetic screening approach that identifies a novel factor, TFG, as a key player in lysosome repair. Through comprehensive analyses including live-cell imaging and biochemical assays, the authors delineate a new bridging mechanism that links damage sensing to ESCRT recruitment. I find the discovery of this mechanism convincing and believe it represents a major advance in the

lysosome biology field. Below, I provide several suggestions that may help further refine the mechanistic insights and improve data presentation.

We thank the reviewer for acknowledging the significance and rigor of our study.

Major Comments
1. Specificity of the TFG–ATG8 mechanism
The study shows that TFG recognizes ATG8 on damaged lysosomes, thereby facilitating ESCRT recruitment. However, ATG8 proteins are activated in multiple contexts, including autophagy. If TFG recruitment depends solely on its association with ATG8, one could envision that TFG may also act on autophagosomes or other ATG8-decorated membranes. Indeed, TFG has previously been reported to associate with LC3 to regulate autophagosome biogenesis (PMID: 33932238), and ESCRT subunits have been implicated in autophagosome closure (PMID: 30030437). It would therefore be important to clarify whether this mechanism is unique to lysosome repair or extends to other ATG8-dependent pathways.

In my view, lysosome repair is distinct from autophagy because it requires the recruitment of broader ESCRT components, whereas autophagy primarily involves ESCRT-III. Additional factors may favor TFG recruitment specifically to damaged lysosomes. These may include:

- *ER–lysosome contact sites, as supported by EM data in the current study and prior work (Tan et al., PMID: 36071159).*
- *Calcium-regulated assembly, as TFG oligomerization is known to be modulated by ALG-2 (PMID: 27813252), a factor also implicated in lysosome repair.*

To strengthen this point, I suggest testing:

a. Whether TFG recruitment to damaged lysosomes requires ER–lysosome contacts.

Thank you for the suggestion. Indeed, co-depletion of VAPA/B, which are required for ER-lysosome contacts during damage-repair[4], also blocks TFG translocation to lysosomes (**Reviewer Fig. 15A-15C; Extended Data Fig. 12B-12D**). This result suggests that upon damage, ERES are brought into proximity to lysosomes via canonical, VAP-mediated, ER-lysosome contacts. In turn, this proximity may enable TFG to be engaged by ATG8 proteins, resulting in its accumulation on the lysosomal membrane.

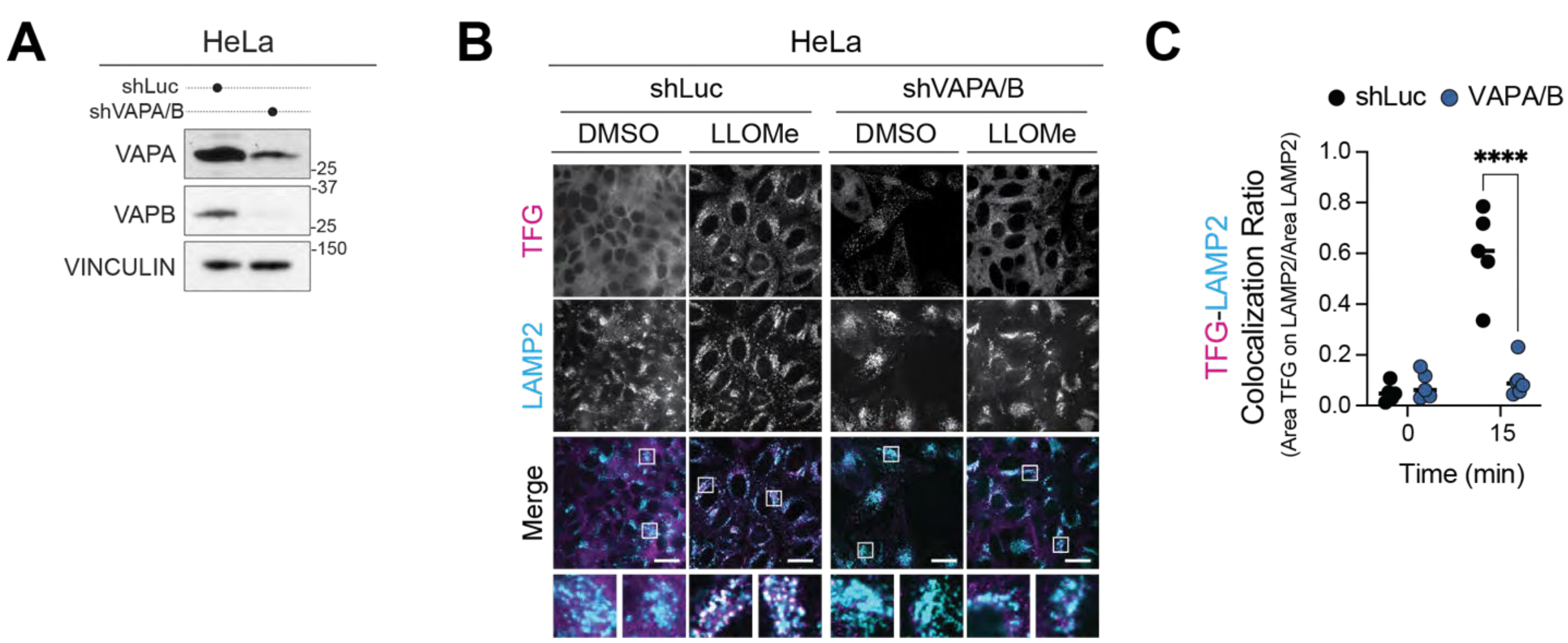

Reviewer Figure 15. VAPA/B are required for LLOMe-induced lysosomal translocation of TFG.

(**A**) Western blot analysis of HeLa cells following knockdown with shRNAs targeting Luc or VAPA and VAPB.
(**B, C**) Representative confocal images of TFG and LAMP2 immunofluorescence staining (**B**) and quantification (**C**) of the fraction of LAMP2 colocalized with TFG in HeLa cells following treatment with shRNAs targeting Luc or VAPA and VAPB followed by treatment with DMSO (vehicle control) or 0.5 mM LLOMe for 15 minutes. N = 5 FOVs. Scale bars, 10 µm.
Comparison of groups carried out by one-way ANOVA (**C**). ****p<0.0001.

b. Whether ER exit sites (ERES) physically contact damaged lysosomes.

Co-staining the ERES marker SEC31A with LAMP2 shows no increase in colocalization between these two compartments upon LLOMe-induced lysosomal damage in HeLa or U2OS cells (**Reviewer Fig. 10; Extended Data Fig. 11A-11D**). Together with the observation that TFG-SEC31A colocalization decreases upon damage (**Extended Data Fig. 11E-11J**), these data support a model in which TFG translocates from ERES to damaged lysosomes rather than direct ERES-lysosome contacts.

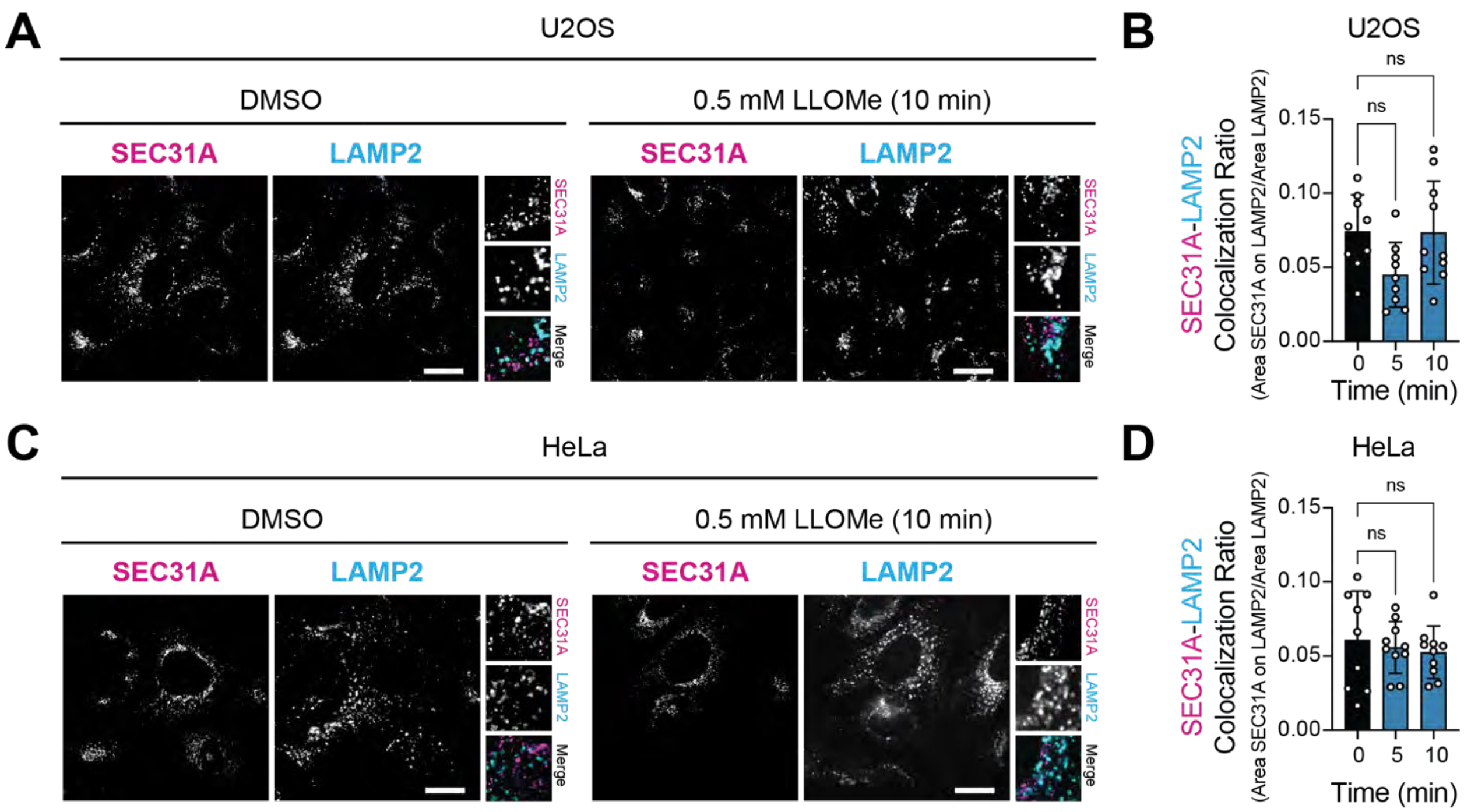

Reviewer Figure 10. Unlike TFG, SEC31A does not co-localize with damaged lysosomes.
(**A-D**) Representative confocal images of LAMP2 and SEC31A immunofluorescence staining (**A, C**) and quantification (**B, D**) of the fraction of SEC31A colocalized with LAMP2 in U2OS (**A, B**) or HeLa (**C, D**) cells following treatment with DMSO (vehicle control) or 0.5 mM LLOMe for 10 minutes. N = 10 FOVs. Scale bars, 10 µm.
Comparison of groups carried out by one-way ANOVA (**B, D**). Error bars represent mean +/- SD. ns=not significant.

c. Whether calcium signaling and ALG-2 are involved for TFG recruitment. Notably, the enlargement of TFG puncta upon lysosomal damage (Figure S7C) supports a role for calcium-enhanced assembly.

We thank the reviewer for their observation. To test whether calcium signaling is involved in TFG recruitment, we stimulated cells with the TRMPL1 agonist, ML-SA5, which was previously also shown to trigger lysosomal membrane ATG8ylation[5]. In support of a role for lysosomal calcium release in TFG recruitment, we observed that TRPML1 activation was sufficient to induce both CASM and lysosomal TFG recruitment to a level comparable to LLOMe treatment, and that this

response was abolished by calcium chelation by BAPTA-AM (**Reviewer Fig. 6A-6D; Fig. 2F-2G; Extended Data Fig. 15E-15F**). Consistently, TRPML1 activation was also sufficient to induce ESCRT-III recruitment to (undamaged) lysosomes, a response also blocked by BAPTA-AM in accordance with previous reports[1] (**Reviewer Fig. 6E-6H; Extended Data Fig. 13A-13D**). Together, these data point to calcium induced LASER assembly as the key driver for ESCRT recruitment.

Unlike calcium, we did not find evidence for ALG2 involvement in TFG recruitment. Upon ALG2 knockdown, we observed no change in TFG recruitment to LLOMe-damaged lysosomes (**Reviewer Fig. 16A-16C below; Extended Data Fig. 14A-14C**). Additionally, knocking down ALG2 in both 293T and U2OS cells showed no defect in ESCRT-III recruitment upon LLOMe treatment (**Reviewer Fig. 16C-16H below; Extended Data Fig. 14D-14I**).

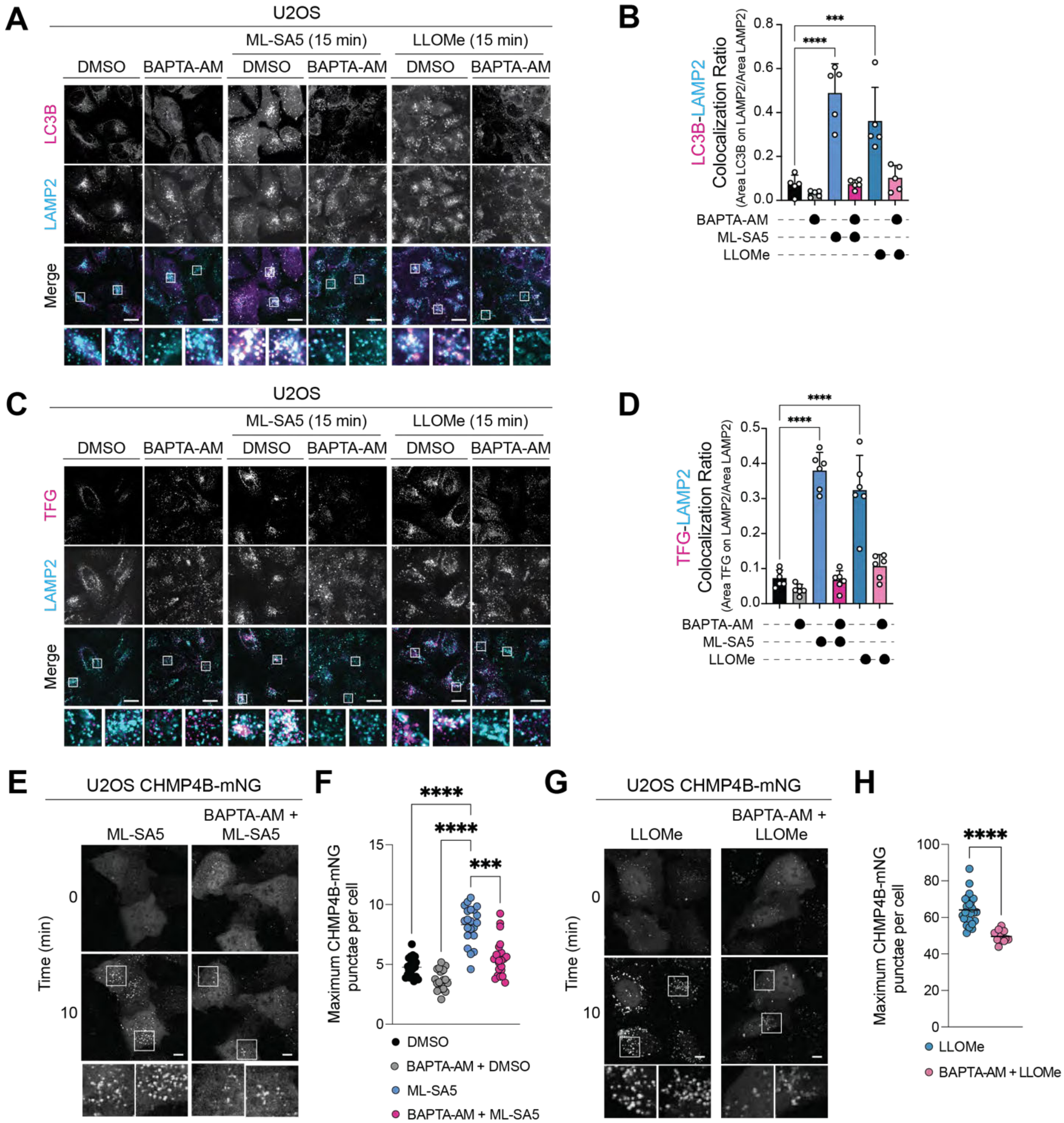

Reviewer Figure 6. Lysosomal calcium is necessary and sufficient for TFG recruitment and ESCRT assembly.
(**A, B**) Representative confocal images (**A**) of LC3B and LAMP2 immunofluorescence staining and quantification (**B**) of colocalization ratio between LC3B and LAMP2 in U2OS cells. Cells were treated with vehicle control (DMSO) or 20 µM BAPTA-AM, followed by treatment with either 4 µM MLSA5 or 0.5 mM LLOMe for 15 minutes. N = 5 FOVs. Scale bars, 10 µm.
(**C, D**) Representative confocal images (**C**) of TFG and LAMP2 immunofluorescence staining and quantification (**D**) of colocalization ratio between TFG and LAMP2 in U2OS cells. Cells were treated with vehicle control (DMSO) or 20 µM BAPTA-AM, followed by treatment with either 4 µM MLSA5 or 0.5 mM LLOMe for 15 minutes. N = 6 FOVs. Scale bars, 10 µm.
(**E, F**) Representative confocal images (**E**) and quantification (**F**) of maximum CHMP4B-mNeonGreen puncta in U2OS cells upon treatment with DMSO or 4 µM ML-SA5, with or without pre-treatment for 1 hour with 20 µM BAPTA-AM. Images are shown for 10 minutes of treatment. N = 21 FOVs. Scale bars, 10 µm.

(**G, H**) Representative confocal images (**G**) and quantification (**H**) of maximum CHMP4B-mNeonGreen puncta in U2OS cells upon treatment with LLOMe, with or without pre-treatment for 1 hour with 20 μM BAPTA-AM. Images are shown for 10 minutes of treatment. N = 24 FOVs. Scale bars, 10 μm.

Comparison of groups carried out by one-way ANOVA (**B, D, F**) or unpaired t-test (**H**). Error bars represent mean +/- SD. ****p<0.0001, ***p<0.001.

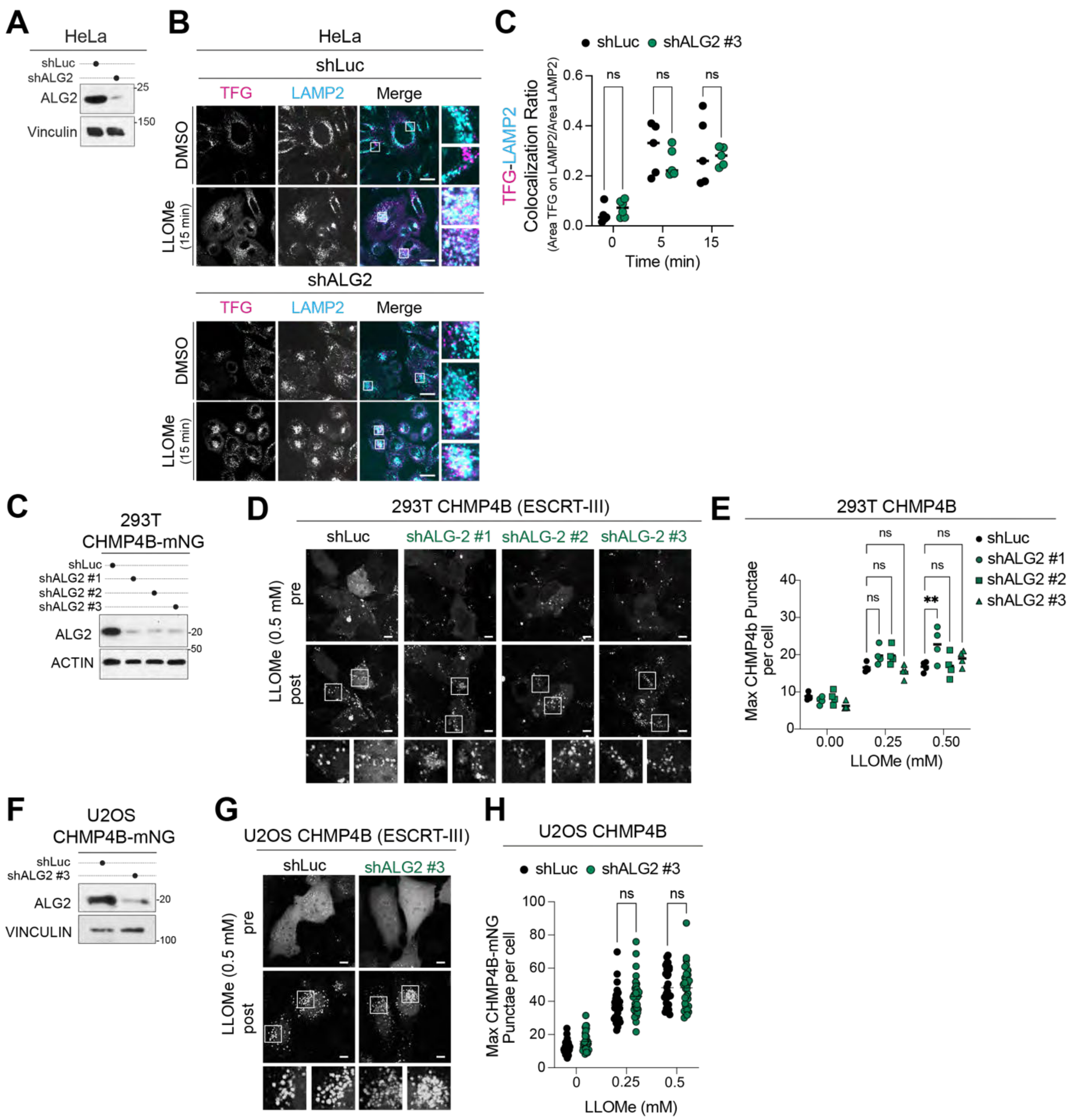

Reviewer Figure 16. ALG2 is dispensable for TFG and CHMP4B recruitment to damaged lysosomes.

(**A**) Western blot analysis of HeLa cells following knockdown with shRNAs targeting Luciferase (Luc) control or ALG2.

(**B, C**) Representative confocal images (**B**) of TFG and LAMP2 immunofluorescence staining and quantification (**C**) of colocalization ratio between TFG and LAMP2 in HeLa cells. Cells were transduced with shRNAs targeting ALG2 or Luc, followed by treatment with vehicle control (DMSO) or 0.5 mM LLOMe for 5 or 15 minutes. Images are shown for 15 minutes of treatment. N = 5 FOVs. Scale bars, 10 μm.

(**C**) Western blot analysis of wild-type (WT) CHMP4B-mNeonGreen (mNG) expressing 293T cells following knockdown with shRNAs targeting Luc control or ALG2.
(**D, E**) Representative confocal images (**D**) and quantification (**E**) of maximum CHMP4B-mNG puncta formed in 293T cells treated with DMSO (vehicle control), 0.25 mM, or 0.5 mM LLOMe following treatment with shRNA targeting ALG2 (green) compared to Luc control (black). Images are shown for 15 minutes of treatment. N = 4 FOVs. Scale bars, 10 µm.
(**F**) Western blot analysis of wild-type (WT) CHMP4B-mNeonGreen (mNG) expressing U2OS cells following knockdown with shRNAs targeting Luc control or ALG2.
(**G, H**) Representative confocal images (**G**) and quantification (**H**) of maximum CHMP4B-mNG puncta formed in U2OS cells treated with DMSO (vehicle control), 0.25 mM, or 0.5 mM LLOMe following treatment with shRNA targeting ALG2 (green) compared to Luc control (black). Images are shown for 15 minutes of treatment. N = 15 FOVs. Scale bars, 10 µm.
Comparison of groups carried out by two-way ANOVA (**C, E, H**). **p<0.01, ns=not significant.

d. As an additional experiment, testing whether TFG–ATG8 interactions contribute to autophagosome closure by recruiting ESCRTs could broaden the appeal of this work to autophagy researchers.

We appreciate the suggestion. However interesting, properly answering this question would require an entirely separate study due to the structural and compositional differences between a damaged lysosome and a growing autophagosome.

2. Patient fibroblast experiments (Figure 5)
The R106C TFG mutant is defective not only in lysosome repair but also in ER exit site function and autophagy (PMID: 33932238). These broader defects could indirectly contribute to the lysosomal phenotypes observed. I recommend either testing an additional TFG mutant or explicitly discussing these alternative possibilities in the manuscript.

As suggested, we tested the ability of other disease-associated mutants of TFG to support lysosomal repair. Specifically, we examined the R22W and P285L mutants in our knockdown-rescue system. The R22W mutation, located in the PB1 domain and associated with early-onset HSP, has been reported to impair TFG ring assembly and impact secretory trafficking[6–8]. However, this mutant did not show an appreciable defect in lysosomal membrane repair (**Reviewer Fig. 17A-17B**). In contrast, we found that P285L mutant exhibited a similar ESCRT recruitment defect as R106C (**Reviewer Fig. 17A-17B**). Hence, the lysosomal repair and ERES secretory defect are, at least in part, separable. Further studies are required to dissect the relative roles of these two defects on neuronal viability.

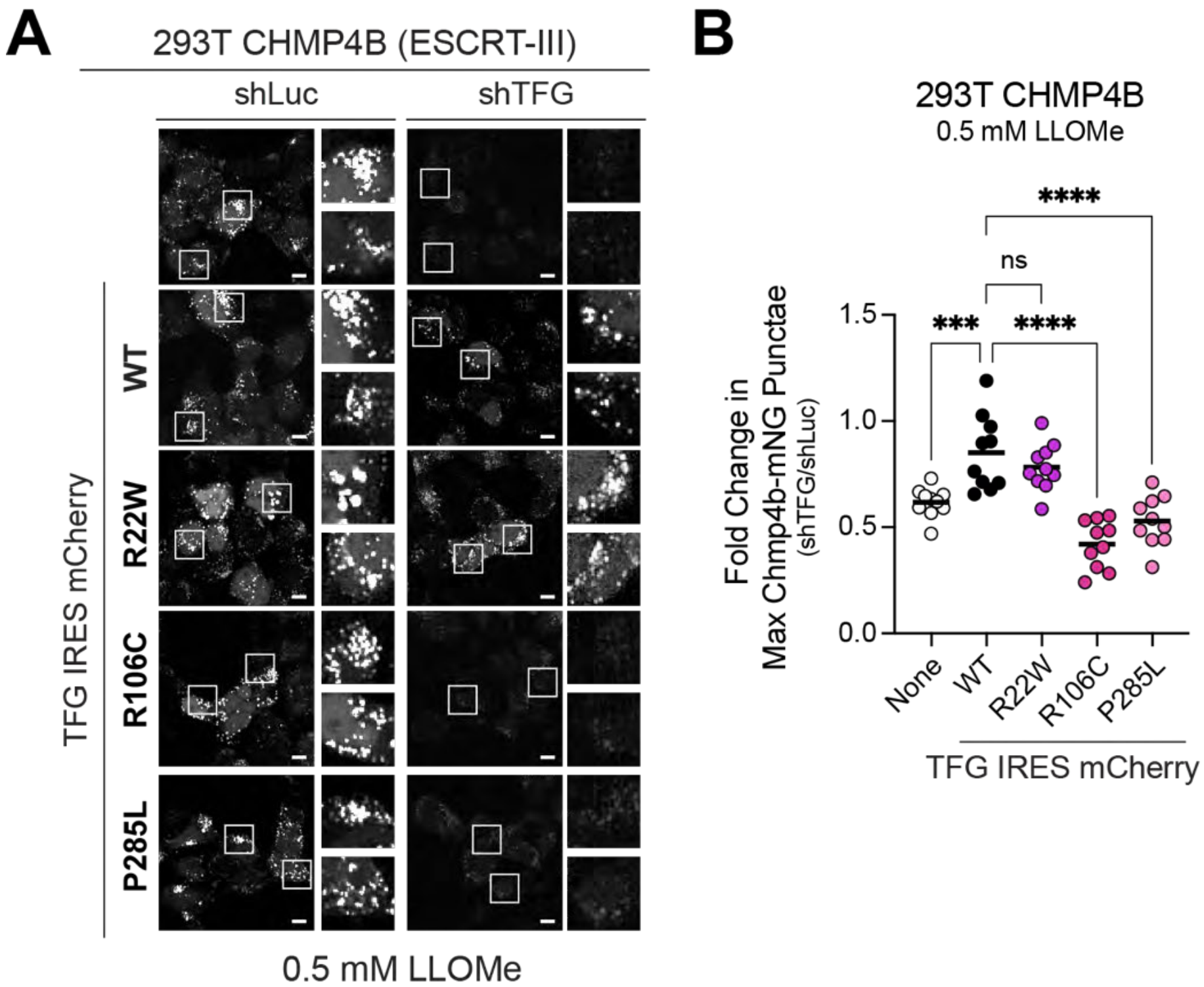

Reviewer Figure 17. Pathogenic P285L mutation of TFG, but not R22W mutation, leads to impaired ESCRT-III recruitment to damaged lysosomes.
(**A, B**) Representative confocal images (**A**) and quantification (**B**) of maximum CHMP4B-mNG puncta in 293T cells treated with DMSO (vehicle control) or 0.5 mM LLOMe following knockdown with shRNA targeting Luc or TFG and reconstituted with either wild-type or R22W, R106C, or P285L mutant TFG proteins. Images are shown for 25 minutes of treatment. N = 10 FOVs. Scale bars, 10 µm.

3. TFG function in NPC1KO cells
Since TFG was identified in NPC1KO cells, it would be interesting to know whether its role is more critical in this background than in WT cells. Specifically, did the authors compare the severity of ESCRT recruitment defects and lysosomal damage in the absence of TFG between WT and NPC1KO cells?

Our functional experiments in wild-type versus NPC1-KO 293T cells show a similar degree of dependency on TFG for ESCRT-III recruitment, which might be due to the specific experimental conditions versus the screen (i.e. degree of TFG knockdown, etc.). However, we did find an interesting qualitative difference in the membrane repair mechanism between wild-type and NPC1-KO cells. Specifically, whereas ALIX was entirely dispensable for ESCRT-III recruitment in wild-type cells, it was required in NPC1-KO cells (**Reviewer Fig. 18; Fig. 1G-1H; Extended Data Fig. 9A-9C**). We note that ALIX recruitment is also TFG dependent in both wild-type and NPC1-KO cells (**Extended Data Fig. 6C-6D, 9F-9G**). The dual dependency of TFG and ALIX could explain why TFG was identified in the NPC1-KO K562 cells in the CRISPRi screen.

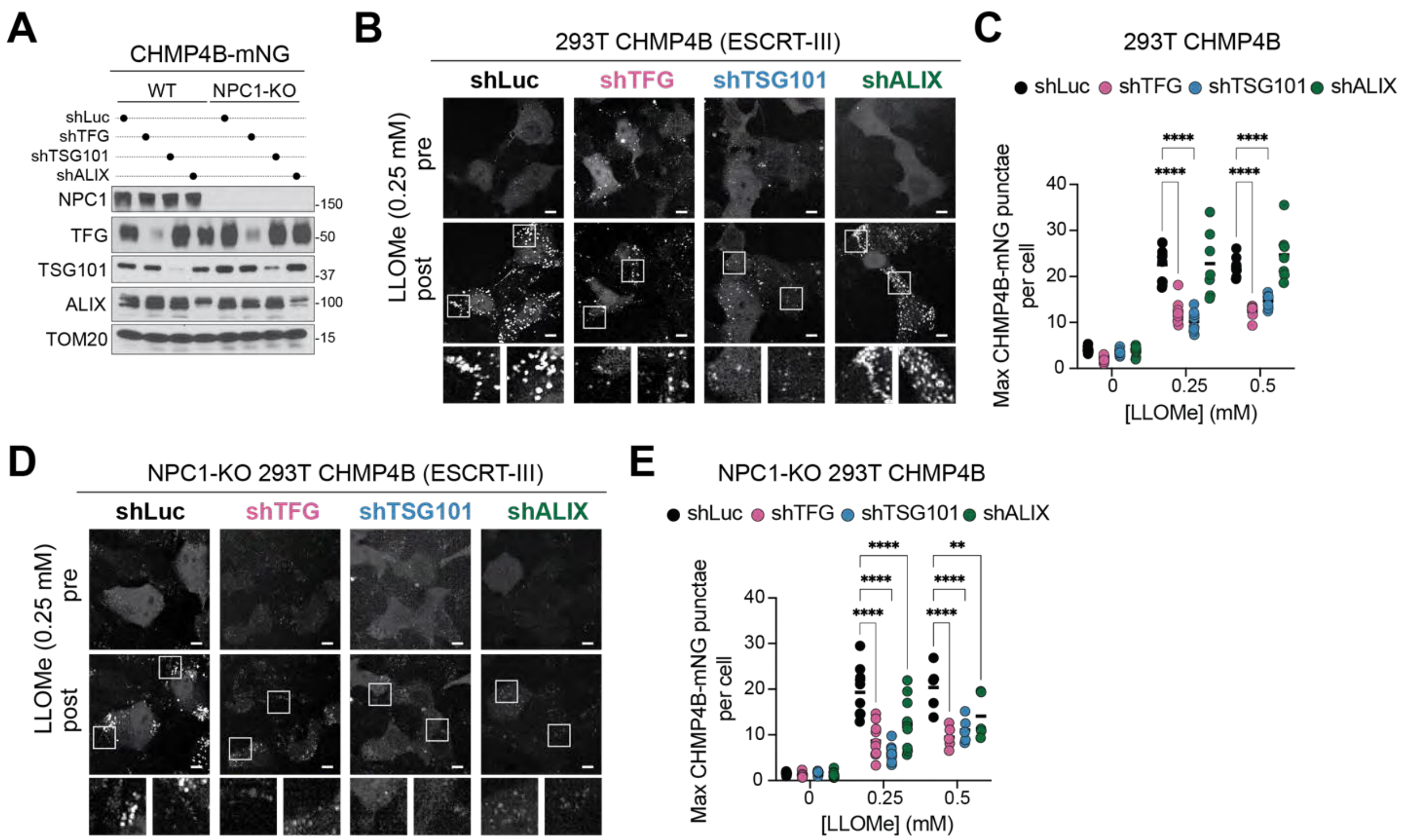

Reviewer Figure 18. TFG depletion phenocopies TSG101 loss and is obligate for ESCRT-III recruitment, whereas ALIX requirement is context-specific.
(**A**) Western blot analysis of wild-type (WT) or NPC1 knockout (KO) CHMP4B-mNG expressing 293T cells following knockdown with shRNAs targeting Luciferase (Luc) control, TFG, TSG101, or ALIX.
(**B, C**) Representative confocal images (**B**) and quantification **(C)** of maximum CHMP4B-mNG puncta formed in 293T cells treated with DMSO (vehicle control), 0.25 mM, or 0.5 mM LLOMe following treatment with shRNA targeting TFG (pink), TSG101 (blue), or ALIX (green), compared to Luc control (black). Images are shown for 5 minutes of treatment. N = 8 FOVs. Scale bars, 10 µm.
(**D, E**) Representative confocal images **(D)** and quantification **(E)** of maximum CHMP4B-mNG puncta formed in 293T NPC1 KO cells treated with DMSO (vehicle control), 0.25 mM, or 0.5 mM LLOMe following treatment with shRNA targeting TFG (pink), TSG101 (blue), or ALIX (green), compared to Luc control (black). Images are shown for 5 minutes of treatment. N = 9 FOVs. Scale bars, 10 µm.
Comparison of groups carried out by two-way ANOVA (**C, E**). Error bars represent mean +/- SD. **** p<0.001, **p<0.01.

4. Biochemical validation of TFG–ATG8 interaction
An in vitro pulldown experiment could validate the interaction between TFG and ATG8 proteins and clarify the requirement of the LIR2 motif. Notably, a previous study (PMID: 33932238) reported that LIR3 mediates this interaction, raising a potential inconsistency. It would be valuable for the authors to address this issue experimentally or in discussion.

We thank the reviewer for raising this important point. We assessed whether TFG and ATG8 could directly interact in an equilibrium binding assay. We purified a truncated mCherry-TFG$^{1-210}$ (the full length, including the C-terminal intrinsically disordered region, could not be purified due to aggregation and precipitation), both wild-type and LIR2-mutant, and combined it with agarose beads coated with Flag-GFP-LC3B. Recombinant wild-type TFG exhibited detectable binding to bead-bound LC3B (**Reviewer Fig. 14A-14B**). Despite identical expression and stability to wild-type (ruling out destabilizing effects of the LIR2 mutation) (**Reviewer Fig. 14C**), the LIR2 mutant of TFG$^{1-210}$ failed to display any detectable binding to LC3B (**Reviewer Fig. 14A-14B**).

In line with the lower affinity of the TFG-LIR2 peptide for LC3B versus the affinity of the p62-LIR peptide (~60 µM for TFG-LIR2 versus ~2 µM for p62) (**Extended Data Fig 18A-18D**), the binding of wild-type mCherry-TFG$^{1-210}$ to LC3B containing beads was much weaker than the

same amount of mCherry-p62 (**Reviewer Fig. 14C-14D**). Despite this weak TFG-LC3B affinity under these *in vitro* conditions, this interaction is clearly critical for TFG recruitment and subsequent ESCRT assembly and lysosomal repair as further shown by our new data in the HSP patient fibroblasts (**Reviewer Fig. 13A-13D above; Fig. 5E-5H**).

Regarding LIR3, we have tested it in peptide binding and knockdown rescue experiments and found no measurable interaction with either LC3B or GABARAP (**Extended Data Fig. 18C-18D**) and no requirement for TFG or ESCRT-III recruitment to damaged lysosomes (**Fig. 3D-3G; Extended Data Fig. 18J-18L**). The reported interaction with LC3C might relate to a different ATG8-dependent process than lysosome repair.

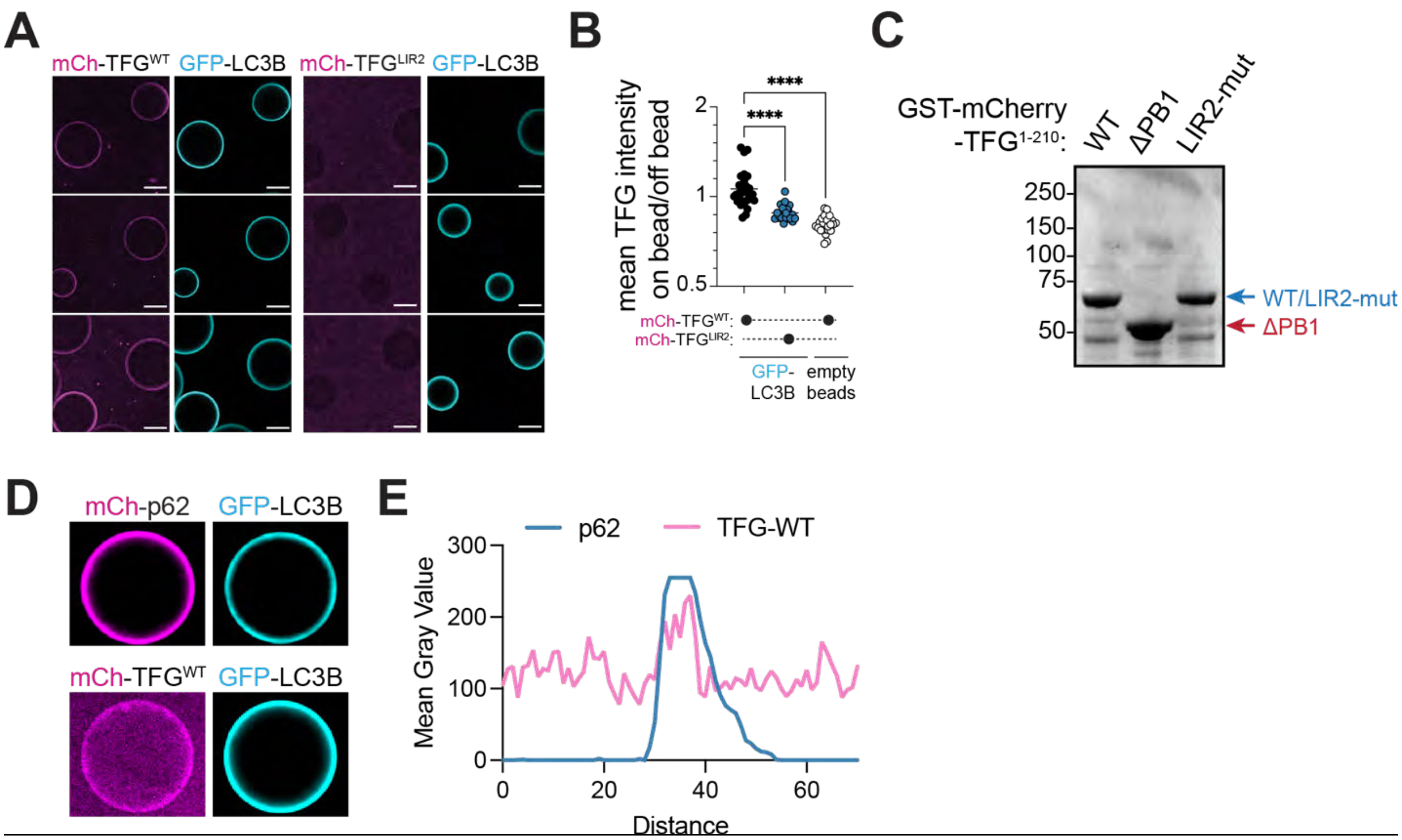

Reviewer Figure 14. Recombinant TFG$^{1\text{-}210}$ binds to LC3B in vitro in a LIR2-dependent manner, but with lower affinity than p62.

(**A, B**) Representative confocal images (**A**) and quantification (**B**) of binding between recombinant mCherry-TFG$^{1\text{-}210}$, wild-type (WT; *left*) or LIR2-mutant (*right*), and GFP-trap beads coated with Flag-GFP-LC3B. Quantification of 16-30 individual beads.

(**C**) Coomassie staining of purified recombinant 6xHis-mCherry-TFG$^{1\text{-}210}$ WT versus mutant (ΔPB1 and LIR2-mut) proteins.

(**D, E**) Representative confocal images (**D**) and line graph quantification (**E**) of bead bound mCherry-p62 (*top*) versus mCherry-TFG$^{1\text{-}210}$ WT (*bottom*) recombinant proteins. GFP-trap beads were coated with Flag-GFP-LC3B.

Minor Comments and Suggestions for Presentation

1. Starting from Figure 1e, lysosomal damage and factor recruitment are visualized as Galectin-3 puncta or other foci. While lysosome specialists can interpret these as bona fide damage markers, general readers may confuse them with protein aggregates. Including co-localization with lysosomal markers early in the figures would improve clarity.

We now provide colocalization data between endogenous lysosomal markers (LAMTOR4 and LAMP2) and mCherry-Galectin-3 as well as CHMP4B-mNG (**Reviewer Fig. 19; Extended Data Fig. 4C-4D, 5A-5B**).

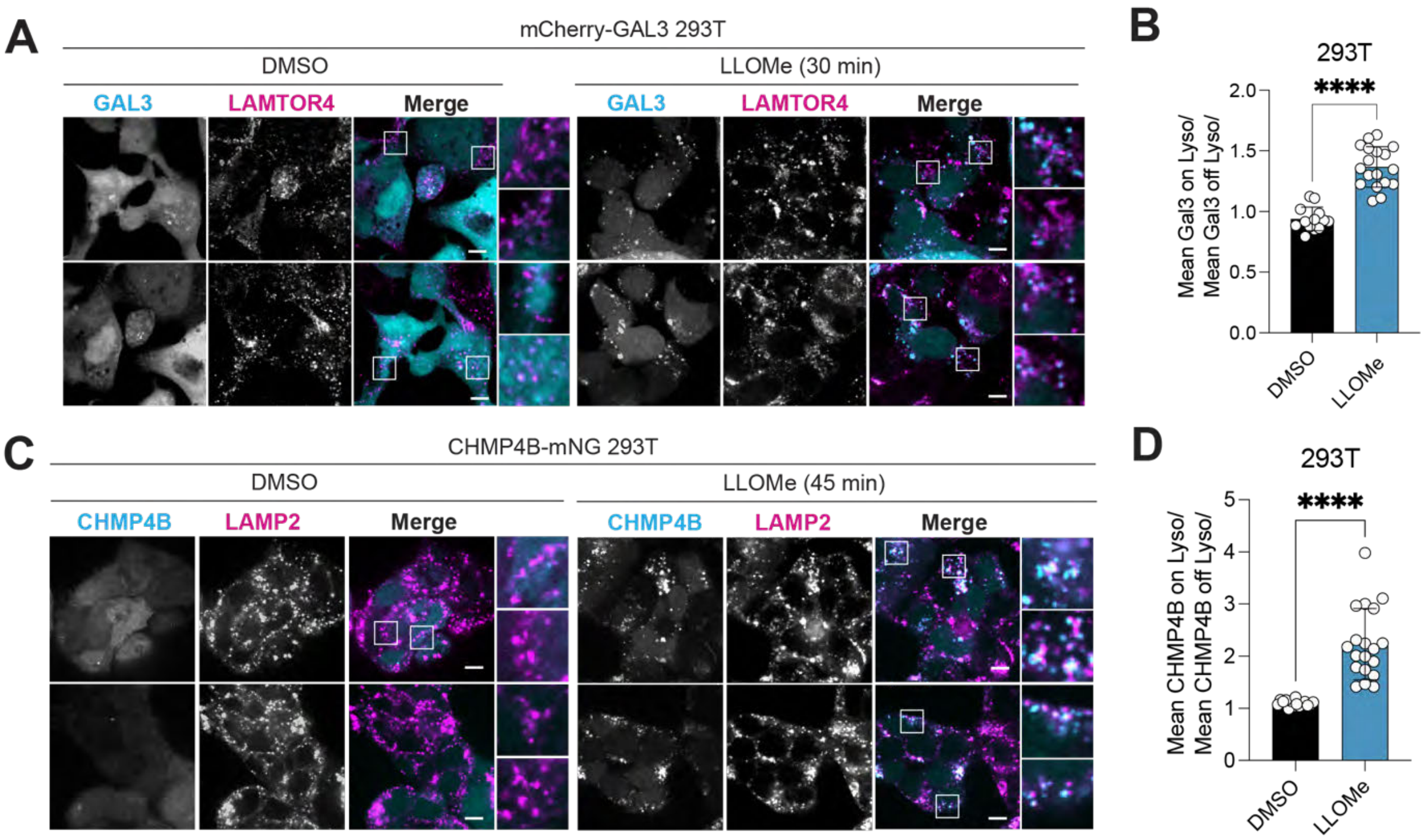

Reviewer Figure 19. mCherry-Galectin-3 and CHMP4B-mNG co-localize with lysosomes.
(**A, B**) Representative confocal images (**A**) of mCherry-Galectin-3 and LAMTOR4 (lysosomal marker) immunofluorescence staining and quantification (**B**) of colocalization ratio between Gal-3-mCherry puncta and LAMTOR4 in 293T cells. Cells were treated with vehicle control (DMSO) or 0.25 mM LLOMe for 30 minutes. N = 18 individual cells. Scale bars, 10 µm.
(**C, D**) Representative confocal images (**C**) of CHMP4B-mNG and LAMP2 (lysosomal marker) immunofluorescence staining and quantification (**D**) of colocalization ratio between CHMP4B-mNG puncta and LAMP2 in 293T cells. Cells were treated with vehicle control (DMSO) or 0.25 mM LLOMe for 45 minutes. N = 20 individual cells. Scale bars, 10 µm.

2. In Figures 1b and S1C,D, the y-axis is labeled "Rho," but the legends describe p-values. This is confusing and should be corrected.

We thank the reviewer of pointing out this mistake. We have now corrected the figure legends to say ρ-values instead of p-values.

3. In Figure 1b, TFG should be highlighted for emphasis.

TFG is now included in the scatter plot in Fig. 1B.

4. In Figures S1C,D, I suggest using both text color (black/blue) and spot color to highlight the identified genes for clarity.

Text coloring is now corrected in Extended Data Fig. 1C-1D so represent the gene hits.

5. Figure 5A: The ΔPB mutant shows puncta resembling liquid droplets at 5 and 2.5 μM TFG. The authors should clarify this phenotype.

We have replaced the images shown for 5 and 2.5 μM for the ΔPB mutant in the original Fig. 5A (now **Extended Data Fig. 20A**) with representative images that better reflect the typical

appearance of the beads observed across the dataset. The original images were of smaller beads, which appeared droplet-like when fully coated, giving the misleading impression of liquid-phase-like puncta. In the revised figure, larger beads are shown, making it clear that the structures correspond to coated beads rather than liquid droplets.

6. In Figure 3, specify the exact amino acid substitutions in the LIR mutants for accessibility to broader readers.

We have now updated the figure legends for any figure that used LIR2 or LIR3 mutant TFG proteins to reflect the mutation made: SAFDPL > SAADPA for LIR2 and PGFTSL > PGATSA for LIR3.

References

1. Skowyra, M. L., Schlesinger, P. H., Naismith, T. V. & Hanson, P. I. Triggered recruitment of ESCRT machinery promotes endolysosomal repair. *Science* **360**, eaar5078 (2018).
2. Radulovic, M. *et al.* ESCRT-mediated lysosome repair precedes lysophagy and promotes cell survival. *EMBO J* **37**, e99753 (2018).
3. Yim, W. W.-Y., Yamamoto, H. & Mizushima, N. Annexins A1 and A2 are recruited to larger lysosomal injuries independently of ESCRTs to promote repair. *FEBS Lett* **596**, 991–1003 (2022).
4. Tan, J. X. & Finkel, T. A phosphoinositide signalling pathway mediates rapid lysosomal repair. *Nature* **609**, 815–821 (2022).
5. Goodwin, J. M. *et al.* GABARAP sequesters the FLCN-FNIP tumor suppressor complex to couple autophagy with lysosomal biogenesis. *Sci Adv* **7**, eabj2485 (2021).
6. Zhang, Z. *et al.* Multiple roles for TFG ring complexes in neuronal cargo trafficking. *bioRxiv* 2024.11.05.621662 (2024) doi:10.1101/2024.11.05.621662.
7. Slosarek, E. L. *et al.* Pathogenic TFG Mutations Underlying Hereditary Spastic Paraplegia Impair Secretory Protein Trafficking and Axon Fasciculation. *Cell Rep* **24**, 2248–2260 (2018).
8. Elsayed, L. E. O. *et al.* Hereditary spastic paraplegias: identification of a novel SPG57 variant affecting TFG oligomerization and description of HSP subtypes in Sudan. *Eur J Hum Genet* **25**, 100–110 (2016).

Referee #1 (Remarks to the Author):

The authors fully addressed my previous comments. I support publication.

Although ESCRT has been known to play a key role in lysosomal repair since 2018, how ESCRT is recruited to damaged lysosomes has been an essential unanswered question in the field. The revised manuscript identifies TFG as a key mediator of ESCRT recruitment, providing a convincing mechanistic link between calcium-dependent noncanonical autophagy (also known as CASM, conjugation of ATG8 to single membrane) and ESCRT-mediated lysosomal repair.

We thank the Reviewer for their support of our manuscript and acknowledgement of its novelty and significance.

Referee #2 (Remarks to the Author):

The authors have successfully addressed most of the points I raised, and the revision has significantly improved the quality of the manuscript. Nevertheless, two major concerns remain:

We appreciate the Reviewer's recognition that the revised manuscript substantially strengthens the mechanistic and conceptual framework of the study.

1. The recent paper by Corkery et al. (ref. 26) shows that ESCRT recruitment to damaged lysosomes can occur even in the absence of CASM, although CASM makes recruitment more efficient. Thus, LASER only represents one out of several alternative mechanisms for ESCRT recruitment to damaged lysosomes, and two such mechanisms have already been described previously. This reduces the priority for publication in Nature.

With respect to previous studies, Ogura et al proposed that an interaction between the ATG8 protein, GABARAPL2, and ALIX promotes recruitment of the latter to damaged lysosomes, thus leading to ESCRT assembly. However, evidence by us and several others points to ALIX as playing an accessory, non-essential role in ESCRT recruitment to damaged lysosomes[1,2] (**new Extended Data Fig. 7I-7Q**). The data by Corkery et al supports the strict requirement for ATG5-ATG12 in the recruitment of ESCRT subunits, while hinting that this complex may play additional roles, independent of ATG8 conjugation, in promoting ESCRT assembly.

These data notwithstanding, neither study identified a molecular adaptor that directly links CASM-dependent ATG8 conjugation, TSG101 recruitment and the resulting ESCRT-I polymerization. Our work fills this precise mechanistic gap by defining LASER and positioning TFG as the missing biochemical bridge between ATG8-decorated lysosomes and ESCRT-I recruitment. This represents a conceptual advance beyond demonstrating requirement or partial independence of CASM, as it establishes the molecular logic by which damage sensing is rapidly converted into ESCRT assembly.

Our mechanistic dissection of pathogenic TFG mutations also links impaired LASER function to defective lysosomal resealing in a neurodegenerative context, thereby extending the impact of this pathway beyond fundamental cell biology into disease mechanism.

2. The mechanisms for TFG recruitment to damaged lysosomes and for prevention of ESCRT recruitment to ER exit sites (my original comment no. 3) have still not been fully addressed. It makes sense that calcium release from damaged lysosomes is a trigger of LASER recruitment since it is known to induce CASM. It is also interesting that VAP proteins are involved, but the molecular mechanism between CASM, TFG release from ER exit sites and LASER recruitment to damaged lysosomes is still not resolved.

Our revision experiments delineated a spatially organized cascade for TFG recruitment to the lysosome. In this mechanism, lysosomal calcium release induces CASM on lysosomes that are kept in physical proximity to ER exit sites via VAP-dependent ER-lysosome contacts. This proximity enables lysosome-conjugated ATG8 proteins to directly bind to TFG via its LIR2 motif, driving its relocalization to lysosomes. Importantly, VAPA/B depletion prevents TFG recruitment to damaged lysosomes, demonstrating that TFG mobilization and lysosomal engagement are mechanistically coupled and dependent on ER–lysosome contact formation. This chain of events provides a sufficient mechanistic link between CASM induction, TFG recruitment and ESCRT polymerization.

Regarding the lack of ESCRT recruitment to ERES, the authors speculate that ERES lack cofactors that enable ESCRT recruitment to damaged lysosomes. They need to provide experimental data that support this hypothesis.

With respect to why ESCRT is not recruited to ERES, we believe that fully answering this question extends beyond the scope of the present study. Our data establish that TFG recruitment to lysosomes requires ATG8 engagement in the context of damage and ER–lysosome proximity, indicating that membrane context and CASM-dependent cues are critical determinants of LASER assembly and ESCRT activation.

Multiple non-mutually exclusive mechanisms could explain the absence of ESCRT recruitment at ERES, including:

(1) distinct oligomeric or biophysical states of TFG,
(2) lack of lysosome-specific cofactors, including ATG8 proteins,
(3) differential post-translational modification of TFG, and/or
(4) differences in membrane composition or curvature between ERES and lysosomes.

We now provide additional support for (1) and (2).

[Redacted text]

through new immunolocalization data we show that, in contrast to its clustering on damaged lysosomes, LC3B does not cluster on ERES under either baseline or LLOMe-treated conditions (**Reviewer Fig. 21**). Thus, a complete LASER is never assembled at ERES. Together with the AF TFG-LC3B prediction, these data strongly suggest that TFG can only engage TSG101 and trigger ESCRT polymerization in the context of a fully assembled LASER.

We believe that thoroughly dissecting these possibilities would require extensive additional investigation and would not alter the central conclusion that LASER functions as a damage-triggered, lysosome-specific ESCRT activation platform. We therefore believe that such analyses are better suited for future studies.

Minor comment:

Even though the authors now cite previous studies on CASM and ESCRT recruitment, they do not make full justice to these studies: "…the molecular links between lysosome-bound ATG8 proteins and ESCRT components remain incompletely understood", "We cannot rule out alternative, TFG-independent mechanisms

for ESCRT recruitment.” A more detailed description of the previous work is required, as is an integration of the LASER data with the data from previous studies.

In the revised Introduction and Discussion, we more explicitly integrate our findings with the studies by Corkery et al., Ogura et al., and related works, and clarify how LASER provides a mechanistic framework that reconciles these observations while providing a key conceptual advance.

[Figure and text redacted]

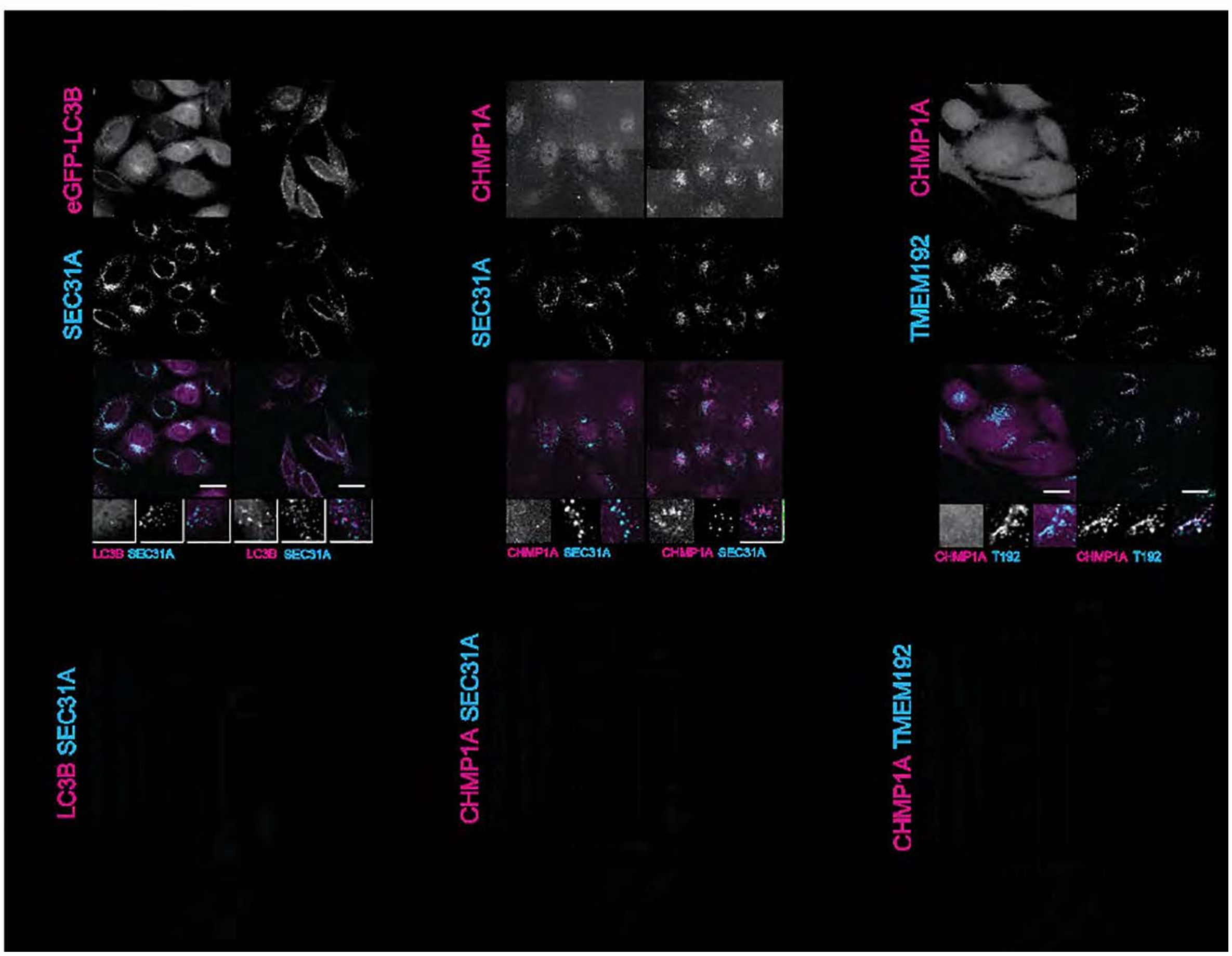

Reviewer Figure 21. LC3B and ESCRTs are not recruited to ERES.

(**A, B**) Representative confocal images (A) of eGFP-LC3B and SEC31A (ERES marker) immunofluorescence staining and quantification (B) of colocalization ratio in HeLa cells. Cells were treated with vehicle control (DMSO) or 0.5 mM LLOMe for 10 minutes. N=6 individual cells. Scale bars, 10 μm.
(**C, D**) Representative confocal images (C) of CHMP1A and SEC31A immunofluorescence staining and quantification (D) of colocalization ratio in HeLa cells. Cells were treated with vehicle control (DMSO) or 0.5 mM LLOMe for 10 minutes. N=5 individual cells. Scale bars, 10 μm.
(**E, F**) Representative confocal images (E) of CHMP1A and TMEM192 immunofluorescence staining and quantification (D) of colocalization ratio in HeLa cells. Cells were treated with vehicle control (DMSO) or 0.5 mM LLOMe for 5 minutes. N=3 individual cells. Scale bars, 10 μm.

Referee #3 (Remarks to the Author):

I co-reviewed this manuscript with one of the reviewers who provided the listed reports.

Referee #4 (Remarks to the Author):

The authors have carried out extensive experiments to address my questions, leading to a significant improvement in the quality of the work. I believe the manuscript is now ready for publication.

We are grateful to the Reviewer for their support of our findings and manuscript.

References

1. Skowyra, M. L., Schlesinger, P. H., Naismith, T. V. & Hanson, P. I. Triggered recruitment of ESCRT machinery promotes endolysosomal repair. *Science* **360**, eaar5078 (2018).
2. Radulovic, M. *et al.* ESCRT-mediated lysosome repair precedes lysophagy and promotes cell survival. *EMBO J.* **37**, e99753 (2018).
3. Zhang, Z. *et al.* Multiple roles for TFG ring complexes in neuronal cargo trafficking. *BioRxiv Prepr. Serv. Biol.* 2024.11.05.621662 (2024) doi:10.1101/2024.11.05.621662.
